# Mitochondrial reprogramming by activating OXPHOS via glutamine metabolism in African American patients with bladder cancer

**DOI:** 10.1172/jci.insight.172336

**Published:** 2024-09-10

**Authors:** Karthik Reddy Kami Reddy, Danthasinghe Waduge Badrajee Piyarathna, Jun Hyoung Park, Vasanta Putluri, Chandra Sekhar Amara, Abu Hena Mostafa Kamal, Jun Xu, Daniel Kraushaar, Shixia Huang, Sung Yun Jung, Livia S. Eberlin, Jabril R. Johnson, Rick A. Kittles, Leomar Y. Ballester, Krishna Parsawar, M. Minhaj Siddiqui, Jianjun Gao, Adriana Langer Gramer, Roni J. Bollag, Martha K. Terris, Yair Lotan, Chad J. Creighton, Seth P. Lerner, Arun Sreekumar, Benny Abraham Kaipparettu, Nagireddy Putluri

**Affiliations:** 1 Department of Molecular and Cellular Biology,; 2 Dan L Duncan Comprehensive Cancer Center,; 3 Department of Molecular and Human Genetics,; 4 Advanced Technology Cores,; 5 Huffington Department of Education, Innovation and Technology,; 6 Verna and Marrs McLean Department of Biochemistry and Molecular Pharmacology, and; 7 Department of Surgery, Baylor College of Medicine, Houston, Texas, USA.; 8 Department of Microbiology, Biochemistry, and Immunology, Morehouse School of Medicine, Atlanta, Georgia, USA.; 9 Department of Community Health and Preventive Medicine, Morehouse School of Medicine, Atlanta, Georgia, USA.; 10 Division of Pathology and Laboratory Medicine, The University of Texas MD Anderson Cancer Center, Houston, Texas, USA.; 11 Analytical and Biological Mass Spectrometry Core, University of Arizona, Tucson, Arizona, USA.; 12 Division of Urology, Department of Surgery, University of Maryland School of Medicine, Baltimore, Maryland, USA.; 13 Department of Genitourinary Medical Oncology, The University of Texas MD Anderson Cancer Center, Houston, Texas, USA.; 14 Georgia Cancer Center, Augusta University, Augusta, Georgia, USA.; 15 Department of Urology, Medical College of Georgia, Augusta, Georgia, USA.; 16 Department of Urology, University of Texas Southwestern Medical Center, Dallas, Texas, USA.; 17 Department of Medicine and; 18 Scott Department of Urology, Baylor College of Medicine, Houston, Texas, USA.

**Keywords:** Metabolism, Oncology, Cancer, Mitochondria, Urology

## Abstract

Bladder cancer (BLCA) mortality is higher in African American (AA) patients compared with European American (EA) patients, but the molecular mechanism underlying race-specific differences are unknown. To address this gap, we conducted comprehensive RNA-Seq, proteomics, and metabolomics analysis of BLCA tumors from AA and EA. Our findings reveal a distinct metabolic phenotype in AA BLCA characterized by elevated mitochondrial oxidative phosphorylation (OXPHOS), particularly through the activation of complex I. The results provide insight into the complex I activation–driven higher OXPHOS activity resulting in glutamine-mediated metabolic rewiring and increased disease progression, which was also confirmed by [U]13C-glutamine tracing. Mechanistic studies further demonstrate that knockdown of NDUFB8, one of the components of complex I in AA BLCA cells, resulted in reduced basal respiration, ATP production, GLS1 expression, and proliferation. Moreover, preclinical studies demonstrate the therapeutic potential of targeting complex I, as evidenced by decreased tumor growth in NDUFB8-depleted AA BLCA tumors. Additionally, genetic and pharmacological inhibition of GLS1 attenuated mitochondrial respiration rates and tumor growth potential in AA BLCA. Taken together, these findings provide insight into BLCA disparity for targeting GLS1-Complex I for future therapy.

## Abstract 2

Molecular landscape from bladder cancer patient tumors identified glutamine mediated metabolic rewiring by complex I activation in african american bladder cancer.

## Graphical abstract

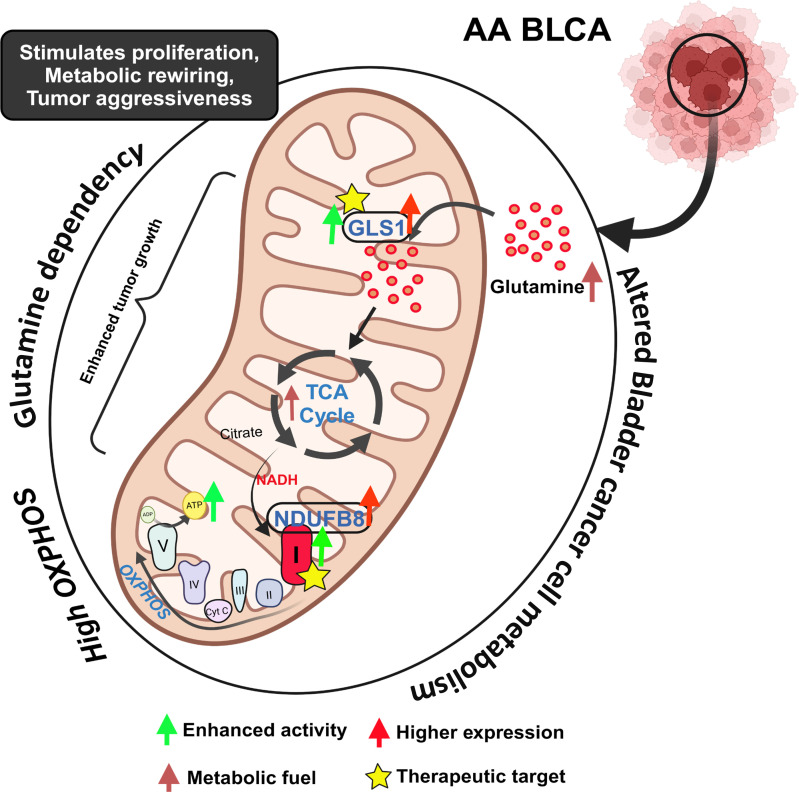

## Introduction

Bladder cancer (BLCA) is the second most prevalent urological cancer in the United States (1, 2). The survival of patients with BLCA is strongly influenced by demographic factors, geographic region, race/ethnicity, and environmental risk factors including tobacco and occupational exposures (3). According to the American Cancer Society (ACS), it is estimated that there will be 83,190 new BLCA cases diagnosed in 2024 (4). Surveillance, Epidemiology, and End Results (SEER) data show a decreased 5-year relative survival in African American (AA) patients with BLCA (58%–72%) compared with European American (EA) patients with BLCA (78%–84%) (5, 6). Notably, AA patients exhibit lower BLCA incidence rates compared with EA patients but have a higher mortality rate (7–11). The inferior survival of AA patients is driven by the different prevalence of risk factors, socioeconomic status (SES), equal access to health care (8, 11), and differential intrinsic tumor biology including altered metabolic activity (12–14). However, underlying molecular targets for the race-specific BLCA mortality are underexplored.

Using a pancancer data across multiple cancer types from The Cancer Genome Atlas (TCGA), we had previously identified enriched oxidative phosphorylation (OXPHOS) (15) in AA tumors compared with EA tumors including BLCA. Upregulation of genes associated with mitochondrial OXPHOS was identified in AA cancers (16). Earlier studies demonstrated that the alterations of metabolites (12, 13), lipids (17), and genes (18) in AA BLCA but the AA-specific BLCA targets were unexplored. Recent studies from our group and others in BLCA have documented the importance of key molecular and metabolic pathways in cancer progression (19–24). Despite the advancements in BLCA clinical management, molecular therapeutics targeting metabolic pathways and mitochondrial alterations have not been explored to improve poor disease outcomes among AA patients. We hypothesize that dysregulated mitochondrial energy that uses glutamine as a fuel drives enhanced disease progression and may be important for the treatment response in AA patients with BLCA.

In this study, using patient RNA-Seq, proteomics, and metabolomics data, we sought to investigate the role of dysregulated mitochondrial OXPHOS as a driver of disease progression in AA BLCA. Our aim was to identify a link between mitochondria-mediated metabolic rewiring in AA patients with BLCA with higher mortality rates.

## Results

### RNA-Seq, proteomics, and metabolomics data reveal an altered OXPHOS pathway in AA patients with BLCA.

**Figure 1:**
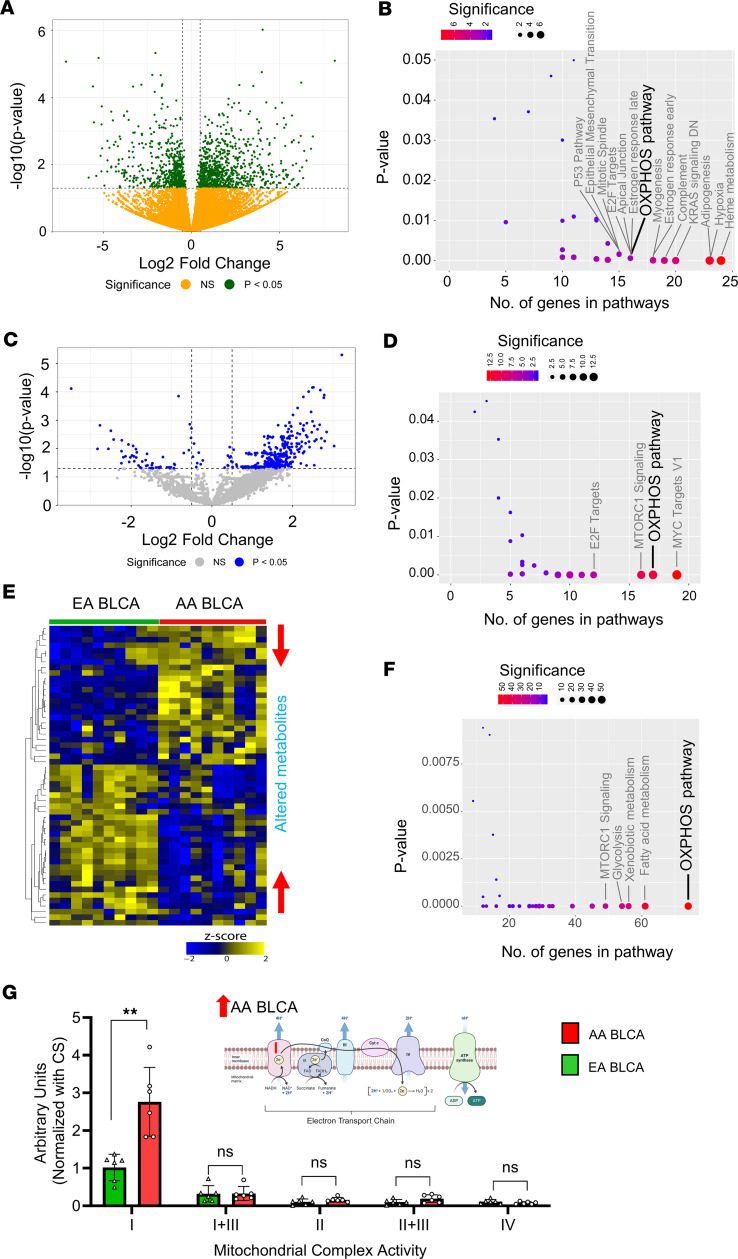
Identification of complex I–mediated OXPHOS activity in AA BLCA. (**A**) Volcano plot represents differentially expressed genes between AA BLCA (*n* = 13) and EA BLCA (*n* = 15) using RNA-Seq data (2-way comparison; *P* < 0.05 denoted as green dots; nonsignificant denoted as yellow dots). (**B**) Graphical representation of hallmark pathways obtained from RNA-Seq data (same data used in **A**) from AA patients versus EA patients with BLCA. Number of genes from pathways in *x* axis and significance in *y* axis are represented. Circle size correlates with number of genes (refer to Methods). (**C**) Volcano plot represents differentially expressed proteins between AA BLCA (*n* = 10) and EA BLCA (*n* = 12) from proteomics (2-way comparison; *P* < 0.05 denoted as blue dots; nonsignificant denoted as gray dots). Note: 4 matched patients with BLCA from RNA-Seq (**A**) and Proteomics (**C**). (**D**) Same as **B**, but BLCA proteomics data from AA patients versus EA patients with BLCA. Number of protein encoded genes from hallmark pathways in *x* axis and their significant *P* values (*P* < 0.05*;* refer to color scale) in *y* axis are represented. (**E**) Heatmap represents differentially expressed metabolites across BLCA tissues from AA (*n* = 10) and EA (*n* = 10) patients (FDR <0.25). Note: 4 matched patients with BLCA from Proteomics (**C**) and metabolomics (**E**). Upregulated metabolites are indicated in yellow, and downregulated are indicated in blue. (**F**) Graphical representation of enriched hallmark pathways obtained from metabolomics data (**E**) from AA BLCA versus EA patients with BLCA. Differential metabolites were mapped to genes and used for pathway analysis. Number of genes from pathways in *x* axis and their significant *P* values (*P* < 0.05*;* refer to color scale; circle size correlate with genes) in *y* axis are represented. (**G**) ETC activity measured in EA (*n* = 6) and AA (*n* = 6) BLCA tissues using colorimetric assay. Significance was determined by unpaired 2-tailed Student’s *t* test; **P < 0.01. Image was created with BioRender.com.

RNA-Seq (Transcriptomics), proteomics (by mass spectrometry [MS]), and metabolomics (targeted MS) were performed on deidentified flash-frozen tissues obtained from self-reported race AA/Black were defined as AA BLCA and self-reported race Caucasian/EA/White were defined as EA BLCA. Additionally, we used both AA and EA benign/normal tissues as a control for the RNA-Seq and proteomics profiling. Detailed clinical information (pathological stage, self-reported race, sex, and smoking status) for the samples were given in Supplemental Tables 1 and 2 (supplemental material available online with this article; https://doi.org/10.1172/jci.insight.172336DS1; refer to data availability section). Briefly, RNA-Seq includes AA BLCA (*n* = 13), EA BLCA (*n* = 15), AA benign/normal tissues (*n* = 6), and EA benign/normal tissue (*n* = 8); proteomics includes AA BLCA (*n* = 10), EA BLCA (n = 12), AA benign/normal tissues (*n* = 6), and EA benign/normal tissue (*n* = 7); and metabolomics includes AA BLCA (*n* = 10) and EA BLCA (*n* = 10) from multiple cohorts. RNA-Seq, proteomics analysis, and metabolomic analysis revealed that altered genes, proteins, and metabolites involved in OXPHOS are the key pathways in AA BLCA tumors, respectively (Figure 1, A–F; Supplemental Figure 1, A–F; and Supplemental Tables 3–5). The AA BLCA enriched OXPHOS pathway was also evident in TCGA-BLCA data (Supplemental Table 6) (15). Furthermore, we identified altered genes and proteins involved in OXPHOS and other key metabolic pathways (Supplemental Figure 1, C and F).

As shown in the Supplemental Figure 1, we utilized benign/normal bladder tissues as a control for RNA-Seq and proteomics profiling. Following this, we examined the variations in transcripts within each race compared with benign/normal and BLCA. In AA BLCA, a total of 992 genes exhibited statistically significant alterations relative to AA benign/normal tissues (Supplemental Figure 2A and Supplemental Table 7). A similar analysis was performed using EA BLCA compared with EA benign/normal tissues. As shown in Supplemental Figure 2B, the alterations of differentially expressed genes (DEGs) between EA BLCA and EA benign/normal represented as a volcano plot and the list of the altered genes were provided in Supplemental Table 8. Subsequently, we compared transcript levels between AA benign/normal and EA benign/normal tissues. We identified a set of DEGs between AA benign/normal and EA benign/normal tissues, as demonstrated in Supplemental Figure 2C, and list of the DEGs is provided in Supplemental Table 9.

To validate the AA BLCA–specific signature of genes from our RNA-Seq data, we have integrated DEGs between AA BLCA versus AA benign/normal and AA BLCA versus EA BLCA. In this analysis, we identified a set of upregulated and downregulated AA BLCA–specific genes (Supplemental Figure 2D and Supplemental Tables 7 and 10). The identified signature genes are based on the limited statistical power and need to be further validated.

Overall, RNA-Seq, proteomics, and metabolomics data provide a comprehensive view of the molecular alterations associated with the OXPHOS pathway in AA patients with BLCA (Figure 1, A–F) (15). These findings from the OXPHOS pathway shed light on the activity of subcomplex in that AA BLCA may be contributing toward tumor progression. To better understand the mitochondrial metabolic vulnerabilities in the context of racial disparity, a subset of AA BLCA and EA BLCA tumors (Supplemental Table 2) was used to perform the electron transport chain (ETC) activity assay, measuring the activity of subcomplexes. Our results reveal that the activity of mitochondrial complex I was exclusively higher in AA BLCA tumors (Figure 1G) and had no effect on complex I+III, II, II+III, and IV activity; this suggests that complex I may play a major role in greater energy production, biomass generation, and tumor growth.

### Targeting complex I inhibits tumor growth in AA BLCA.

**Figure 2:**
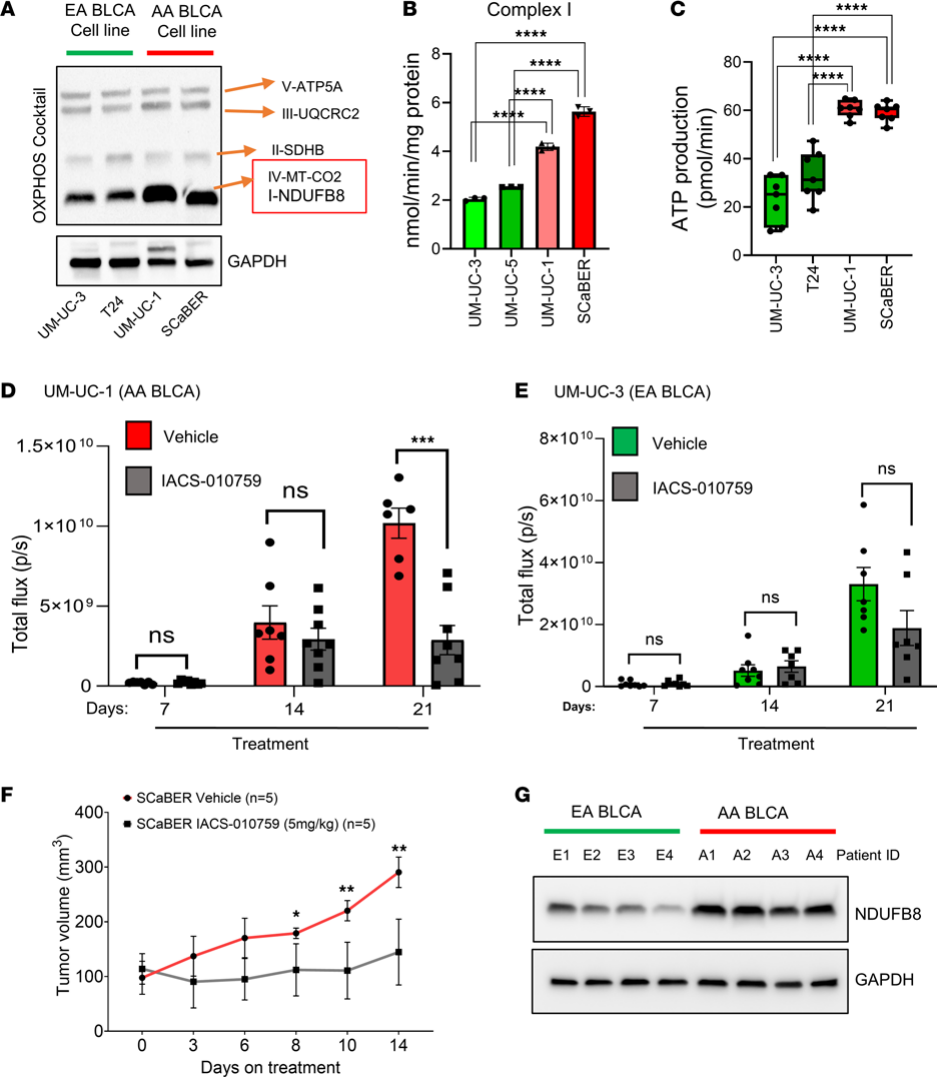
Pharmacological perturbation of complex I inhibition in AA BLCA. (**A**) Protein expression of OXPHOS cocktail — mitochondrial complex V (ATP5A), III (UQCRC2), II (SDHB), IV (MT-CO2), and I (NDUFB8) — in EA BLCA (UM-UC-3, T24) and AA BLCA (UM-UC-1, SCaBER) cell lines. (**B**) Mitochondrial complex activity in AA BLCA — UM-UC-1 (*n* = 3), SCaBER (*n* = 3) — cell lines compared with EA BLCA — UM-UC-3 (*n* = 3), UM-UC-5 (*n* = 3) — cell lines; optical density (OD) of complex I activity was measured using specific substrates and normalized with the citrate synthase activity measured by colorimetric assay (****P < 0.0001). (**C**) Box-and-whisker plot represents ATP production in AA BLCA — UM-UC-1 (*n* = 7), SCaBER (*n* = 7) — and EA BLCA — UM-UC-3 (*n* = 7), T24 (*n* = 7) — cell lines measured by Seahorse assay. Data were normalized using cell number measured by CellTiter-Glo (****P < 0.0001). (**D** and **E**) Scatter plot represents the tumor growth (orthotopic) of AA BLCA (UM-UC-1) and EA BLCA (UM-UC-3) upon IACS-010759 (5 mg/kg) treatment compared with vehicle control. Luciferase measurements were performed over a period of 21 days — UM-UC-1: days 7 and 14 for Control (*n* = 7), IACS-010759 treatment (*n* = 8); day 21 for Control (*n* = 6), IACS-010759 treatment (*n* = 8); and UM-UC-3: day 7 for Control (*n* = 8), IACS-010759 treatment ( *n* = 8); day 14 for Control (*n* = 8), IACS-010759 treatment (*n* = 7); day 21 for Control (*n* = 7), IACS-010759 treatment (*n* = 7) (unpaired *t* test for each time point; ***P < 0.001). (**F**) Plot represents the tumor growth (s.c.) from AA BLCA (SCaBER) cell line–derived xenografts treated with IACS-010759 (5 mg/kg; *n* = 5) compared with vehicle control (*n* = 5) (unpaired *t* test for each time point; **P < 0.01; *P < 0.05*)*. (**G**) Protein expression of NDUFB8 (mitochondrial complex I protein) in EA (*n* = 4; E1 to E4) and AA BLCA (*n* = 4; A1 to A4) tissues measured by Western blot analysis. Significance was determined by unpaired 2-tailed student *t* test.

Given the increase of mitochondrial activity in AA BLCA, we sought to determine whether AA tumor cells would generate a higher rate of ETC for compensation. Using ancestry-verified BLCA cell lines (Supplemental Table 11), we measured the expression of OXPHOS proteins. Western blot analysis of OXPHOS protein showed increased expression of complex I (NDUFB8) and complex IV (MT-CO2) in AA BLCA compared with EA BLCA cell lines (Figure 2A). Furthermore, we measured the ETC activity in AA and EA BLCA cell lines. Since we found that AA patients with BLCA had a higher complex I activity, we observed that AA BLCA cell lines also had higher complex I activity (Figure 2B). In addition, we also observed elevated activity of other complexes in AA BLCA cell lines (Supplemental Figure 3A), but we did not observe the alterations of other complex activities in AA patients with BLCA (Figure 1G). Upon observing high complex I activity in AA BLCA tumors and AA BLCA cell lines, we further measured ATP production from the above AA BLCA and EA BLCA cell lines using the Seahorse XF Cell Mito Stress assay. The Seahorse assay results reveal a higher ATP production in AA BLCA cell lines (UM-UC-1 and SCaBER) compared with EA BLCA cell lines (UM-UC-3 and T24) (Figure 2C). In line with above findings, our analysis from patient tumors identified the activation of mitochondrial complex I activity in AA BLCA, which may explain race-based disparities in BLCA oncologic outcomes and pave the way for the development of race-specific biomarkers and therapeutics.

To understand the effects of increased complex I activity in AA BLCA, we used IACS-010759, a specific mitochondrial complex I inhibitor (25) used in clinical trial (26), to test the effect on tumor progression in an in vivo orthotopic model. We engrafted AA-derived (UM-UC-1) BLCA cells and EA-derived (UM-UC-3) BLCA cells into the bladder wall of NOD-SCID-γ (NSG) mice. After orthotopic injection, each mouse was imaged by bioluminescence imaging (BLI) once a week, and weights were monitored. Luciferase signals were observed in mice after 1 week, indicating initial growth of the BLCA cells. At day 7, we randomized the mice using luciferase readings to test the efficacy of IACS-010759 (5 mg/kg; oral administration). At day 21, AA-derived bladder tumors treated with IACS-010759 showed prominent inhibition of tumor growth (Figure 2D) compared with EA BLCA orthotopic tumors (Figure 2E). During all of these experiments, we observed no loss of mice weight in the vehicle and treatment groups (Supplemental Figure 3, D and E). Furthermore, we also validated these findings in another AA BLCA cell line (SCaBER) using a s.c. model. Our results show that IACS-010759 treatment significantly inhibited tumor growth compared with the vehicle control (Figure 2F).

### Complex I activation drives tumorigenesis via increased OXPHOS and glutamine dependency in AA BLCA.

**Figure 3:**
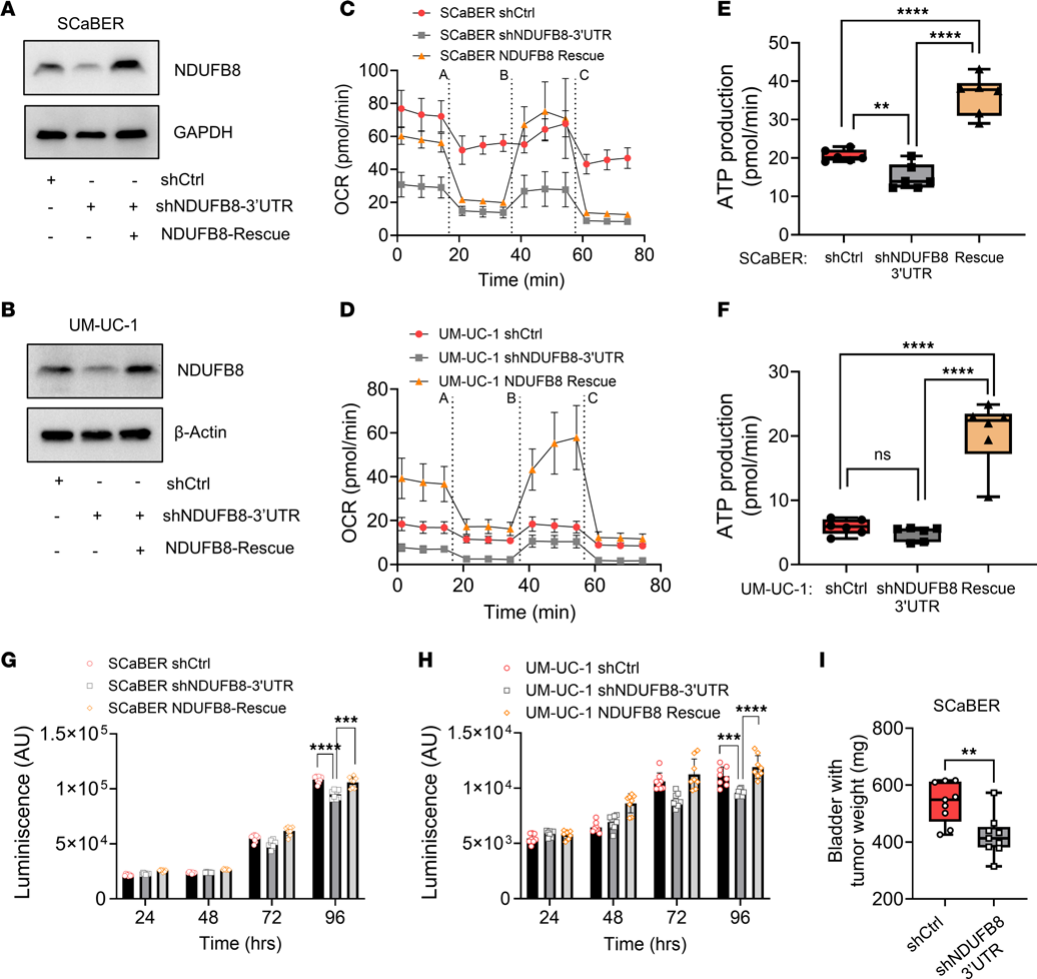
Complex I–mediated (NDUFB8-mediated) OXPHOS activity, and tumor growth in AA BLCA. (**A** and **B**) Immunoblot analysis showed the confirmation of NDUFB8 KD and reexpression using the full-length NDUFB8 in the KD (shNDUFB8-3′UTR) background cells (rescue) in SCaBER and UM-UC-1 compared with their corresponding nontargeting control. (**C** and **D**) KD of NDUFB8 (*n* = 6) in SCaBER and UM-UC-1 significantly reduces basal respiration compared with shControl (*n* = 6) (oxygen consumption rate [OCR]) and rescued upon NDUFB8 reexpression (*n* = 6) measured by Seahorse assay (A, oligomycin; B, FCCP; C, Rotenone/Antimycin A; data are normalized with cell number by counting). (**E** and **F**) Same as in **C** and **D**, but for ATP production in SCaBER and UM-UC-1 cell lines, respectively (****P < 0.0001, ** P < 0.01). (**G**) CellTiter-Glo proliferation assay significantly reduced in SCaBER NDUFB8 KD (*n* = 8) compared with shControl (*n* = 8) and rescued upon NDUFB8 reexpression (*n* = 8) (***P < 0.001; ****P < 0.0001). (**H**) CellTiter-Glo proliferation assay significantly reduced in UM-UC-1 NDUFB8 KD (*n* = 8) compared with shControl (*n* = 8) and rescued upon NDUFB8 reexpression (*n* = 8) (***P < 0.001; ****P < 0.0001). (**I**) Weight of the orthotopic mice bladder harboring tumors (endpoint: day 35) from SCaBER shCtrl (*n* = 9) and SCaBER shNDUFB8-3′UTR (*n* = 9) (**P < 0.01). Significance was determined by unpaired 2-tailed Student’s *t* test.

Complex I is the component of respiratory chain and is composed of various modules with different subunit proteins (27, 28). To further investigate ETC expression in AA BLCA, we have measured the protein expression of complex I (NADH Dehydrogenase [Ubiquinone] 1 Beta Subcomplex Subunit 8-NDUFB8); NADH: Ubiquinone Oxidoreductase Subunit A6-NDUFA6; NADH Dehydrogenase (Ubiquinone) Flavoprotein 1-NDUFV1); complex II (Succinate Dehydrogenase Complex, Subunit B-SDHB); complex III (Ubiquinol-Cytochrome C Reductase Complex Core Protein 2-UQCRC2); complex IV (Mitochondrially Encoded Cytochrome C Oxidase II-MT-CO2); and complex V (ATP Synthase F1 Subunit Alpha-ATP5A) subunits and identified that NDUFB8 levels are higher in AA BLCA (Figure 2G and Supplemental Figure 3F) but not the other complex proteins (Supplemental Figure 3B). In line with our results, AA BLCA cell lines exhibit higher NDUFB8 expression compared with EA BLCA cell lines (Supplemental Figure 3C). We hypothesized that components of the mitochondrial complex could be one of the key mediators for AA BLCA progression. To validate the function of complex I, we used NDUFB8 as a surrogate gene of complex I. We have knocked down NDUFB8 expression using shRNA (2 independent sequences; one targeting CDS region and other 3′UTR region) and used nontargeting scrambled shRNA as a control. We further rescued NDUFB8 expression with an overexpression construct in shNDUFB8-3′UTR (Supplemental Table 12) for AA (SCaBER and UM-UC-1) (Figure 3, A and B, and Supplemental Figure 4A) and EA BLCA cells (UM-UC-3) (Supplemental Figure 4, E and I). We measured the mitochondrial respiration using the Seahorse assay in AA BLCA and EA BLCA cell lines, examining basal respiration (oxygen consumption rate [OCR], extracellular acidification rate [ECAR]), and ATP production in NDUFB8-knockdown (NDUFB8-KD) and rescue cells compared with control cells (Figure 3, C and D; Supplemental Figure 4, B, D, F, H, J, and L; and Supplemental Figure 5, A and B). As expected, ATP production is decreased upon NDUFB8 KD and increased upon rescued expression of NDUFB8 (Figure 3, E and F, and Supplemental Figure 4, C, G, and K). Furthermore, we observed decreased proliferation in NDUFB8-KD cells compared with control cells and rescued upon NDUFB8 reexpression (Figure 3, G and H, and Supplemental Figure 4, M–O). As stated above, we observed higher NDUFB8 in AA BLCA. We then generated orthotopic xenograft using 2 independent clones of NDUFB8 KD in AA BLCA cell lines to measure the tumor growth. In vivo results reveal that NDUFB8 KD had a significant effect on tumor regression in 2 independent clones compared with controls (Figure 3I and Supplemental Figure 5C).

### OXPHOS-mediated rewired glutamine metabolism in AA BLCA.

**Figure 4:**
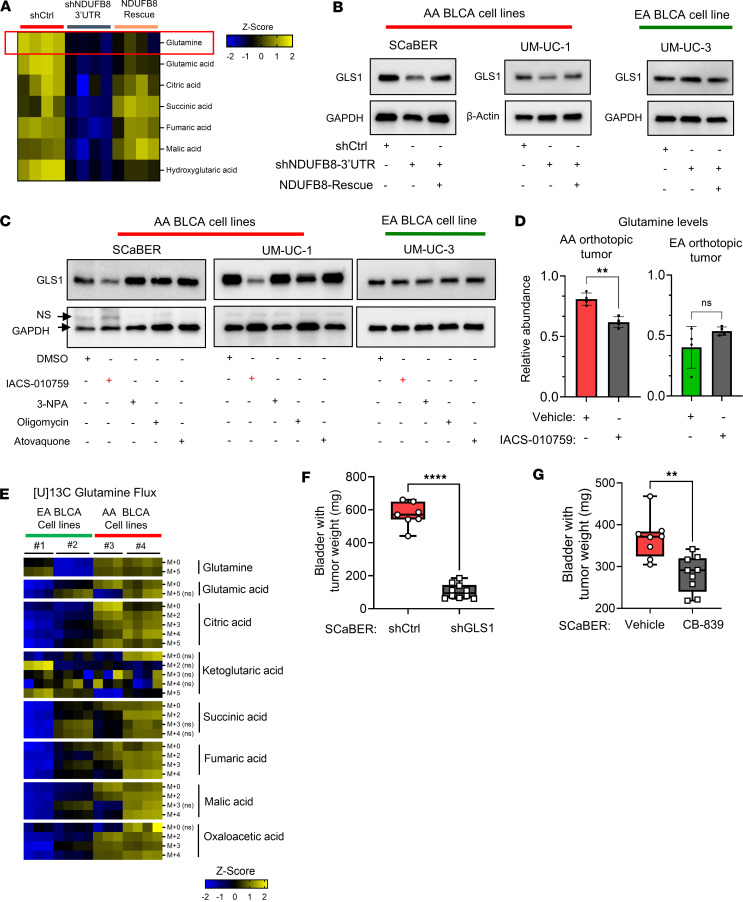
Genetic perturbations of NDUFB8 affects GLS1 and glutamine-mediated mitochondrial metabolism in AA BLCA. (**A**) Heatmap represent the levels of TCA intermediates in shCtrl (*n* = 4), NDUFB8 KD (*n* = 4), and NDUFB8 rescue (*n* = 4) AA (SCaBER) BLCA cell lines measured by LC-MS (FDR < 0.25). (**B**) Western blot analysis represents the expression of GLS1 in AA BLCA (SCaBER and UM-UC-1) and EA BLCA (UM-UC-3) cell lines with NDUFB8 KD and rescue. (**C**) Western blot analysis represents the expression of GLS1 in AA BLCA (SCaBER and UM-UC-1) and EA BLCA (UM-UC-3) cell lines treated with mitochondrial complex specific inhibitors — IACS-010759-10 μM, 3-Nitropropionic acid (3-NPA)-10 μM, oligomycin-10 μM, atovaquone-10 μM. (**D**) Relative levels of glutamine (normalized with internal standard) measured by LC-MS in AA BLCA orthotopic tumors (UM-UC-1) and EA BLCA orthotopic tumors (UM-UC-3) treated with IACS-010759 (5 mg/kg; *n* = 4) compared with vehicle (*n* = 4). Significance was determined using unpaired 2-tailed Student’s *t* test; **P < 0.01. (**E**) Heatmap representing [U]13C glutamine incorporation (6 hours) into TCA cycle of EA BLCA — #1=UM-UC-3 (*n* = 3), #2=J82 (*n* = 4) — and AA BLCA — #3=UM-UC-1 (*n* = 3), #4=SCaBER (*n* = 4) — cell lines. Peak areas are converted to log_2_ and followed by *z* score transformation. Significance was determined based on log-transformed data using Student’s *t* test between AA BLCA cell lines and EA BLCA cell lines. Yellow represents increased, and blue represent decreased levels from *Z* score values. (**F**) Weight of the orthotopic mice bladder harboring tumors (endpoint: day 37) from SCaBER shCtrl (*n* = 7) and GLS1 KD (*n* = 10) tumors. Significance was determined by unpaired 2-tailed Student’s *t* test; ****P < 0.0001. (**G**) Weight of the orthotopic mice bladder harboring tumors derived from SCaBER (endpoint: day 31) treated with vehicle (*n* = 8) and CB-839 (*n* = 9). Significance was determined by unpaired 2-tailed Student’s *t* test; **P < 0.01.

As we observed, increased ETC activity in AA patients with BLCA could be a result of distinctive rewiring of energy metabolism in AA BLCA compared with their EA counterparts. Interestingly, metabolomics data from AA BLCA cells with NDUFB8 KD show decreased glutamine and TCA cycle metabolites, and this effect was rescued when NDUFB8 was reexpressed (Figure 4A). As stated above, metabolic rewiring occurs upon loss and gain of NDUFB8 in AA BLCA cells. We next measured the expression of glutaminase 1 (GLS1), a key enzyme in regulating free glutamine levels. Interestingly, in AA BLCA cells, KD of the NDUFB8 gene decreased the GLS1 protein expression, which was recoverable after NDUFB8 rescued in shNDUFB8-3′UTR BLCA cell lines (Figure 4B). However, this phenomenon was not observed in the EA BLCA cell lines (Figure 4B). Further analysis involving mitochondrial and cytosolic fraction separation revealed a significant reduction in GLS1 expression within mitochondrial fraction of NDUFB8 KD AA BLCA cell lines (Supplemental Figure 6A). To understand the GLS1-NDUFB8 axis associated with glutamine flux in AA BLCA, we next treated BLCA cell lines with mitochondrial complex–specific inhibitors (IACS-010759 inhibits complex I; 3-nitropropionic acid inhibits complex II; oligomycin inhibits complex V; atovaquone inhibits complex III) (29–31) and measured GLS1 expression. Cells treated with complex I–specific inhibitor IACS-010759 showed decreased GLS1 expression in AA BLCA cells, whereas other complex inhibitors do not have much effect (Figure 4C). To directly access the inhibition of complex I and glutamine metabolism, we measured endogenous metabolite levels from the orthotopic tumors, and our analysis revealed decreased glutamine levels in AA xenograft tumors (derived from UM-UC-1) upon IACS-010759 inhibition, whereas EA tumors (derived from UM-UC-3) did not show much effect (Figure 4D). With these findings, we summarize that pharmacological and genetic inhibition of one of the components of complex I (NDUFB8) was able to regulate GLS1 mediated glutamine metabolism in AA BLCA.

### Genetic and pharmacological inhibition of GLS1 impairs AA BLCA tumor growth.

To determine the role of glutamine-mediated increased ETC activity and ATP production in AA BLCA, we evaluated the expression of GLS1 in patients with BLCA and observed increased GLS1 expression in AA BLCA tissues (Supplemental Figure 6B). Next, we performed metabolic flux analysis using [U]13C-glutamine in ancestry-verified AA BLCA and EA BLCA cell lines to investigate the role of GLS1 (Figure 4E). Our analysis shows increased M+4 and M+5 citrate in AA BLCA cell lines compared with EA BLCA cell lines, indicating a preference for glutamine dependency via oxidative TCA (M+4) and reductive carboxylation (M+5) in AA BLCA cell lines (Figure 4E). As a control, we also tested the metabolic flux using [U]13C-glucose in both AA BLCA and EA BLCA cell lines (Supplemental Figure 6C). To investigate the race-specific mitochondria-mediated metabolic alterations, we analyzed the cytosolic and mitochondrial fractions of EA (UM-UC-3) and AA (UM-UC-1) BLCA cell lines and confirmed increased expression of GLS1 and NDUFB8 proteins in the mitochondrial fraction of AA BLCA compared with EA BLCA cell lines (Supplemental Figure 6D). To explore the role of the GLS1-Complex I axis in AA BLCA, we treated the 2 AA BLCA cell lines (UM-UC-1 and SCaBER) with increasing concentrations of glutamine and observed a dose-dependent increase in the expression of NDUFB8, increased OCR, and increased ATP production (Supplemental Figure 6, E and F). To confirm the role of glutamine flux in the activation of GLS1 and complex I proteins, we blocked glutamine flux using V-9302, a small molecule competitive antagonist of transmembrane glutamine flux; the addition of glutamine activates GLS1 and NDUFB8 expression, which increased OCR and ATP production. Similarly, V-9302 inhibited the increased GLS1 and NDUFB8 expression along with OCR and ATP production (Supplemental Figure 6, G and H). Based on these findings, we concluded that AA BLCA cell lines utilize more glutamine through the TCA cycle for increased ATP production through increased complex I activity–mediated mechanism.

To investigate the role of GLS1 in AA BLCA, we tested the effects of genetic and pharmacological inhibition of GLS1. We knocked down GLS1 in both AA BLCA (SCaBER) and EA (UM-UC-3) BLCA cell lines, and we confirmed the KD by Western blot analysis (Supplemental Figure 7A). As expected, GLS1 KD showed more prominent inhibition on proliferation in the AA BLCA cell line compared with the EA BLCA cell line (Supplemental Figure 7, B and C). Interestingly, GLS1 KD resulted in major inhibition in basal respiration (Supplemental Figure 7, D and F) and ATP production (Supplemental Figure 7E) in AA BLCA cells compared with EA BLCA cells (Supplemental Figure 7, G–I). In vivo orthotopic mouse tumors with GLS1 KD revealed that tumor growth is significantly reduced compared with the control group (Figure 4F). Pharmacological inhibition of GLS1 (CB-839) resulted in prominent inhibition of basal respiration and ATP production in AA BLCA cell lines compared with EA BLCA cell lines (Supplemental Figure 8, A–I). As observed in GLS1 KD cells, CB-839 treatment inhibited in vivo tumor growth in AA BLCA xenografts (Figure 4G). All these results confirm that the increased glutamine flux–mediated OXPHOS activation is a critical mechanism that supports the increased tumor aggressiveness in AA BLCA.

## Discussion

Social determinants of health confer racial disparities in BLCA that contribute to worse outcomes in AA patients (10, 11, 32), but the molecular mechanisms are not well understood. In the era of race-specific predictive biomarkers, metabolic alterations and tumor burden are playing an important role in guiding therapeutic strategies. However, there are no reliable biomarkers available to guide the selection of therapeutic regimens for BLCA based on racial differences. In addition, limited studies have attempted to investigate the intrinsic biological traits unique to AA tumors (18) that confer them a more aggressive and lethal phenotype (33–35). Along similar lines, race is poorly studied in clinical trials due to lower enrollment of AA patients (36, 37). Furthermore, the limited number of AA BLCA (~10%) patient transcriptomics data in public cohorts such as TCGA (38, 39) has made understanding the biological basis for the racial disparity in patients with BLCA particularly challenging.

Our RNA-Seq, proteomics, and metabolomics data from a cohort composed of AA BLCA and EA BLCA patient tumors shed light on AA-specific tumor biology. Our validation using RNA-Seq data from TCGA-BLCA confirmed OXPHOS as one of the top enriched pathways in AA BLCA. Utilizing state-of-art technologies, self-reported patient samples, ancestry-verified BLCA cell lines, and orthotopic preclinical models, we were able to successfully and conclusively establish that glutamine pathway–driven high mitochondrial OXPHOS activity is an intrinsic biological trait in AA BLCA tumors that contributes to their aggressiveness. From literature over recent years, it is evident that cancer cells with high tumorigenic potential are more reliant upon OXPHOS activity, which provides a potential opportunity for utilizing metabolic inhibitors as cancer therapy (26, 40–43). Our study reveals that the high rate of mitochondrial respiration observed in AA BLCA tumors is driven at least in part by the elevation of complex I activity. This highlights the importance of mitochondrial OXPHOS in driving tumor progression and enabling therapy resistance, as demonstrated in breast cancer (44, 45), melanoma (46), pancreatic cancer (47), gastric cancer (48), and leukemia (49).

Pharmacological inhibition of OXPHOS activity using the complex I inhibitor (IACS-010759) was able to regress tumor growth in AA BLCA. Through extensive analysis of patient tumors, preclinical xenograft, and ancestry-verified BLCA cell lines, we found that enhanced OXPHOS activity through increased complex I activity is prevalent in AA BLCA. Our study focused on NDUFB8, one of the components of complex I, and its role in AA BLCA progression. Given that complex I comprises numerous subunits (27), it is plausible that other components may also exert effects worthy of investigation in future studies.

Depletion of NDUFB8 (a complex I accessory subunit) reduced the OXPHOS-dependent ATP pool and abrogated the tumor growth in AA BLCA tumors, highlighting complex I role in linking race-specific mitochondrial activity and tumor progression in AA patients with BLCA.

Glutamine is one of the nonessential amino acids in the human body that contributes to a majority of the biosynthetic pathways in various cancers (50). Cancer cells undergo a metabolic switch from glucose to glutamine pathway to meet their energy demand to synthesize biomolecules required for tumor growth (51). In this context, there are few studies demonstrated that breast cancer in AA patients is dependent on glutamine metabolism in disease progression (52). In addition, it was shown that glutamine metabolism is linked to patient survival in breast cancer (53). In this study, we have identified the glutamine pathway as the major carbon source that supports the increased complex I and OXPHOS activity in AA BLCA. Interestingly, a complex I–driven feedback activation of GLS1 also contributes to the maintenance of the glutamine-OXPHOS axis in AA BLCA to maintain the higher energy demands through TCA cycle that feeds NADH for ETC activity supporting tumor growth (54, 55).

In conclusion, our study shows that the enhanced GLS1 and NDUFB8 expression maintains an upregulated glutamine-OXPHOS axis in AA BLCA. Increased NDUFB8 expression and complex I activity in AA BLCA tumors with enhanced glutamine uptake and elevated glutaminolysis induce tumor progression. This metabolic rewiring–driven tumor progression provides a unique therapeutic vulnerability for AA patients with BLCA.

The limitation of the current study is the availability of AA BLCA cell lines. In this study, we have used UM-UC-1 (derived from urothelial carcinoma) and SCaBER (derived from squamous cell carcinoma) cell lines, which are of AA lineage and are ancestry verified. Since tumor mutations can alter many intrinsic pathways, studies in a larger number of AA and EA BLCA cell lines and models with different BLCA subtypes may be necessary. While our analysis across different cohorts of AA BLCA and EA BLCA patient tumor and benign/normal samples provide unique mechanistical insights, further studies involving larger sample sizes are necessary to validate these findings due to the relatively small sample sizes in the current study. Further analysis of correlations with genomic mutations may be important to identify the subtype of AA BLCA that are most dependent on our proposed mechanisms of metabolic rewiring.

## Methods

### Sex as a biological variable

The current study included both male and female human BLCA and normal/benign samples. For mouse studies, our assessment was on male mice for tumor growth and therapeutic experiments. Sex was not considered as a biological variable for the mouse experiments, since all experiments were performed in male mice.

### Clinical samples

For this study, human BLCA and benign/normal tissues were collected from Baylor College of Medicine (BCM), Augusta University (AU), University of Maryland Baltimore (UMB), Cooperative Human Tissue Network (CHTN), and University of Texas Southwestern Medical Center (UTSW) and were purchased from National Disease Research Interchange (NDRI) obtained with prior consent with approved IRB protocols and stored at –140°C until further analysis. Samples were annotated based on self-reported race, sex, smoking status, and pathological stage of BLCA (Supplemental Tables 1 and 2). All the samples are collected in a deidentified manner. In all cohorts, race was self-reported, and Caucasian/EA/White was labeled as EA BLCA and AA/Black was labeled as AA BLCA.

### RNA-Seq and library preparation

#### NEBNext ultra II directional RNA-Seq sample preparation with rRNA depletion protocol.

Total RNA samples are normalized to 100 ng each, based on Qubit quantitation. Samples underwent ribosomal RNA depletion using a RNAse H-based method and subsequently fragmented and primed with random hexamers to produce first-strand cDNA. RNA templates were removed during second-strand synthesis and replaced with cDNA strands containing dUTP. The resulting directionally identifiable ds-cDNA was then purified using Beckman Coulter AMPure XP beads. Libraries were created from the cDNA by attaching an adenosine to the 3′ end and ligating adapters to the ends. The unique barcodes were incorporated into the ligated products during amplification. Agilent 2100 Bioanalyzer and nanodrop spectrophotometer were used to assess the fragment size and quantify the libraries, respectively. A quantitative PCR (qPCR) assay was performed on the libraries to determine the concentration of adapter ligated fragments using the Applied Biosystems ViiA7 Quantitative PCR instrument and KAPA Library Quant Kit (KK4824).

#### Illumina sequencing.

In total, 150 pM of equimolarly pooled libraries were loaded onto a NovaSeq S4 flowcell (Illumina, 20028312) following the XP Workflow protocol and amplified by exclusion amplification onto a nanowell-designed, patterned flowcell using the Illumina NovaSeq 6000 sequencing instrument at Genomic and RNA Profiling (GARP) Core at BCM. A paired-end 150 bp cycle run was used to sequence the flowcell on a NovaSeq 6000 Sequencing System. An average of 50 million read pairs per sample was sequenced. FastQ file generation was executed using Illumina’s cloud-based informatics platform, BaseSpace Sequencing Hub.

#### Analysis.

For transcriptomic quantification and annotation, the reads were quality trimmed using Trim Galore and FastQC. They were then aligned to the hg19 genome using HISAT2. Gene expression was quantified using feature counts against the gencode.v24 gene reference. DEGs were detected using DEseq2, with significance achieved at *P* < 0.05. During our RNA-Seq analysis, we used less stringent criteria for *P* values in all comparisons due to a limited sample number; this is a limitation of the study. As a note, we used the RNA-Seq method for prioritization, to identify the potential pathway that was validated further by multiple experimental data including proteomics, metabolomics, and molecular biology assays.

#### Pathway analysis.

DEGs from RNA-Seq data were used for pathway enrichment analysis using python 2.7. Hypergeometric enrichment analysis was conducted to identify key pathways that enriched in Hallmark pathways collections. A nominal *P* < 0.05 used as threshold for determining their significance, and *P* value was calculated via the hypergeometric enrichment method. The number of genes in the pathways versus significance of the pathway was plotted using RStudio and represented.

### Proteomics analysis

#### Nano–LC-MS/MS analysis.

All the nano–LC-MS/MS analysis was performed on a Q Exactive Plus mass spectrometer (Thermo Fisher Scientific) equipped with an EASY-Spray nano-ESI source and Vanquish Neo UHPLC System (Thermo Fisher Scientific) using a trap-and-elute injection mode. Trypsin-digested peptides (300 ng) were injected onto an Acclaim PepMap 100 trap column (75 micron ID × 2 cm, Thermo Fisher Scientific) and eluted onto an Acclaim PepMap Neo UHPLC analytical column (75 micron ID × 50 cm, Thermo Fisher Scientific), and the peptides were separated using a 5%–29% gradient of solvent B (acetonitrile [ACN], 0.1% formic acid [FA]) over 92 minutes, 29%–57% of solvent B over 10 minutes, 57%–99% of solvent B over 5 minutes, a hold of solvent 99% B for 5 minutes, and finally a column wash at 99% of solvent B for 6 minutes. Solvent A consisted of water and 0.1% FA, and solvent B was 0.1% FA in 90% ACN. Flow rates were 300 nL/min using a Vanquish Neo UHPLC System (Thermo Fisher Scientific). Data-dependent scanning was performed by the Xcalibur v 4.7.69.37 software using a survey scan at 70,000 resolving power scanning mass/charge (*m/z*) 300–1,600 at an automatic gain control (AGC) target of 1 × 10^6^ and a maximum injection time (IT) of 65 msec, followed by higher-energy collisional dissociation (HCD) tandem MS (MS/MS) at 27 normalized collision energy, of the 11 most intense ions at a resolving power of 17,500, an isolation width of 1.5 *m/z*, an AGC of 5 × 10^4^, and a maximum IT of 65 msec. Dynamic exclusion was set to place any selected *m/z* on an exclusion list for 30 seconds after a single MS/MS. Ions of charge state +1, 6, 7, 8, >8, unassigned, and isotopes were excluded from MS/MS. Acquired raw data files were further processed for analysis at BCM.

#### Protein identification and data analysis.

Obtained spectra were searched against the target-decoy Human RefSeq database (release 2020) in Proteome Discoverer 2.1 interface (PD 2.1, Thermo Fisher Scientific) with the Mascot algorithm (Mascot 2.4, Matrix Science). Dynamic modifications of the acetylation of N-terminus and oxidation of methionine were allowed. The precursor mass tolerance was confined within 20 ppm with fragment mass tolerance of 0.5 Da, and a maximum of 2 missed cleavages was allowed. Protein inference and quantitation were conducted using gpGrouper (v1.0.040) incorporating a shared peptide intensity-based absolute quantification (iBAQ) area distribution (56). This software employed a universal peptide grouping logic to ensure precise allocation and MS1 based quantification across multiple gene products. Quantification of gene-protein products (GPs) was achieved through label-free, iBAQ. The iBAQ-based fraction of total values (iFOT) was calculated by dividing the iBAQ for each gene product by the total species iBAQ. Then, each iFOT was divided by the corresponding gene product iFOT for the internal reference and log_2_ transformed. The resulting protein values underwent median normalization and log transformation to facilitate downstream analyses. For statistical assessment, the missing value was replaced with half of minimum of recovered proteome. Differentially expressed proteins were determined with significance of *P* < 0.05. For MS-based proteomics, we used a *P* value due to limited sample numbers.

#### Pathway analysis.

In this analysis, significant differentially expressed proteins from proteomics data were used for the pathway analysis. Protein encoding genes were used for pathway enrichment analysis with python 2.7. As described above, hypergeometric enrichment analysis was conducted to identify key pathways that enriched in Hallmark pathways. A nominal *P* < 0.05 used as threshold for determining their significance.

### Metabolomics analysis

#### Sample preparation.

Metabolites were extracted from BLCA tissues, cell lines, and mouse liver. Pooled samples of mouse liver were used for quality control. About 25 mg of tissue and cell pellets (3 million cells) were used to extract the metabolites. Cells were frozen and thawed for 3 consecutive cycles of 30 seconds each in liquid nitrogen and water. Then, both tissue and cell pellets were homogenized in 1:4 (750 μL) ice cold water/methanol mixture (v/v) containing an equimolar mixture of internal standards. This was followed by the sequential application of ice-cold organic and aqueous solvents as chloroform/water (3:1 v/v). The organic and aqueous layers were collected, dried, and resuspended with 500 μL of 1:1 methanol/water (v/v). The extract was deproteinized using a 3 kDa Amicon-Ultra filter (Millipore Corporation) and the filtrate was dried under vacuum (Genevac) (57)**.**

#### LC-MS.

The dried samples were resuspended in methanol/water (1:1 v/v) and analyzed using ultra–high performance LC (UPLC) coupled to MS/MS (Agilent 1290 infinity series UPLC system and Agilent 6495 mass spectrometer; Agilent Technologies) (13, 58).

For targeted metabolic profiling, an X-bridge amide column (3.5 μm, 4.6 × 100 mm; Waters) was applied in electrospray ionization (ESI) positive mode as described earlier (57). Mobile phase A and B were 0.1% FA in water and ACN, respectively. Gradient flow was as follows: 0–3 minutes 85% B; 3–12 minutes 85%–30 % B, 12–15 minutes 30%–2 % B, and 16 minutes 95% B, followed by reequilibration until the end of the gradient at 23 minutes to the initial starting condition of 85% B. Flow rate of the solvents used for the analysis was 0.3 mL/min. The injection volume was 5 μL.

For steady state TCA, glycolysis intermediates were measured using (3 μm,150 × 2 mm, Luna NH2, 100 pore size [A^0^], Phenomenex) with 5 mM ammonium acetate in water pH 9.9 as buffer (A) and 100% ACN as buffer (B) in ESI negative mode as described earlier (59, 60). The binary gradient utilized is as follows: from 0 to 20 minutes, the composition of solvent B was maintained at 80%, with a flow rate of 0.2 mL/min. Between 20 and 20.10 minutes, there was a linear decrease from 80% to 2% of solvent B. Subsequently, from 20.10 to 25 minutes, the solvent composition remained at 2% of solvent B with 0.3 mL/min flow rate. From 25 to 30 minutes, there was a return to 80% of solvent B with a flow rate of 0.35 mL/min. This gradient was maintained from 30 to 38 minutes at a 0.4 mL/min flow rate. Finally, the system was reequilibrated to the initial condition of 80% of solvent B at a flow rate of 0.2 mL/min.

#### LC-MS data review and analysis.

The acquired data were analyzed, and review and integration of each peak, along with each of isotopomers peak for metabolic flux, was done using Agilent Mass Hunter Quantitative Analysis software (Agilent Technologies).

#### Statistical analysis for metabolomics.

The data was log_2_ transformed followed by internal standard normalization on a per-sample, per-method basis. For every metabolite in the normalized data set, 2-tailed *t* tests were conducted to compare expression levels between AA BLCA and EA BLCA. Differential metabolites were identified by adjusting the *P* values for multiple testing at an FDR threshold of < 0.25. The R statistical software system generated a hierarchical cluster of the differentially expressed metabolites (https://www.r-project.org/).

#### Pathway analysis.

Differentially expressed metabolites were mapped to associated genes using HMDB database (61). Using mapped genes, we conducted the pathway analysis with python 2.7. As described above Hypergeometric Enrichment analysis was conducted to identify key hallmark pathways. A nominal *P* < 0.05 used as threshold for determining their significance.

### Cell lines used for this study

UM-UC-3, T24, J82, and SCaBER (HTB-3) cell lines were purchased from ATCC, and UM-UC-1 and UM-UC-5 cell lines were purchased from Sigma-Aldrich. As per ATCC, the SCaBER cell line was derived from AA BLCA, and T24 and J82 cell lines were derived from EA BLCA. Furthermore, genomic DNA was isolated from all BLCA cell lines and genotyped to determine the genetic ancestry performed at Morehouse School of Medicine, as previously described (62–64). The identity of human cell lines was verified by short tandem repeat (STR) fingerprinting at least every 6 months.

### Cell culture and treatments

All BLCA cells were grown in stable MEM (0.4344 g/L stable glutamine, 1 g/L glucose, and no sodium pyruvate) supplemented with 10% FBS (Sigma-Aldrich) and 1× penicillin-streptomycin (Sigma-Aldrich) at 37°C under 5% CO_2_. All cell lines were confirmed negative for mycoplasma prior to the study using the MycoAlert Mycoplasma Detection Kit (Lonza). For glutamine supplementation experiments represented in Supplemental Figure 6, E and F, cells were cultured in glutamine-free media (no glutamine, 1 g/L glucose, and no sodium pyruvate) for 2 hours and supplemented with 0 mM, 1 mM, 2.5 mM, and 5 mM glutamine up to 48 hours. For glutamine supplementation experiments represented in Supplemental Figure 6, G and H, cells were cultured in glutamine-free media (no glutamine, 1 g/L glucose, and no sodium pyruvate) for 2 hours and supplemented with 1 mM glutamine along with 2.5 μM V-9302 up to 48 hours. All the drugs used to treat the cell lines are dissolved in DMSO vehicle or otherwise specified. In total, 1 μM of CB-839 and 2.5 μM of V-9302 was used for in vitro assays. For mitochondrial complex inhibitor treatment experiments represented in Figure 4C, BLCA cell lines were treated with 10 μM of IACS-010759, 3-nitropropionic acid, oligomycin, and atovaquone separately in stable MEM (0.4344 g/L stable glutamine, 1 g/L glucose, and no sodium pyruvate). After 48 hours of treatment, cells were collected and processed for further analysis. Manufacturer for all products used for the study are provided in Supplemental Table 13.

#### Lentivirus packaging and stable knockdown or overexpression cell line generation.

GLS1 (targeting CDS region) and NDUFB8 (targeting CDS and 3′UTR region) KD cells were generated using GIPZ vectors (Supplemental Table 12). For virus packaging, Lipofectamine 2000 transfection reagent (Thermo Fisher Scientific) was used to cotransfect the control or target shRNA constructs with lentiviral packaging constructs psPAX2 and pMD2.G (Addgene) into HEK293T cells. After 48 hours, the virus-containing medium was collected and then filtered through a 0.45 μm cellulose acetate filter. BLCA cells were seeded in a 6-well plate and cultured for 24 hours until 60%–70% of the cells were confluent. These cells were incubated with lentivirus for 48 hours, stable KD cells were selected with puromycin (1 μg/mL). KD of specific genes in the stable cell lines was detected by Western blot analysis. For overexpression of NDUFB8 in the KD (shNDUFB8-3′UTR) background cells (rescue), the full-length NDUFB8 (pHAGE-EF1a-NDUFB8-IRES-Luc-pgk-blast) or control (pHAGE-EF1a-Luc-pgk-blast) plasmids (generated by the ACE&3M core at BCM) were used to generate lentivirus and infect BLCA cells as described above. Stable overexpression cells were selected with blasticidin (1 μg/mL) (23).

#### 13C metabolic flux using all carbon labeled [U]13C-glutamine and [U]13C-glucose.

An equal number of AA BLCA cells (SCaBER, UM-UC-1) and EA BLCA cells (UM-UC-3, J82) was seeded in 10 cm dishes in stable MEM (0.4344 g/L stable glutamine, 1 g/L glucose, and no sodium pyruvate) supplemented with 10% FBS and 1× penicillin-streptomycin at 37°C under 5% CO_2_ overnight; the next day, all the cells were starved with glutamine-free (no glutamine, 1 g/L glucose, and no sodium pyruvate)/glucose-free (584 mg/L glutamine, no glucose, and no sodium pyruvate) media with 10% dialyzed FBS for 2 hours and then incubated with media containing 4 mM [U]13C-glutamine and/or 12 mM [U]13C-glucose in glutamine-free and/or glucose-free media along with dialyzed FBS. Six hours of incubation were used for glycolysis and TCA metabolite measurement. After incubation, the medium was removed, and the cells were washed 3 times with cold PBS. Equal numbers of cells were collected, snap frozen in liquid nitrogen, and stored at –80°C until metabolite extraction. [U]13C-glucose– or [U]13C-glutamine–labeled cells were freeze thawed in liquid nitrogen and were then homogenized in methanol/water (1:1) using needle sonication. The samples were centrifuged at 10 minutes at 4°C for 2,400*g*–2,500*g*. Next, the samples were filtered using a 3K Amicon filter to remove the proteins and lipids, dried under a speed vacuum, and reconstituted with methanol/water (1:1). Similar to targeted metabolomics, glycolysis flux and TCA flux were measured as described in earlier publications (59, 65).

#### Metabolic flux analysis.

For metabolic flux and the incorporation measurements, the peak areas for the labeled and unlabeled metabolites were log transformed. *P* value are calculated by 2-tailed *t* test using log transformed values. Following the log transformation, we used *Z* score transformation of the data represented as a heatmap for all the labeled and unlabeled metabolites.

### Western blot analysis

Western blotting was performed as previously described (66). BLCA tissues and cell lines were collected and lysed with RIPA buffer containing protease and phosphatase inhibitor cocktail. Protein concentrations were measured using BCA assay. All the patient tissue samples were obtained from tumor bank in deidentified manner; lysate was aliquoted depending on sample amount and stored in –80**°**C until the analysis. Equal amounts of protein were separated by SDS-PAGE and transferred to PVDF filter membrane. Membranes were blocked with 5% BSA and immunoblotted with primary antibodies followed by HRP-conjugated secondary antibodies. Protein expression was visualized using Bio-Rad ChemiDoc XRS chemiluminescence detection and imaging system. All the antibodies used in this study were listed in Supplemental Table 13. All the cell line Western blots were run in at least 3 biological replicates.

### Reverse phase protein array (RPPA)

Protein extracts were prepared from BLCA tissues (~20 mg) following established protocols (67, 68). The extraction utilized modified tissue protein extraction reagent (TPER) from Invitrogen. These extracts were denatured in SDS sample buffer at a concentration of 0.5 mg/mL. Subsequently, the protein extracts were printed on to nitrocellulose membrane-coated slides (Grace Bio-Labs) by Quanterix 2470 Arrayer (Quanterix Billerica). Each extract was printed in triplicate. As previously described, antibody labeling was performed (1 antibody per slide, simultaneously for multiple slides) by automated slide stained Autolink 48 (Agilent/Dako), and for normalization purposes, a negative control slide without primary antibody and total protein slides were stained with Sypro Ruby (23, 67, 68). The antibody signal was spot-wise subtracted from the corresponding negative control signal and normalized against the total protein signal. A median of 3 replicates from the normalized signal intensity of GLS1 protein expression was analyzed using GraphPad Prism V10.2.3. Significance was determined using Student’s *t* test.

### ETC analysis

Flash-frozen BLCA tissues (~50 mg) were lysed using homogenate buffer and then centrifuged at 600*g* at 4°C to clear the lysates. BLCA cells (5 × 10^6^) were lysed in cell lysis buffer and then sonicated using Qsonica sonicator at 4°C. The protein concentration was determined by Bradford assay to allow for normalization. ETC enzyme activity was analyzed in triplicates using Tecan Infinite M200, as previously described (69, 70). ETC complexes and citrate synthase activity measurements were normalized to the protein concentration, and kinetics of the activity were calculated from the slope of the reaction. Following this, ETC complex values were normalized with citrate synthase. GraphPad Prism V10.2.3 was used to plot, and significance was calculated using *t* test.

### CellTiter-Glo proliferation assay

Cell proliferation was assessed using the Promega CellTiter-Glo assay kit as per manufacturer instructions. Cells were plated in 96-well white plates of 5,000 cells per well and cultured in MEM with 10% FBS. About 90 μL of media from wells were removed, and the cells were incubated with CellTiter-Glo reagent for 10 minutes. Luminescence reading is directly proportional to cell number and measured with microplate reader Tecan Infinite M200. GraphPad Prism V10.2.3 was used to plot the values as arbitrary units (AU), and significance was calculated using *t* test.

### Clonogenic assay

BLCA cells (500 cells/well) were cultured in 6-well plates. After incubating at 37°C for 2 weeks, the colonies were fixed and incubated with crystal violet for 15 minutes. The plates were scanned using Bio-Rad ChemiDoc XRS imaging system. The number of colonies was manually counted using ImageJ software (NIH) and analyzed using GraphPad Prism V10.2.3.

### OCR and ECAR activity by Seahorse

Measurement of OCR and ECAR measurements in ancestry-verified BLCA cell lines was performed using an Agilent Seahorse XF Cell Mito Stress Test Kit with XFe96 analyzer (Agilent Technologies). The measurement of OCR and ECAR values serves as direct quantification of mitochondrial respiration and glycolysis. Both AA BLCA and EA BLCA cells (20,000 cells/well) were seeded in an Agilent Seahorse 96-well XF cell culture microplate and were maintained for 24 hours in the CO_2_ incubator. Calibrant solution was placed prior to the analysis, and the sensor cartridge was hydrated in a non-CO_2_ 37°C incubator overnight. The basic energy metabolism profiles of the AA BLCA and EA BLCA cell lines, as well as key parameters of mitochondrial function, were assessed by sequential addition of mitochondrial perturbing agents with final concentrations — 2.5 μM of oligomycin (A), 1 μM of Carbonyl cyanide-4 (trifluoromethoxy) phenylhydrazone (FCCP) (B), and 0.5 μM rotenone/antimycin A (C). A, B, and C represent injection of the specific drugs at a specific time. Data were normalized with cell number with counting or CellTiter-Glo in Wave2.6.0. OCR (pmol/min); ECAR (mpH/min) and ATP production were analyzed with Wave2.6.0 and plotted by GraphPad Prism V10.2.3.

### IHC analysis

Formalin-fixed, paraffin-embedded (FFPE) BLCA and benign/normal tissue sections (5 μm) were used to generate tissue microarray (TMA). TMA slides were stained with anti–mouse NDUFB8 antibody (Supplemental Table 13) with 1:400 dilution along with controls and were counterstained with Hematoxylin at UTHealth Histopathology core, performed as per their standard operational procedures. Furthermore, IHC staining was analyzed by board-certified anatomic pathologist for extent and intensity. The immunoreactive score (IRS) was calculated according to previously published method (23) and analyzed using GraphPad Prism V10.2.3.

### Isolation of cytosolic and mitochondrial fraction from BLCA cell lines

In total, 5 × 10^6^ BLCA cells were used to isolate mitochondrial and cytosolic fraction using a mitochondrial isolation kit as described by the manufacturer instructions (Sigma-Aldrich, MITOISO2). Once the cytosolic and mitochondria fraction was extracted, 15 μg protein was used to run a Western blot to measure the target proteins by chemiluminescence. Tom20 and β-actin were used as loading controls for mitochondrial and cytosolic fraction, respectively.

### In vivo studies

NSG mice (4–6 weeks of age, male mice) were obtained from The Jackson laboratory. Bladder orthotopic xenograft experimental details were described in an earlier publication (23). Briefly, for orthotopic surgery, mice were anesthetized by isoflurane, and buprenorphine SR (1 mg/kg) and meloxicam (5 mg/kg) were administered 1 hour prior to the surgery and meloxicam (5 mg/kg) for postoperation up to 5 days. The surgical site was clipped; then, any additional hair was removed with depilatory cream (Nair). The surgical site was then prepped aseptically with 3 alternating preps of betadine and alcohol. Local analgesia at the incisional line was achieved by subdermal infiltration with a 50/50 mix of lidocaine 2% and bupivacaine 0.5% diluted 1:20 in sterile saline. Sterile techniques were used by using autoclaved instruments, sterile surgical gloves, sterile drapes, and aseptic technique. A bead sterilizer was used between mice to flash sterilize the instrument tips. In preparation of the orthotopic injection of luciferase-tagged BLCA cells into the bladder wall, a 26G needle syringe was used. Surgery started once the animal was in a state of surgical plane anesthesia (monitored with the toe pinch maneuver). With a pair of forceps, an area of skin ~7 mm superior to the external genitalia was lifted, and a vertical incision was made (approximately 1–1.5 cm in length) on the linea alba to open the lower abdominal cavity. The urinary bladder was carefully retracted with sterile forceps and held with sterile cotton swab. BLCA cell suspensions (50 μL contain 25 μL of cell suspension and 25 μL of matrigel) were injected into the wall of the bladder using a 26G syringe with 200,000 cells per mice. The bladder was put back in place, and the muscle layer was sutured at least 3 times with #5-0 Vicryl suture. The skin incision was closed with tissue adhesive and clips. Postoperative mice were held in a clean cage with sterile bedding, and the cage was kept over a heated pad to keep the mice warm after surgery until they recovered from isoflurane anesthesia and adopted sternal recumbency and normal movement. For confirmation of orthotopic injections, mice were imaged after 7 days of surgery by removing the wound clips. Mice were injected with luciferin (15 mg/mL) via i.p. route; we waited 5 minutes before mice were imaged under IVIS (71). Tumor progression was monitored by IVIS (Spectral Instruments, Living Image 3).

For s.c. experiment, 100 μL of SCaBER (1 × 10^6^ cells/mice; 50:50 cells/matrigel) cells were implanted into the right side of the nude male mice, which were obtained from The Jackson laboratory. Once tumors reached 100 mm^3^, mice were randomized and started the IACS-010759 (5 mg/kg) treatment orally.

#### In vivo treatments.

IACS-010759 was dissolved in DMSO and prepared in saline with 0.1% methyl acetate solution. In total, 5 mg/kg concentration was used to treat the mice by oral gavage daily. Calithera Biosciences Inc. supplied formulated CB-839 along with vehicle control, under approved material transfer agreement (MTA) between BCM and Calithera Biosciences. The drug was prepared at 20 mg/mL in 25% (w/v) hydroxypropyl-b-cyclodextrin in 10 mmol/L citrate (pH 2). Animals were dosed with 200 mg/kg body weight, continuously twice a day (7 days a week) via oral gavage. Body weight of the mice in all the experiments were measured weekly once.

### Reagents used for this study

All the antibodies, compounds, and other reagents used for this study are listed in Supplemental Table 13.

### Statistics

Statistical significance was determined for 2-sided analysis at a significance level of *P* < 0.05. GraphPad Prism v10.2.3 was utilized for graph analysis, while R software and GraphPad Prism v10.2.3 was employed for heatmap generation. Data are shown as mean ± SD or SEM as specified.

### Study approval

All human samples were acquired under the H-35808 protocol approved by BCM IRB. All animal experiments were performed according to the guidelines and requirements of BCM IACUC under protocol no. AN-7324 in accordance with IACUC guidelines. For the study, all the experiments were performed according to the protocol approved by the Institutional Biological & Chemical (IBC) Safety Committee at BCM, under the protocol no. D-499.

### Data availability

All the raw data are available in Supporting Data Values and supplemental material. Raw and processed RNA Sequence data from samples are uploaded at GeoHub (GSE261944; https://www.ncbi.nlm.nih.gov/geo/query/acc.cgi?acc=GSE261944). The MS data for proteome profiling have been submitted via the MASSIVE repository (MSV000095559) to the ProteomeXchange Consortium (http://proteomecentral.proteomexchange.org) with the data set identifier PXD054731. Raw and processed metabolomics data from samples are available at Metabolomics workbench (ID: ST002464).

## Author contributions

KRKR contributed project conception; performed in vitro and in vivo experiments; analyzed data; takes full responsibility for the finished work and/or the conduct of the study, had access to the entire data set, and conducted manuscript writing. DWBP performed the transcriptomics, proteomics, metabolomics, and pathway analysis. JHP performed the in vitro and ex vivo ETC complex activity assay. VP performed steady state metabolomics analysis for cell lines and patient tissues as well as metabolic flux analysis in cell lines. CSA performed experiments for response to reviewer comments. AHMK performed steady state metabolomics analysis for cell lines and patient tissues as well as metabolic flux analysis in cell lines. JX performed lentivirus preparation for KD and overexpression of genes. DK performed RNA-Seq. SH performed RPPA data generation and analysis. SYJ performed proteomics data analysis. LSE provided BLCA and benign/normal tissues as well as clinical information for CHTN cohort. JRJ and RAK contributed by performing ancestry genotyping analysis to verify ancestry of BLCA cell lines. LYB provided the pathological scoring for TMA analysis and helped to calculate the IRS score. KP performed proteomics analysis using MS from BLCA and benign/normal tissues. MMS provided the clinical samples used in this study and contributed editorial and clinical input. JG provided the editorial and clinical input. ALG, RJB, and MKT provided clinical samples, clinical input, and edited the manuscript. YL provided clinical samples, intellectual input, clinical input, and edited the manuscript. CJC provided data analysis for TCGA Pan-Cancer Atlas used in response to reviewers. SPL provided clinical samples, intellectual input, and clinical input and edited the manuscript. AS provided intellectual input, helped on procuring normal bladder tissues, and edited the manuscript. BAK provided intellectual input and manuscript writing, had access to the entire data set, and controlled the decision to publish. NP provided project conception and intellectual input, had access to the entire data set, provided supervision and manuscript writing, and controlled the decision to publish.

## Supplementary Material

Supplemental data

Unedited blot and gel images

Supplemental table 1

Supplemental table 10

Supplemental table 11

Supplemental table 12

Supplemental table 13

Supplemental table 2

Supplemental table 3

Supplemental table 4

Supplemental table 5

Supplemental table 6

Supplemental table 7

Supplemental table 8

Supplemental table 9

Supporting data values

## Version 1 09/10/2024

Electronic publication
