# Supplementary material for: Mitochondrial reprogramming by activating OXPHOS via glutamine metabolism in African American patients with bladder cancer
Source: JCI Insight. 2024 Sep 10;9(17):e172336. doi: 10.1172/jci.insight.172336 (PMC11385078; doi:10.1172/jci.insight.172336)
Supplement: Supplemental data [file jciinsight-9-172336-s269.pdf]

Supplementary Figures

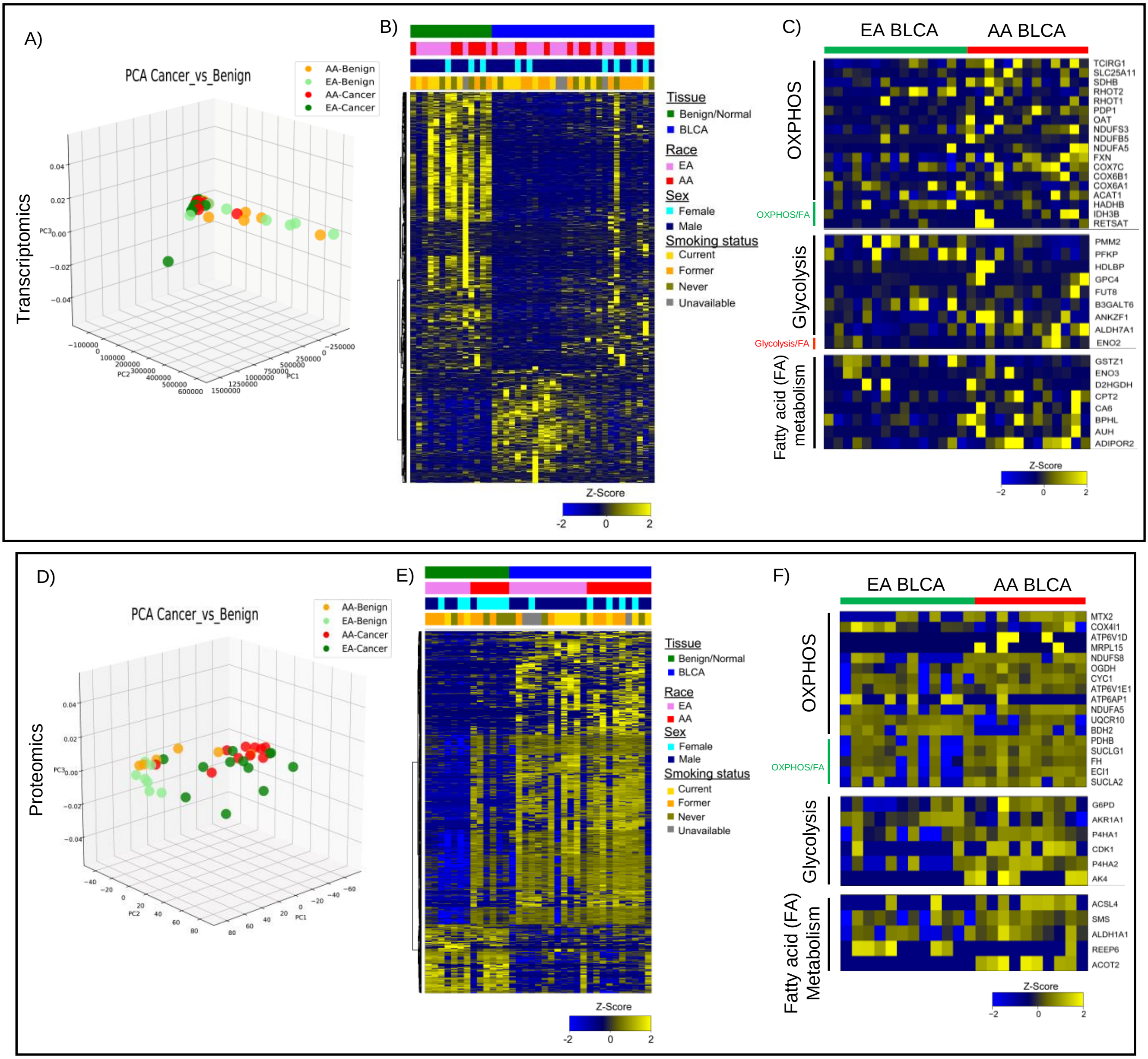
A)
PCA Cancer_vs_Benign
AA-Benign
EA-Benign
AA-Cancer
EA-Cancer
Transcriptomics
B)
Tissue
Benign/Normal
BLCA
Race
EA
AA
Sex
Female
Male
Smoking status
Current
Former
Never
Unavailable
Z-Score
-2
0
2
C)
EA BLCA
AA BLCA
OXPHOS
OXPHOS/FA
Glycolysis
Glycolysis/FA
Fatty acid (FA) metabolism
TCIRG1
SLC25A11
SDHB
RHOT2
RHOT1
PDP1
OAT
NDUFS3
NDUFB5
NDUFA5
FXN
COX7C
COX6B1
COX6A1
ACAT1
HADHB
IDH3B
RETSAT
PMM2
PFKP
HDLBP
GPC4
FUT8
B3GALT6
ANKZF1
ALDH7A1
ENO2
GSTZ1
ENO3
D2HGDH
CPT2
CA6
BPHL
AUH
ADIPOR2
Z-Score
-2
0
2
D)
PCA Cancer_vs_Benign
AA-Benign
EA-Benign
AA-Cancer
EA-Cancer
Proteomics
E)
Tissue
Benign/Normal
BLCA
Race
EA
AA
Sex
Female
Male
Smoking status
Current
Former
Never
Unavailable
Z-Score
-2
0
2
F)
EA BLCA
AA BLCA
OXPHOS
OXPHOS/FA
Glycolysis
Fatty acid (FA) Metabolism
MTX2
COX4I1
ATP6V1D
MRPL15
NDUFS8
OGDH
CYC1
ATP6V1E1
ATP6AP1
NDUFA5
UQCR10
BDH2
PDHB
SUCLG1
FH
ECI1
SUCLA2
G6PD
AKR1A1
P4HA1
CDK1
P4HA2
AK4
ACSL4
SMS
ALDH1A1
REEP6
ACOT2
Z-Score
-2
0
2

Supplementary Figure 1. A) Principal component analysis (PCA) plot using RNA sequencing profile from AA benign/normal (n=6), EA benign/normal (n=8), AA BLCA (n=13) and EA BLCA (n=15). B) Heatmap for RNA sequencing data showing altered genes in BLCA tissues (n=28) compared to benign/normal tissues (n=14) (FDR <0.25). Samples are collected from Augusta University (AU), University of Texas Southwestern (UTSW), University of Maryland, Baltimore (UMB) and Cooperative Human Tissue Network (CHTN). C) Heatmap represent the oxidative phosphorylation (OXPHOS), glycolysis and fatty acid (FA) metabolic pathway genes from RNA sequencing (Same RNA sequencing data was also used for Figure 1A and Supplementary Figure 1A and 1B) in AA BLCA (n=13) and EA BLCA (n=15) (P<0.05). Some of the genes are overlapped in OXPHOS/FA and Glycolysis/FA are highlighted in the heatmap. D) PCA plot using proteomics profile from AA benign/normal (n=6), EA benign/normal (n=7), AA BLCA (n=10) and EA BLCA (n=12). E) Heatmap for proteomics data showing altered proteins in BLCA tissues (n=22) compared to benign/normal tissues (n=13) (P<0.05). Samples are collected from Augusta University (AU), University of Texas Southwestern (UTSW), University of Maryland, Baltimore (UMB) and Baylor College of Medicine (BCM) or purchased from National Disease Research Interchange (NDRI). F) Heatmap represent the OXPHOS, glycolysis and fatty acid (FA) metabolic pathway proteins from proteomics in AA BLCA (n=10) and EA BLCA (n=12) (P<0.05). Data derived from proteomics used for Figure 1C and Supplementary Figure 1D and 1E. Some of the proteins are overlapped in OXPHOS/FA are highlighted in the heatmap.

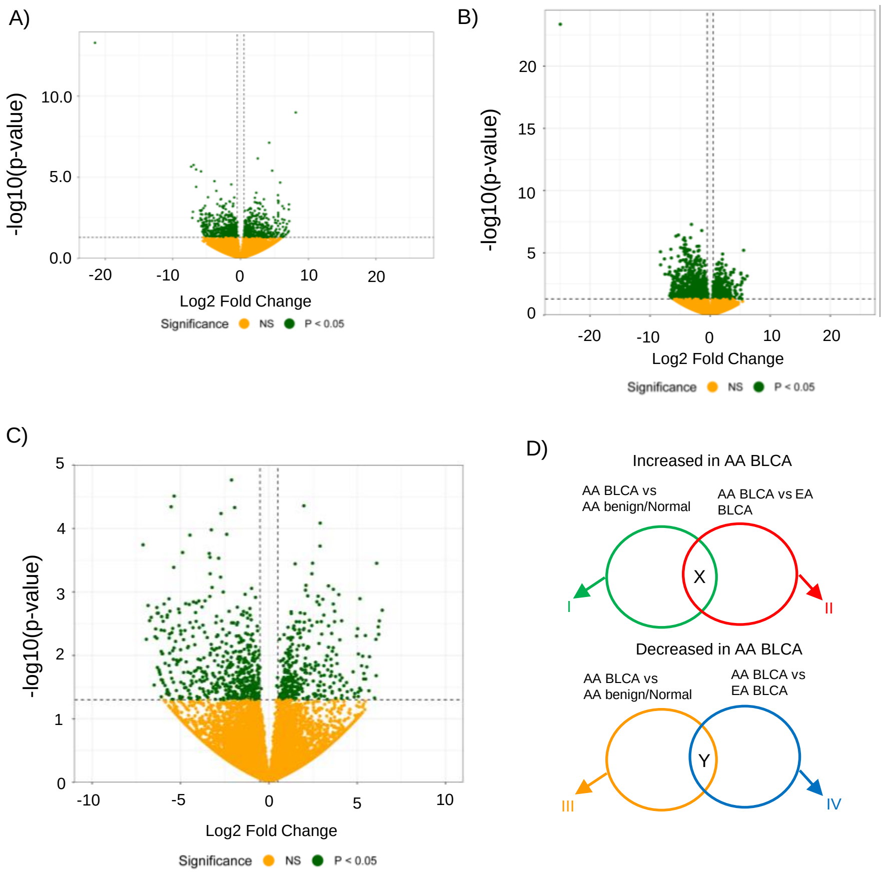
A)
-log10(p-value)
10.0
5.0
0.0
-20
-10
0
10
20
Log2 Fold Change
Significance NS P < 0.05
B)
-log10(p-value)
20
15
10
5
0
-20
-10
0
10
20
Log2 Fold Change
Significance NS P < 0.05
C)
-log10(p-value)
5
4
3
2
1
0
-10
-5
0
5
10
Log2 Fold Change
Significance NS P < 0.05
D)
Increased in AA BLCA
AA BLCA vs AA benign/Normal
AA BLCA vs EA BLCA
X
I
II
Decreased in AA BLCA
AA BLCA vs AA benign/Normal
AA BLCA vs EA BLCA
Y
III
IV

Supplementary Figure 2: A) Volcano plot represents differentially expressed genes between AA BLCA (n=13) vs AA Benign/normal (n=6) derived from RNA seq data (P<0.05 denoted as green dots; non-significant (NS) denoted as yellow dots). B) Volcano plot represents differentially expressed genes between EA BLCA (n=15) vs EA Benign/normal (n=8) derived from RNA seq data (P<0.05 denoted as green dots; non-significant (NS) denoted as yellow dots). C) Volcano plot represents differentially expressed genes between AA benign/normal (n=6) vs EA Benign/normal (n=8) derived from RNA seq data (P<0.05 denoted as green dots; non-significant (NS) denoted as yellow dots). D) Venn diagram represent the common transcriptional gene signature by comparing between AA BLCA vs AA benign/normal (I=444; III=548; total:992) derived from Supplementary Figure 2A and AA BLCA vs EA BLCA (II=817; IV=616) derived from Figure 1A. Top panel show increased in AA BLCA bottom panel decreased in AA BLCA. Commonly shared increased in AA BLCA (X=38), decreased in AA BLCA (Y=29) are highlighted (bold text) in Supplementary Table 7 and 10. All DEGs in comparison between AA BLCA vs AA benign/normal are listed in Supplementary Table 7 and all DEGs in comparison between AA BLCA vs EA BLCA are listed in Supplementary Table 10.

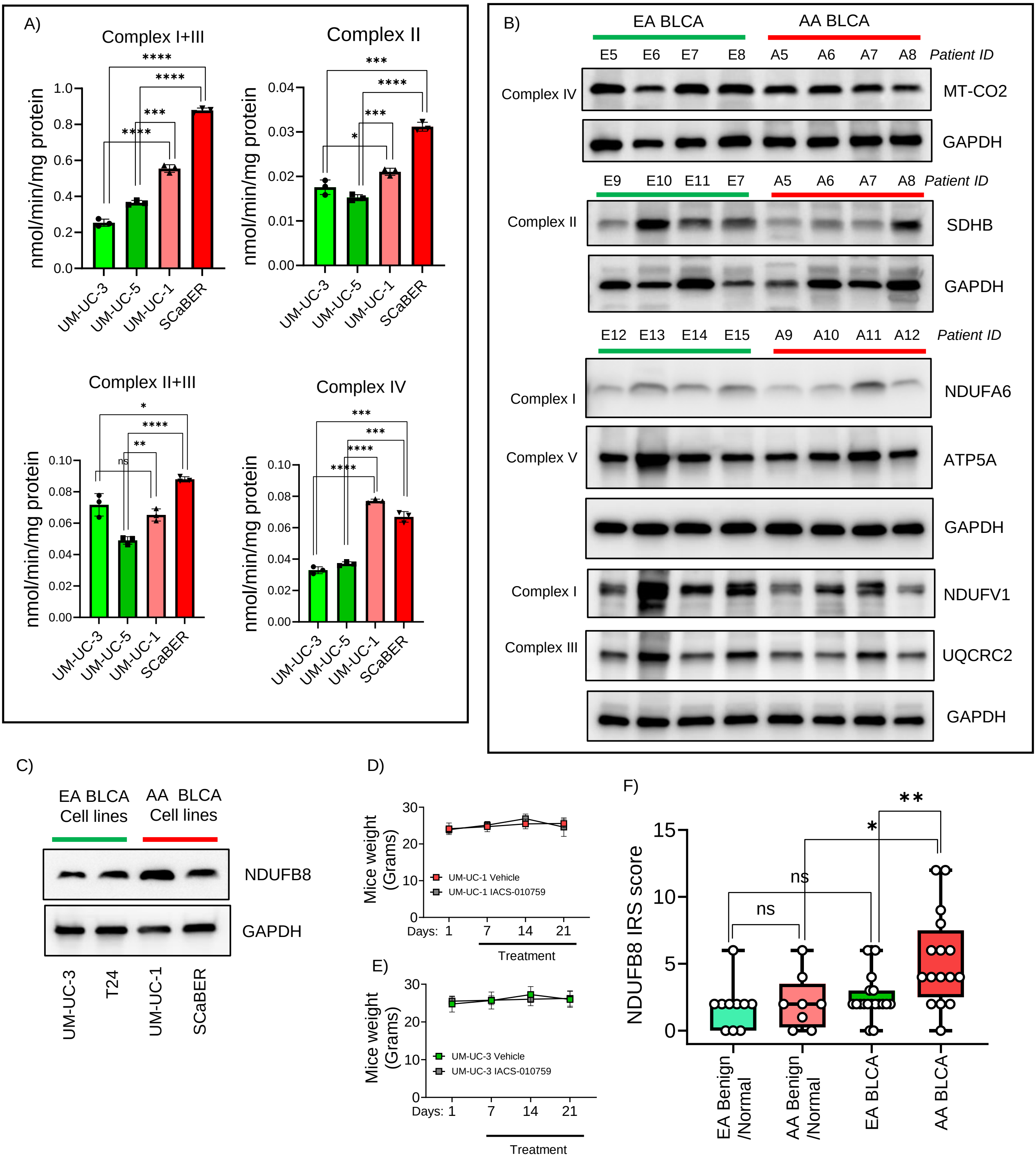

A)
Complex I+III
Complex II
Complex II+III
Complex IV
nmol/min/mg protein
UM-UC-3
UM-UC-5
UM-UC-1
SCaBER
ns
B)
EA BLCA
AA BLCA
E5 E6 E7 E8 A5 A6 A7 A8 Patient ID
Complex IV
MT-CO2
GAPDH
E9 E10 E11 E7 A5 A6 A7 A8 Patient ID
Complex II
SDHB
E12 E13 E14 E15 A9 A10 A11 A12 Patient ID
Complex I
NDUFA6
Complex V
ATP5A
NDUFV1
Complex III
UQCRC2
C)
EA BLCA Cell lines
AA BLCA Cell lines
NDUFB8
UM-UC-3
T24
D)
Mice weight (Grams)
UM-UC-1 Vehicle
UM-UC-1 IACS-010759
Days: 1 7 14 21
Treatment
E)
UM-UC-3 Vehicle
UM-UC-3 IACS-010759
F)
NDUFB8 IRS score
EA Benign /Normal
AA Benign /Normal
EA BLCA
AA BLCA

Supplementary Figure 3: A) Scatter plots representing the differential mitochondrial complex activities in AA BLCA [UM-UC-1 (n=3), SCaBER(n=3)] cell lines compare to EA BLCA [UM-UC-3 (n=3), UM-UC-5 (n=3)] cell lines. OD of complex activity was measured using specific substrates which was measured by colorimetric assay. Data was normalized with Citrate synthase (CS) (****=P<0.0001; ***=P<0.001; **=P<0.01; *=P<0.05; ns= non significance). B) Protein expression of MT-CO2, SDHB, NDUFA6, ATP5A, NDUFV1, UQCRC2 in EA BLCA and AA BLCA patient tissues. We have used the same set of the patient samples (E7, A5-A8) for MT-CO2 and SDHB. Additionally, E12-E15, A9-A12 patient samples were used to probing NDUFA6, ATP5A, NDUFV1 and UQCRC2 (refer to unedited blot in Supplementary Material). GAPDH was used as loading control. C) Western blot analysis shows protein expression of NDUFB8 in EA BLCA and AA BLCA cell lines. D-E) Line plots represent the UM-UC-1(D) and UM-UC-3 (E) harboring mice weight from vehicle and IACS-010759 treatment arms derived from Figure 2D (UM-UC-1) and 2E (UM-UC-3). F) Immunohistochemistry analysis of NDUFB8 in EA benign/normal (n=10), AA benign/normal (n=8), EA BLCA (n=19) and AA BLCA (n=16) from tissue micro array. IRS score was used for the analysis. Significance was determined by unpaired two-tailed student t-test.

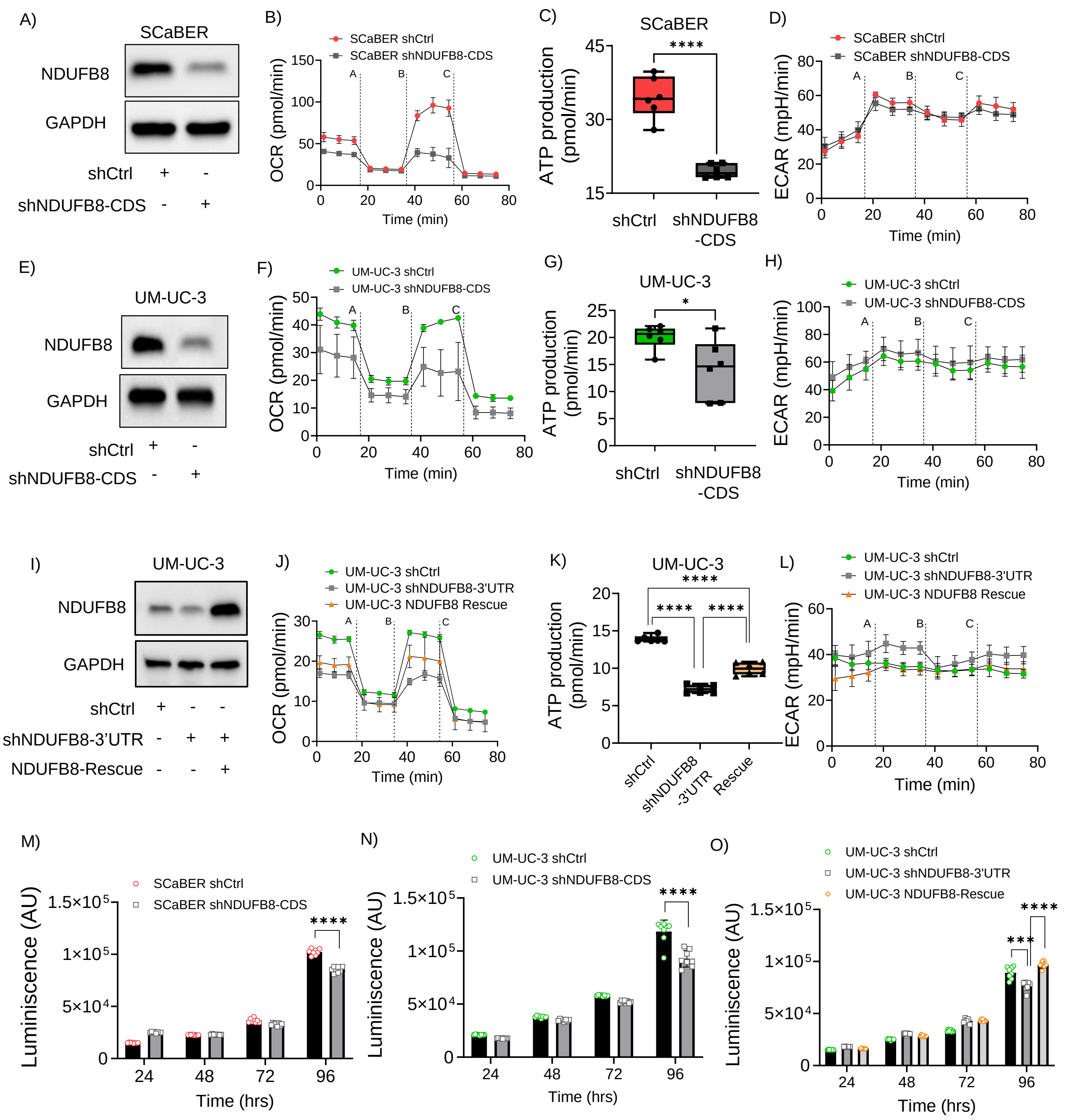
A)
SCaBER
NDUFB8
GAPDH
shCtrl + -
shNDUFB8-CDS - +
B)
SCaBER shCtrl
SCaBER shNDUFB8-CDS
OCR (pmol/min)
Time (min)
C)
SCaBER
ATP production (pmol/min)
****
shCtrl
shNDUFB8 -CDS
D)
SCaBER shCtrl
SCaBER shNDUFB8-CDS
ECAR (mpH/min)
Time (min)
E)
UM-UC-3
NDUFB8
GAPDH
shCtrl + -
shNDUFB8-CDS - +
F)
UM-UC-3 shCtrl
UM-UC-3 shNDUFB8-CDS
OCR (pmol/min)
Time (min)
G)
UM-UC-3
ATP production (pmol/min)
*
shCtrl
shNDUFB8 -CDS
H)
UM-UC-3 shCtrl
UM-UC-3 shNDUFB8-CDS
ECAR (mpH/min)
Time (min)
I)
UM-UC-3
NDUFB8
GAPDH
shCtrl + - -
shNDUFB8-3'UTR - + +
NDUFB8-Rescue - - +
J)
UM-UC-3 shCtrl
UM-UC-3 shNDUFB8-3'UTR
UM-UC-3 NDUFB8 Rescue
OCR (pmol/min)
Time (min)
K)
UM-UC-3
ATP production (pmol/min)
****
**** ****
shCtrl
shNDUFB8 -3'UTR
Rescue
L)
UM-UC-3 shCtrl
UM-UC-3 shNDUFB8-3'UTR
UM-UC-3 NDUFB8 Rescue
ECAR (mpH/min)
Time (min)
M)
SCaBER shCtrl
SCaBER shNDUFB8-CDS
Luminiscence (AU)
****
Time (hrs)
N)
UM-UC-3 shCtrl
UM-UC-3 shNDUFB8-CDS
Luminiscence (AU)
****
Time (hrs)
O)
UM-UC-3 shCtrl
UM-UC-3 shNDUFB8-3'UTR
UM-UC-3 NDUFB8-Rescue
Luminiscence (AU)
***
****
Time (hrs)

Supplementary Figure 4: A) Immunoblot analysis shows the confirmation of NDUFB8 KD in AA BLCA cell line (SCaBER) compared to non-targeting control. B) KD of NDUFB8 (n=6) in SCaBER significantly reduces basal respiration compared to shControl (n=6) measured by seahorse assay (A=oligomycin; B=FCCP; C=Rotenone/Antimycin A, data was normalized with cell number by counting). C) same as in B, but for ATP production (****=P<0.0001) and D) for extra cellular acidification rate (ECAR). E) Immunoblot analysis shows the confirmation of NDUFB8 KD in EA BLCA cell line (UM-UC-3) compared to non-targeting control. F) KD of NDUFB8 (n=6) in UM-UC-3 reduces basal respiration compared to shControl (n=6) measured by seahorse assay (A=oligomycin; B=FCCP; C=Rotenone/Antimycin A, data was normalized with cell number by counting). G) same as in F, but for ATP production (*=P<0.05) and H) for ECAR. I) Immunoblot analysis shows the confirmation of NDUFB8 KD and rescue in UM-UC-3 compared to non-targeting control. Rescue of NDUFB8 was generated from full length NDUFB8 overexpression in KD (shNDUFB8-3'UTR) cells. J) KD of NDUFB8 (n=6) in UM-UC-3 significantly reduces basal respiration compared to shControl (n=6) and rescued upon NDUFB8 re-expression (n=6) measured by seahorse assay (A=oligomycin; B=FCCP; C=Rotenone/Antimycin A, data was normalized with cell number by counting). K) same as in J, but for ATP production (****=P<0.0001) and L) for ECAR. M-O) CellTiter-Glo® proliferation assay shows significantly reduced in NDUFB8 KD (n=8) compared to shControl (n=8) and rescued upon NDUFB8 re-expression (n=8) in SCaBER and UM-UC-3 cell line at 96 hours (***=P<0.001, ****=P<0.0001). Significance was determined by unpaired two-tailed student t-test.

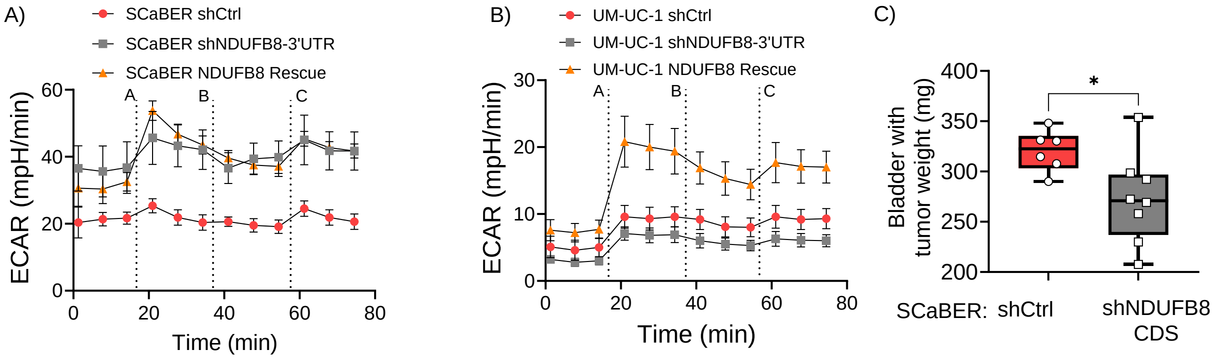
A)
SCaBER shCtrl
SCaBER shNDUFB8-3'UTR
SCaBER NDUFB8 Rescue
ECAR (mpH/min)
0
20
40
60
A
B
C
Time (min)
0
20
40
60
80
B)
UM-UC-1 shCtrl
UM-UC-1 shNDUFB8-3'UTR
UM-UC-1 NDUFB8 Rescue
ECAR (mpH/min)
0
10
20
30
A
B
C
Time (min)
0
20
40
60
80
C)
Bladder with tumor weight (mg)
200
250
300
350
400
*
SCaBER:
shCtrl
shNDUFB8 CDS

Supplementary Figure 5: A-B) Extracellular acidification rate (ECAR) plots for SCaBER (A) and UM-UC-1 (B) cell lines. (OCR experimental data presented in Figure 3C and 3D). C) Box & Whiskers plot representing the weight of orthotopic bladders harboring tumors (End point: day 28) from SCaBER shCtrl (n=6) and shNDUFB8-CDS (n=8) (*=P<0.05). Significance was determined by unpaired two-tailed student t-test.

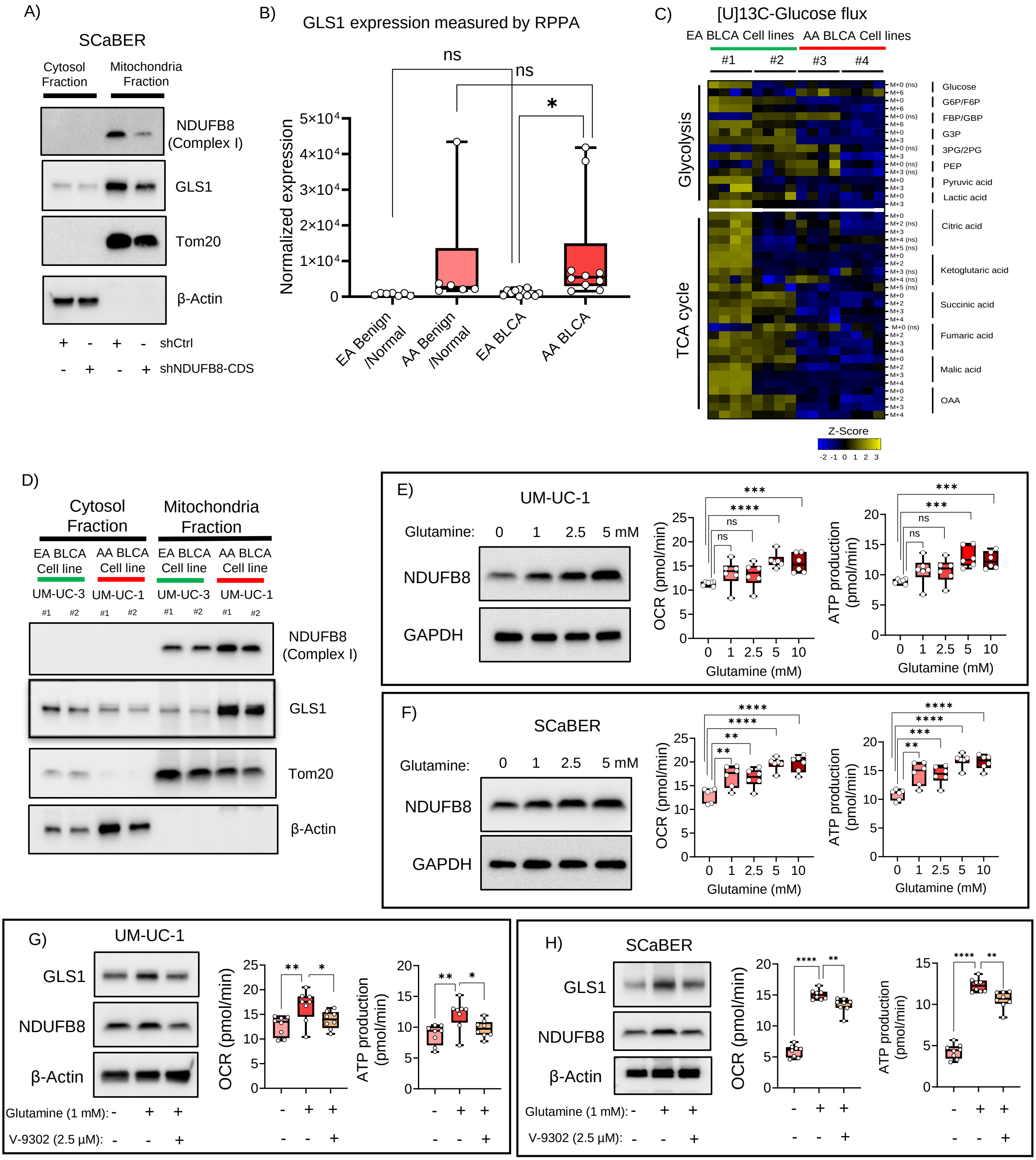

A)
SCaBER
Cytosol Fraction
Mitochondria Fraction
NDUFB8 (Complex I)
GLS1
Tom20
β-Actin
+ - + - shCtrl
- + - + shNDUFB8-CDS
B)
GLS1 expression measured by RPPA
ns
ns
*
Normalized expression
EA Benign /Normal
AA Benign /Normal
EA BLCA
AA BLCA
C)
[U]13C-Glucose flux
EA BLCA Cell lines
AA BLCA Cell lines
#1 #2 #3 #4
Glycolysis
TCA cycle
Glucose
G6P/F6P
FBP/GBP
G3P
3PG/2PG
PEP
Pyruvic acid
Lactic acid
Citric acid
Ketoglutaric acid
Succinic acid
Fumaric acid
Malic acid
OAA
Z-Score
D)
Cytosol Fraction
Mitochondria Fraction
EA BLCA Cell line
AA BLCA Cell line
UM-UC-3
UM-UC-1
NDUFB8 (Complex I)
GLS1
Tom20
β-Actin
E)
UM-UC-1
Glutamine: 0 1 2.5 5 mM
NDUFB8
GAPDH
OCR (pmol/min)
ATP production (pmol/min)
Glutamine (mM)
F)
SCaBER
Glutamine: 0 1 2.5 5 mM
NDUFB8
GAPDH
OCR (pmol/min)
ATP production (pmol/min)
Glutamine (mM)
G)
UM-UC-1
GLS1
NDUFB8
β-Actin
Glutamine (1 mM): - + +
V-9302 (2.5 µM): - - +
OCR (pmol/min)
ATP production (pmol/min)
H)
SCaBER
GLS1
NDUFB8
β-Actin
Glutamine (1 mM): - + +
V-9302 (2.5 µM): - - +
OCR (pmol/min)
ATP production (pmol/min)

Supplementary Figure 6: A) Immunoblot analysis for GLS1 and NDUFB8 expression from cytosolic and mitochondrial fraction from SCaBER shCtrl and shNDUFB8-CDS cell lines. Tom20 and β-actin were used as loading controls for mitochondrial and cytosolic fractions, respectively. B) Protein expression of GLS1 from RPPA in EA benign/normal (n=7), AA benign/normal (n=6), EA BLCA (n=12) and AA BLCA (n=10). Normalized protein expression was used for the analysis and plotted as box & whiskers plot (Significance was determined by unpaired two-tailed student t-test; *=P<0.05; ns= non-significance). C) Heatmap representing glycolysis and TCA flux (6 hours) using [U]-13C glucose in EA BLCA [#1=UM-UC-3 (n=4), #2=J82 (n=4)] and AA BLCA [#3=SCaBER (n=4), #4=UM-UC-1 (n=4)] cell lines. Peak areas were converted to log2 and followed by z-score transformation. Significance was determined based on log transformed data using student t-test between AA BLCA cell lines and EA BLCA cell lines. Yellow represents increased and blue represent decreased levels from z score values. Non-significant metabolites were represented as ns. D) Immunoblot analysis for GLS1 and NDUFB8 expression from cytosolic and mitochondrial fraction in AA BLCA (UM-UC-1; n=2) and EA BLCA (UM-UC-3; n=2) cell lines. Tom20 and β-actin were used as loading controls for mitochondrial and cytosolic fractions, respectively. E-F) NDUFB8 expression by immunoblot analysis and OCR (middle panel), ATP production (right panel) measured by seahorse assay using UM-UC-1 (E) and SCaBER (F) cells treated with different concentrations of glutamine (Significance was determined by unpaired two-tailed student t-test; ****=P<0.0001; ***=P<0.001; **=P<0.01; ns= non significance. Data was normalized with cell number measured by CellTiter-Glo® and n=6 replicates were used for each condition for UM-UC-1 and SCaBER). G-H) GLS1 and NDUFB8 expression measured by immunoblot analysis and OCR (middle panel), ATP production (right panel) measured by seahorse assay using UM-UC-1 (G) and SCaBER (H) cells treated with glutamine and V-9302 (Significance was determined by unpaired two-tailed student t-test; ****=P<0.0001; **=P<0.01; *=P<0.05; ns= non significance. Data was normalized with cell number measured by CellTiter-Glo® and n=8 replicates were used for each condition for UM-UC-1 and SCaBER).

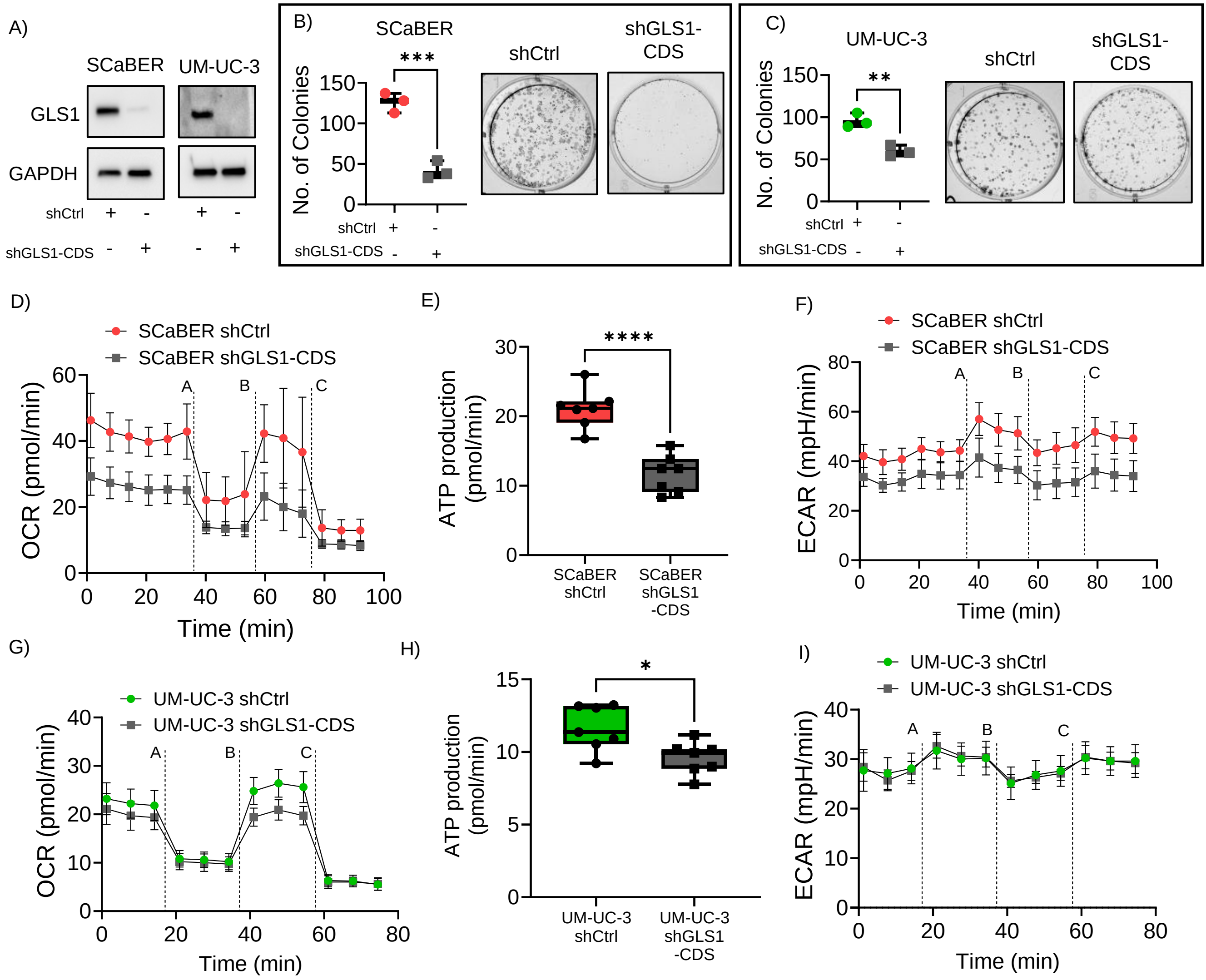
A)
SCaBER
UM-UC-3
GLS1
GAPDH
shCtrl + - + -
shGLS1-CDS - + - +
B)
SCaBER
***
No. of Colonies
shCtrl
shGLS1-CDS
C)
UM-UC-3
**
No. of Colonies
shCtrl
shGLS1-CDS
D)
SCaBER shCtrl
SCaBER shGLS1-CDS
OCR (pmol/min)
Time (min)
E)
****
ATP production (pmol/min)
SCaBER shCtrl
SCaBER shGLS1 -CDS
F)
SCaBER shCtrl
SCaBER shGLS1-CDS
ECAR (mpH/min)
Time (min)
G)
UM-UC-3 shCtrl
UM-UC-3 shGLS1-CDS
OCR (pmol/min)
Time (min)
H)
*
ATP production (pmol/min)
UM-UC-3 shCtrl
UM-UC-3 shGLS1 -CDS
I)
UM-UC-3 shCtrl
UM-UC-3 shGLS1-CDS
ECAR (mpH/min)
Time (min)

Supplementary Figure 7: A) Immunoblot analysis showing GLS1 KD in AA BLCA (SCaBER) and EA BLCA (UM-UC-3) cell lines. B) Box & whiskers plots representing number of colonies in SCaBER cell line with shCtrl (n=3) and shGLS1-CDS (n=3) (***P<0.001). Representative images of clonogenic assay with SCaBER shCtrl and shGLS1-CDS. C) Box & whiskers plots representing number of colonies in UM-UC-3 cell line with shCtrl (n=3) and shGLS1-CDS (n=3) (**P<0.01). Representative images of clonogenic assay with UM-UC-3 shCtrl and shGLS1-CDS. D-F) Basal respiration (D) (A=oligomycin; B=FCCP; C=Rotenone/Antimycin A, data was normalized with cell number by counting), ATP production (E) is significantly reduced in SCaBER cell line with GLS1 KD (n=7) compared to shControl (n=7) (****=P<0.0001) along with ECAR plot (F) measured by seahorse assay, respectively. G-I) Basal respiration (G) (A=oligomycin; B=FCCP; C=Rotenone/Antimycin A, data was normalized with cell number by counting), ATP production (H) using seahorse assay in UM-UC-3 cell lines with GLS1 KD (n=7) and shControl (n=7) (*=P<0.05) along with ECAR plot (I), respectively. Significance was determined by unpaired two-tailed student t-test.

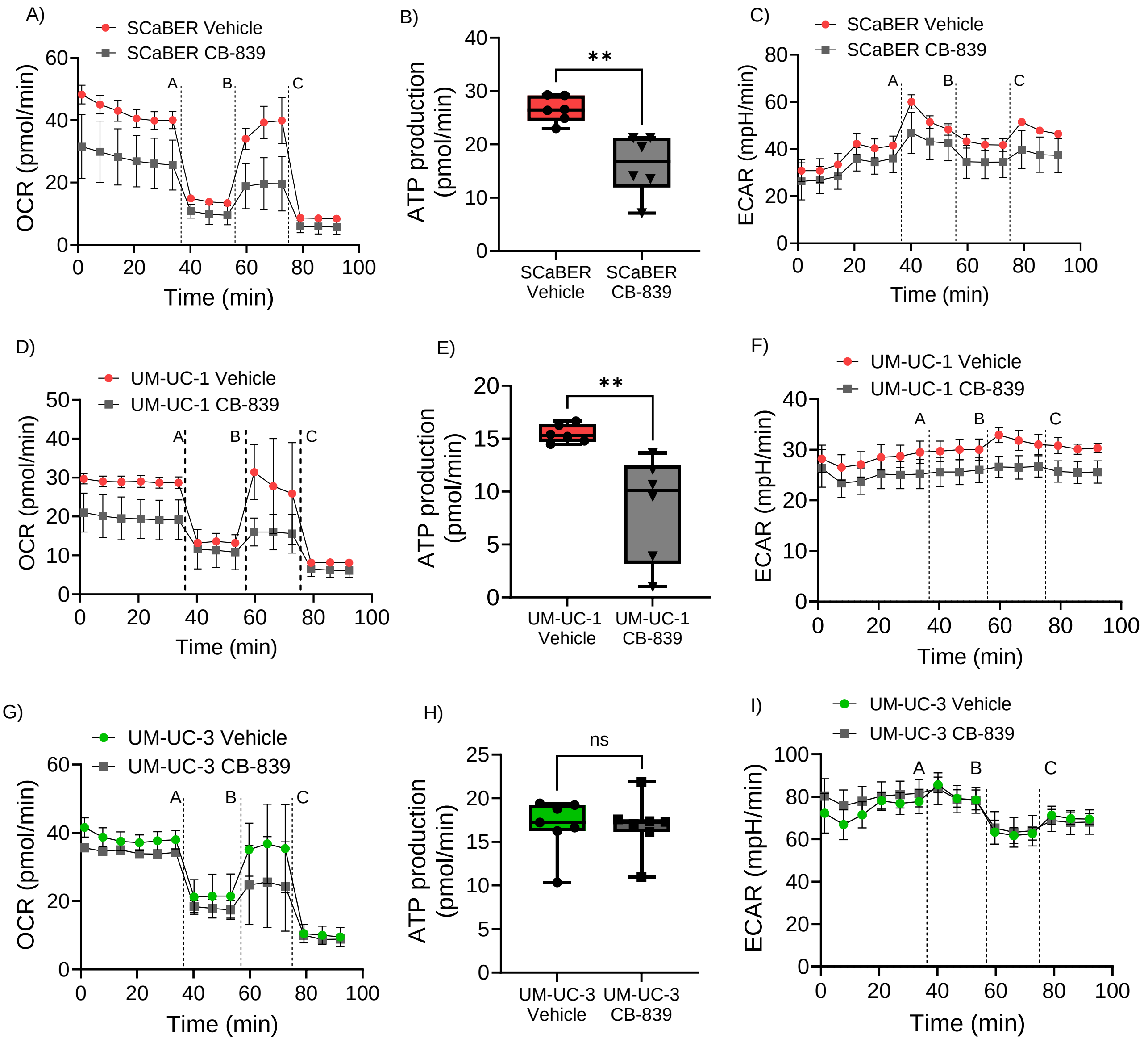

A)
SCaBER Vehicle
SCaBER CB-839
OCR (pmol/min)
Time (min)
B)
ATP production (pmol/min)
**
SCaBER Vehicle
SCaBER CB-839
C)
SCaBER Vehicle
SCaBER CB-839
ECAR (mpH/min)
Time (min)
D)
UM-UC-1 Vehicle
UM-UC-1 CB-839
OCR (pmol/min)
Time (min)
E)
ATP production (pmol/min)
**
UM-UC-1 Vehicle
UM-UC-1 CB-839
F)
UM-UC-1 Vehicle
UM-UC-1 CB-839
ECAR (mpH/min)
Time (min)
G)
UM-UC-3 Vehicle
UM-UC-3 CB-839
OCR (pmol/min)
Time (min)
H)
ATP production (pmol/min)
ns
UM-UC-3 Vehicle
UM-UC-3 CB-839
I)
UM-UC-3 Vehicle
UM-UC-3 CB-839
ECAR (mpH/min)
Time (min)

Supplementary Figure 8: A-C) Basal respiration (A) (A=oligomycin; B=FCCP; C=Rotenone/Antimycin A, data was normalized with cell number by counting), ATP production (B) shows significant effect in AA BLCA (SCaBER) cell line treated with CB-839 (n=6) compared with vehicle (n=6) (**=P<0.01) along with ECAR plot (C) measured by seahorse assay, respectively. D-F) Basal respiration (D) (A=oligomycin; B=FCCP; C=Rotenone/Antimycin A, data was normalized with cell number by counting), ATP production (E) shows significant effect in AA BLCA (UM-UC-1) cell line treated with CB-839 (n=6) compared with vehicle (n=6) (**=P<0.01) along with ECAR plot (F) measured by seahorse assay, respectively. G-I) Basal respiration (G) (A=oligomycin; B=FCCP; C=Rotenone/Antimycin A, data was normalized with cell number by counting), ATP production (H) shows in EA BLCA (UM-UC-3) cell line treated with CB-839 (n=7) compared with vehicle (n=7) (ns= non significance) along with ECAR plot (I) measured by seahorse assay, respectively. Significance was determined by unpaired two-tailed student t-test.

Supporting Material

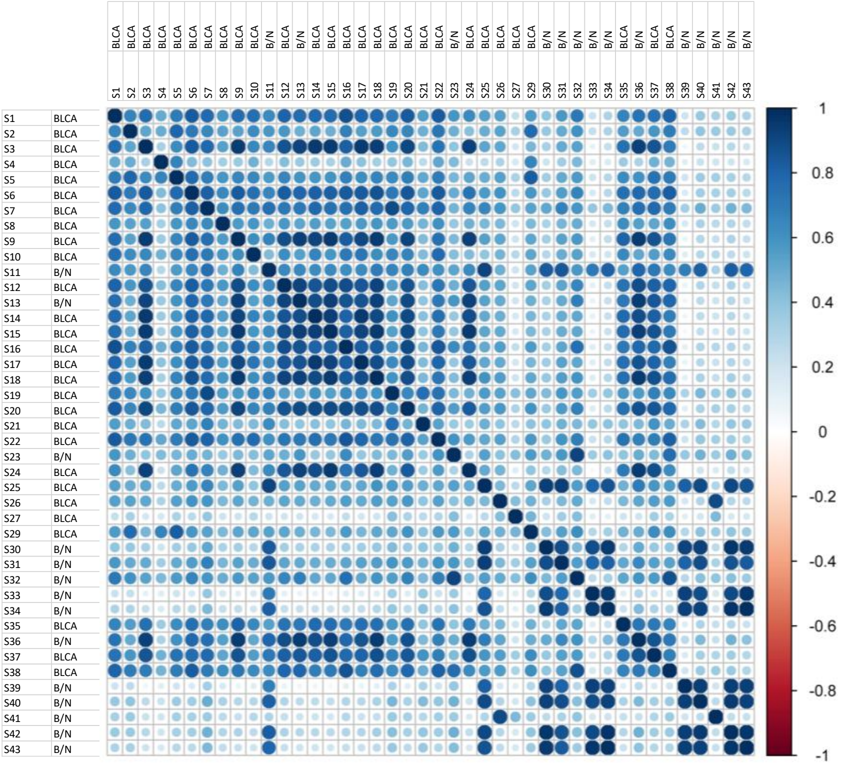

Supporting Material A: Sample to sample Correlation matrix of RNA sequencing profiling from benign/normal (B/N), bladder cancer (BLCA) samples used for this study

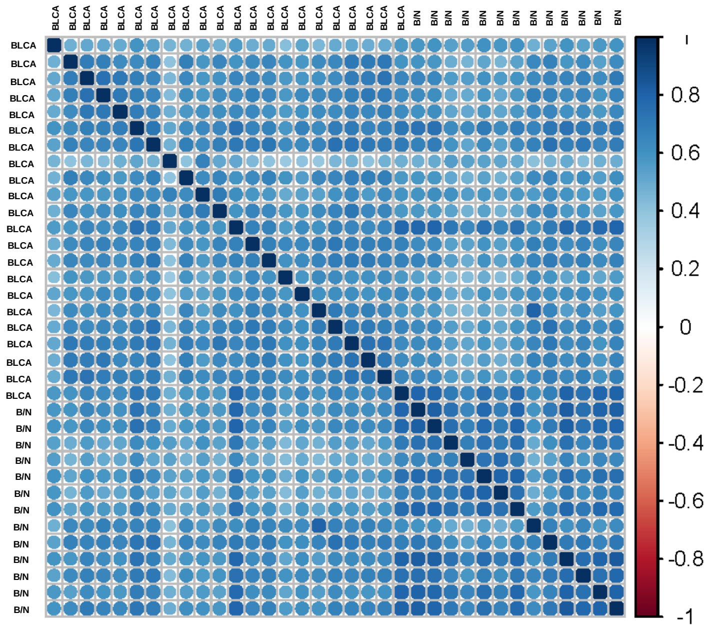

Supporting Material B: Sample to sample Correlation matrix of proteomics profiling from benign/normal (B/N), bladder cancer (BLCA) samples used for this study
