# Supplementary material for: Mitochondrial reprogramming by activating OXPHOS via glutamine metabolism in African American patients with bladder cancer
Source: JCI Insight. 2024 Sep 10;9(17):e172336. doi: 10.1172/jci.insight.172336 (PMC11385078; doi:10.1172/jci.insight.172336)
Supplement: Unedited blot and gel images [file jciinsight-9-172336-s270.pdf]

Mitochondrial reprogramming by activating OXPHOS via glutamine metabolism in African American patients with bladder cancer.

Full unedited blots and raw images associated with Figures and Supplementary figures.

Unedited blots associated with Figure 2A

Overlaid with ladder

Raw image

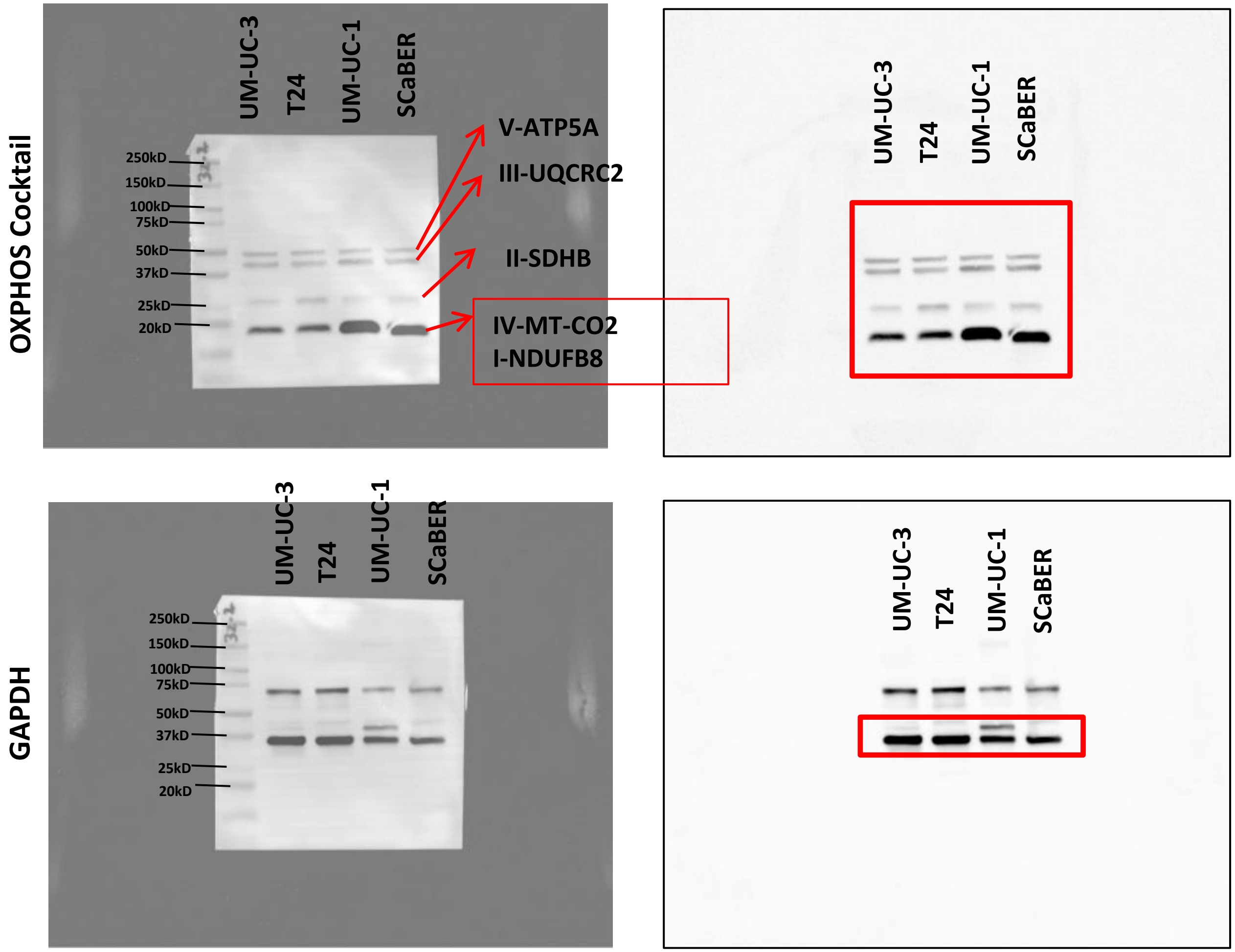

Area used for assembled the figure

OXPHOS : 1 in 1000, Abcam
GAPDH: 1 in 5000, CST

Blot no: 32-2

Unedited blots associated with Figure 2G

Overlaid with ladder | **Original**

NDUFB8

GAPDH

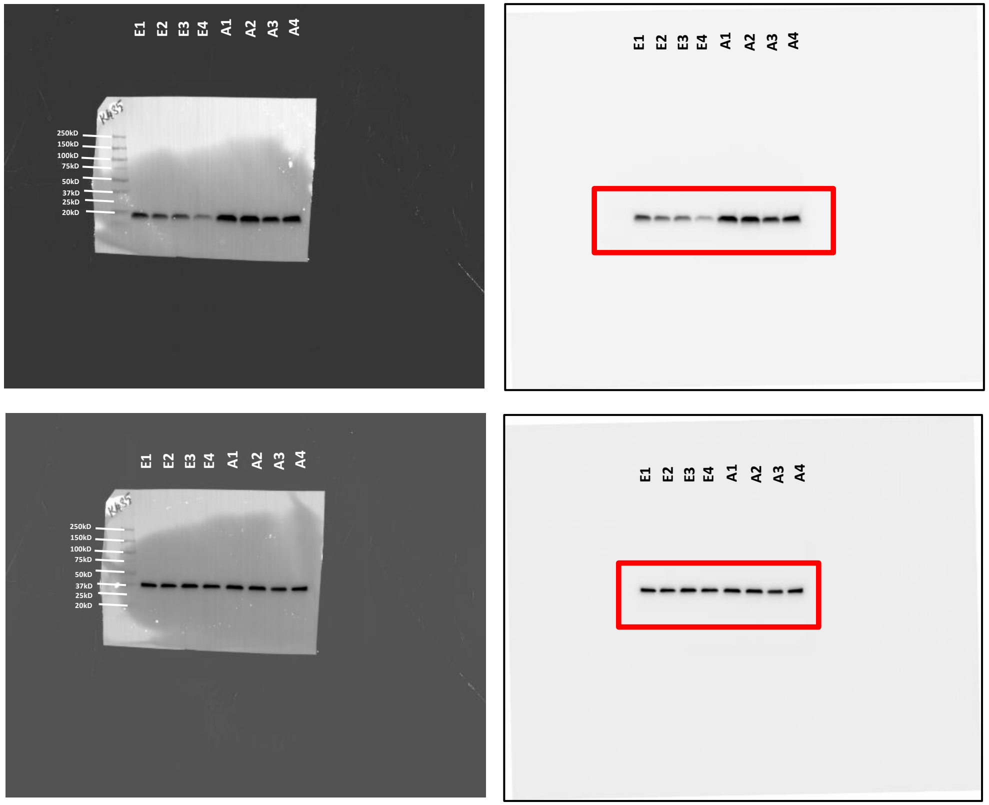

Area used for assembled the figure

NDUFB8:1 in 1000, Abcam
GAPDH: 1 in 5000, CST

Blot no:K485

Unedited blots associated with Figure 3A (SCaBER)

Overlaid with ladder **Raw image**

NDUFB8

GAPDH

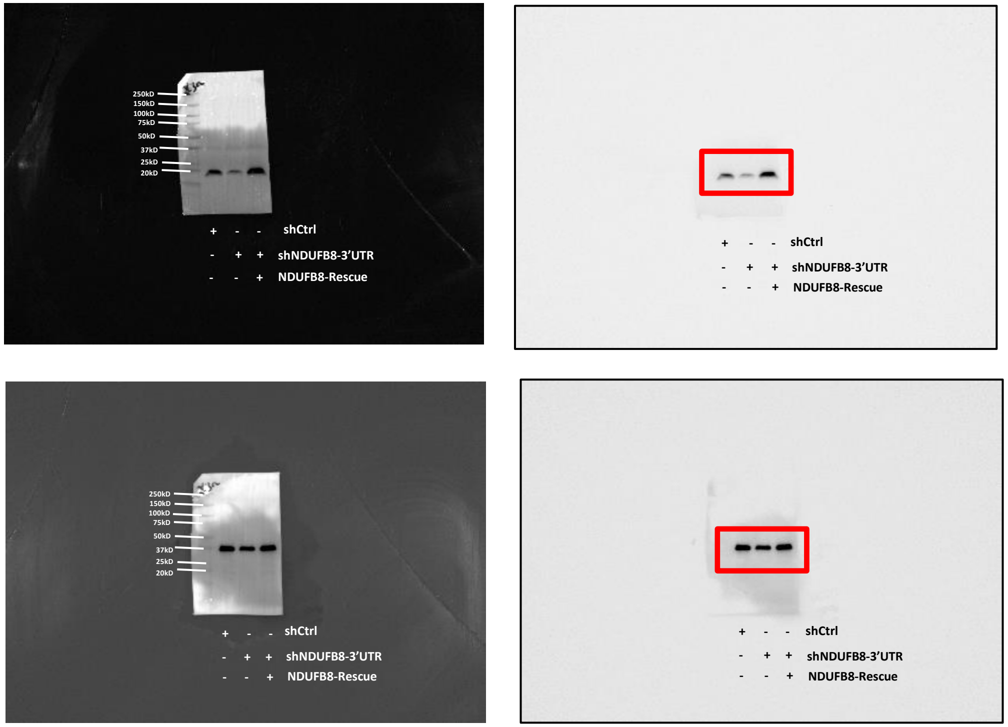

Area used for assembled the figure

NDUFB8:1 in 1000, Abcam
GAPDH: 1 in 5000, CST

Blot:263a

Unedited blots associated with Figure 3B (UM-UC-1)

Overlaid with ladder | **Raw image**

NDUFB8

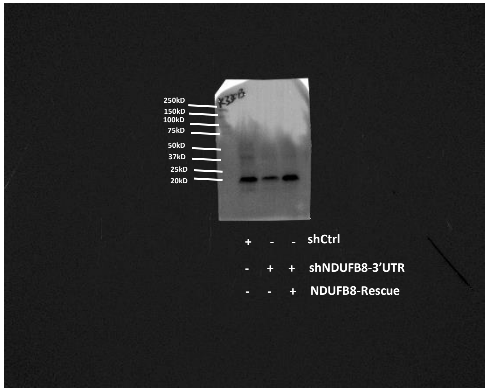

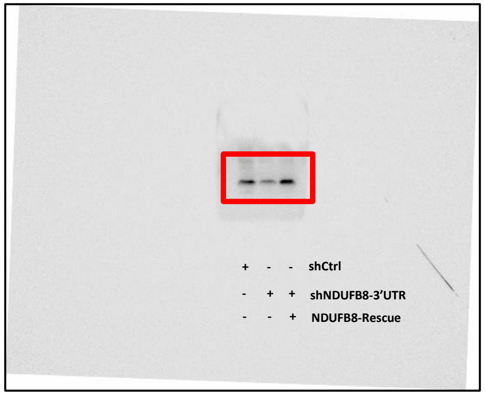

β-Actin

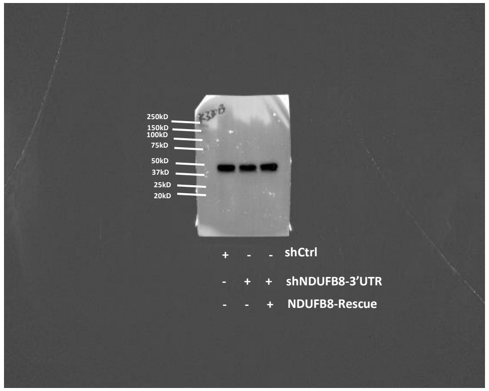

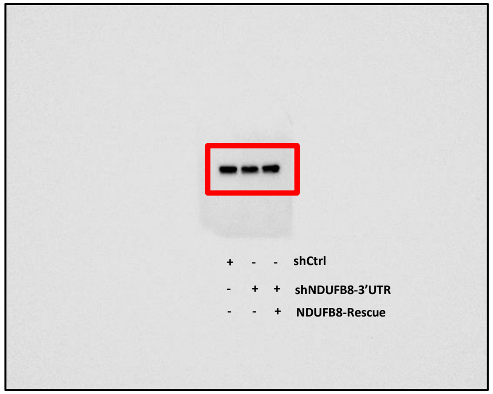

Area used for assembled the figure

NDUFB8:1 in 1000, Abcam
β-Actin: 1 in 5000, CST

Blot:K330b

Unedited blots associated with Figure 4B (Left panel: SCaBER, AA BLCA cell line)

Overlaid with ladder

Raw image

GLS1

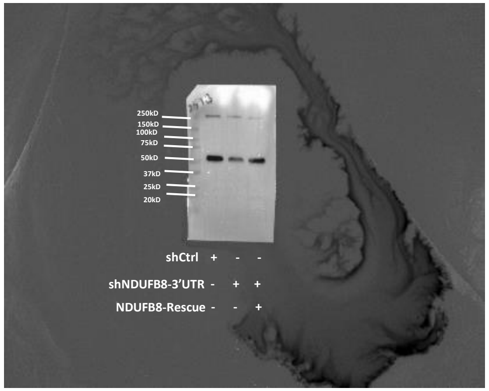

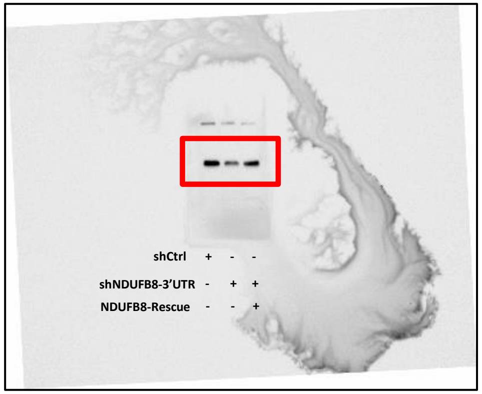

GAPDH

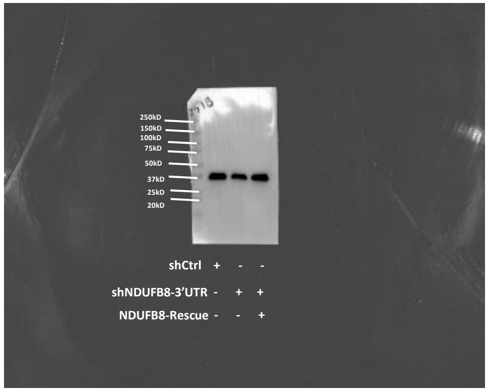

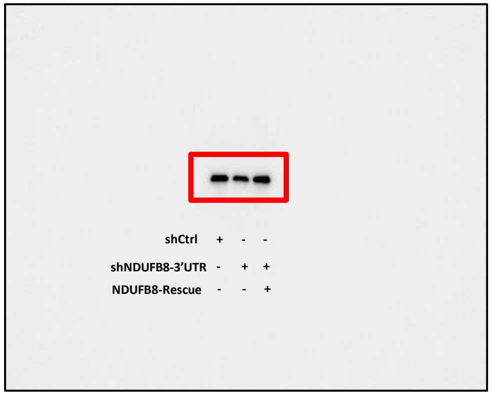

Area used for assembled the figure

GLS1:1 in 1000, CST

GAPDH: 1 in 5000, CST

Blot:259b

Unedited blots associated with Figure 4B (Middle panel: UM-UC-1, AA BLCA cell line)

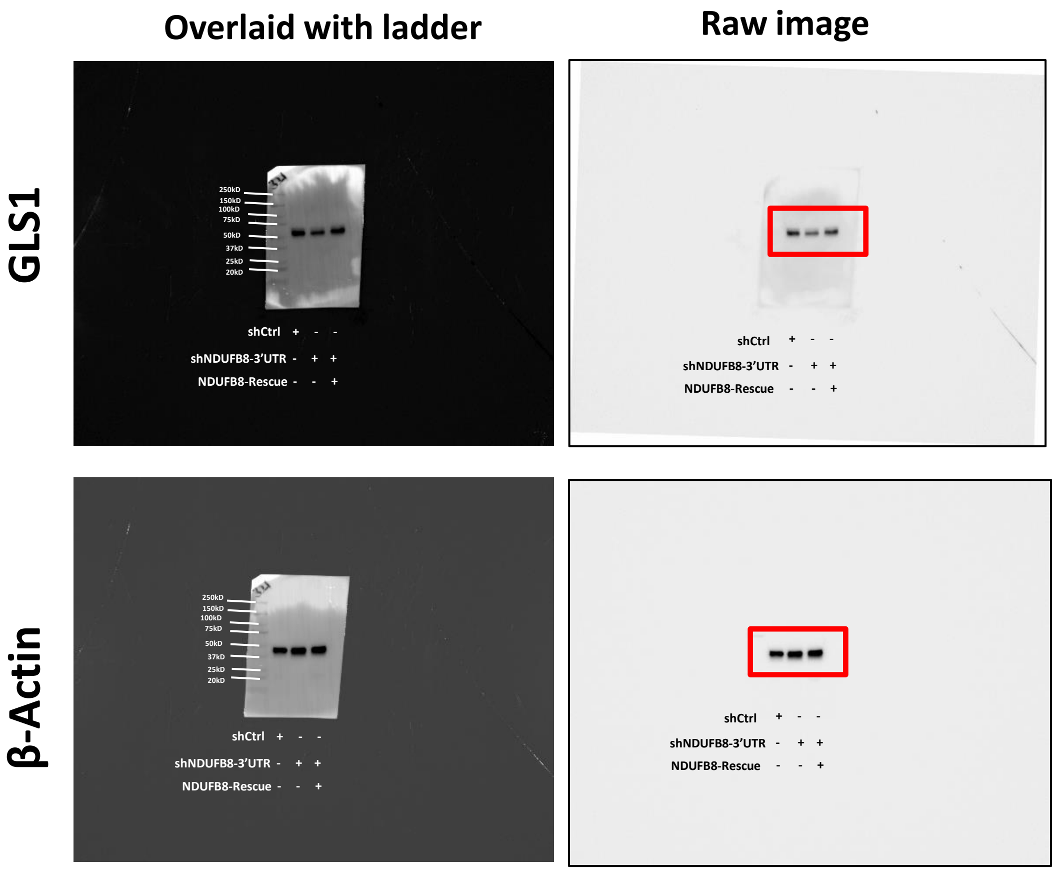

Area used for assembled the figure

GLS1: 1 in 1000, CST
β-Actin: 1 in 5000, CST

Blot: 321

Unedited blots associated with Figure 4B (Right panel: UM-UC-3, EA BLCA cell line)

Overlaid with ladder | Raw image

GLS1

GAPDH

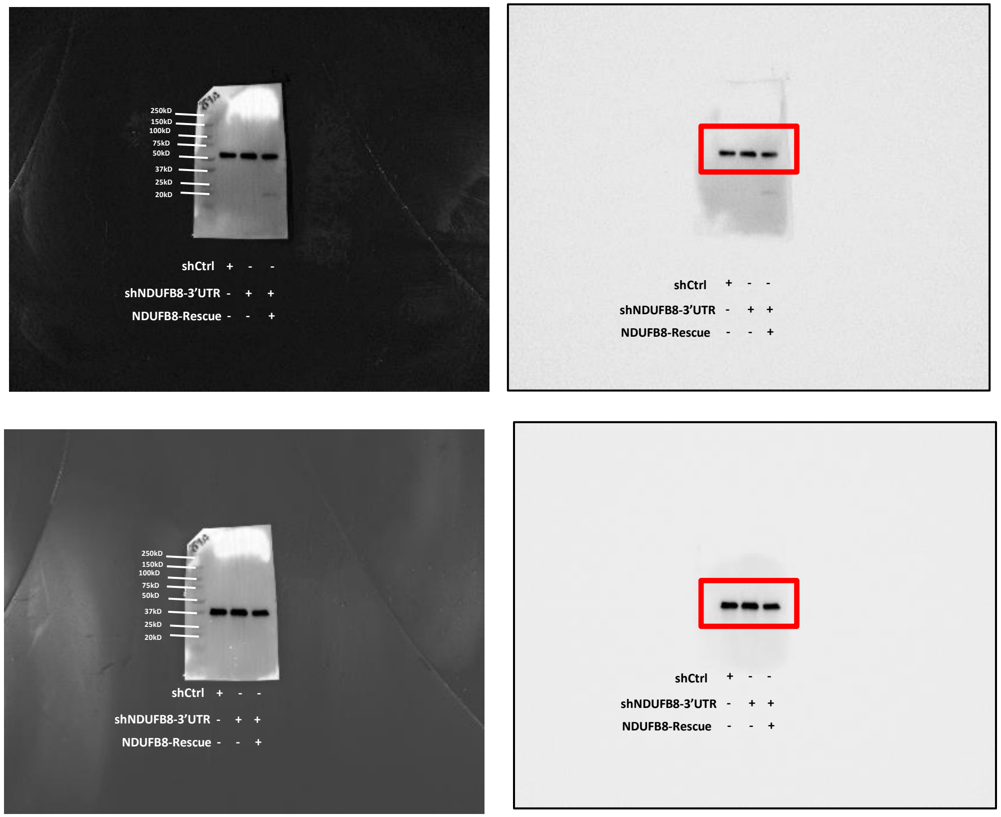

Area used for assembled the figure

GLS1:1 in 1000, CST
GAPDH: 1 in 5000, CST

Blot:259a

Unedited blots associated with Figure 4C (Left panel: SCaBER, AA BLCA cell line)

Overlaid with ladder | **Raw image**

GLS1

GAPDH

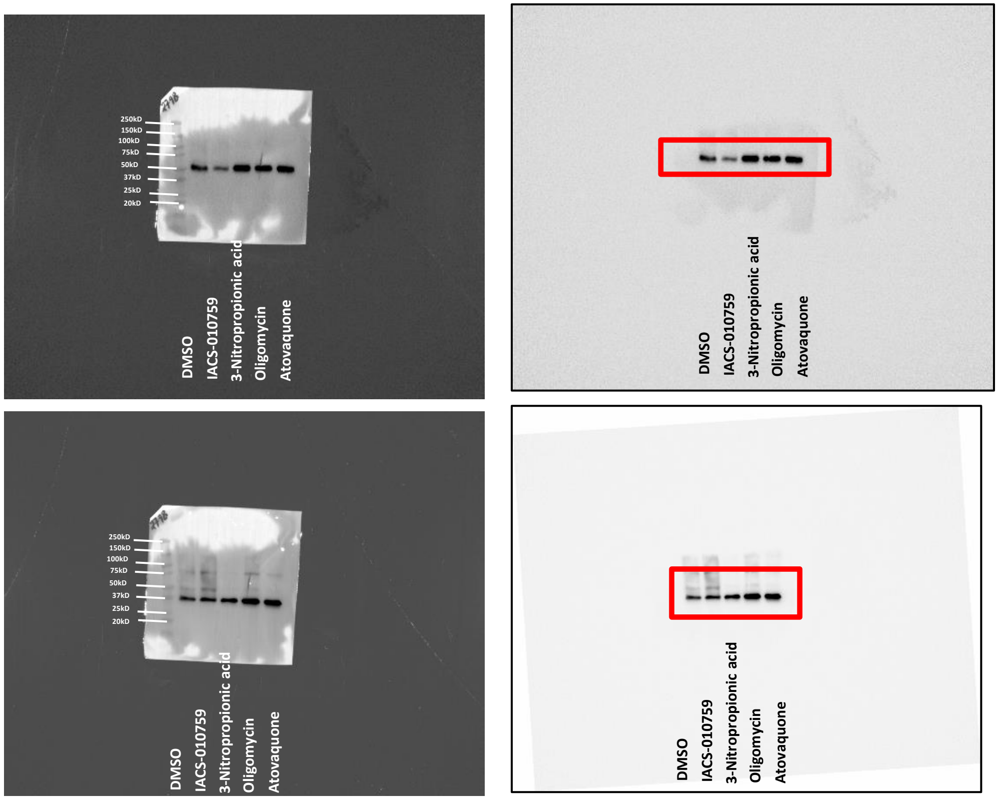

Area used for assembled the figure

GLS1: 1 in 1000, CST
GAPDH: 1 in 5000, CST

Blot: 279B

Unedited blots associated with Figure 4C (Middle panel: UM-UC-1, AA BLCA cell line)

Overlaid with ladder

Raw image

GLS1

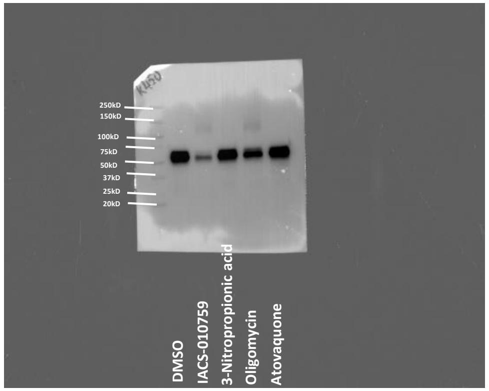

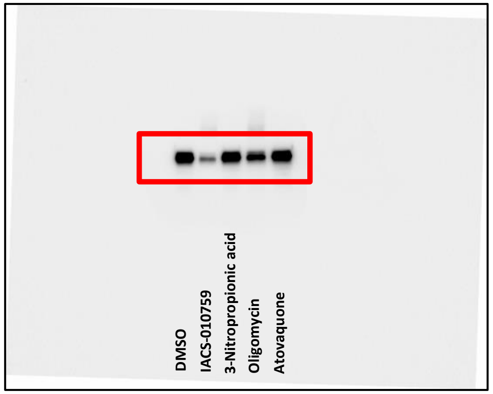

GAPDH

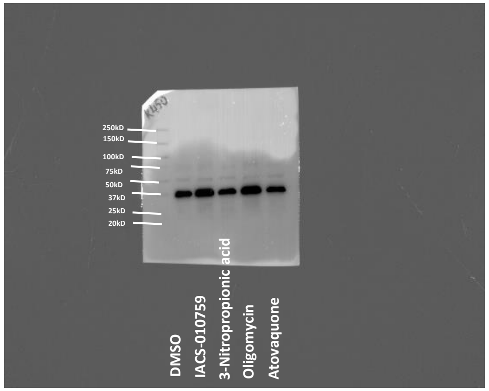

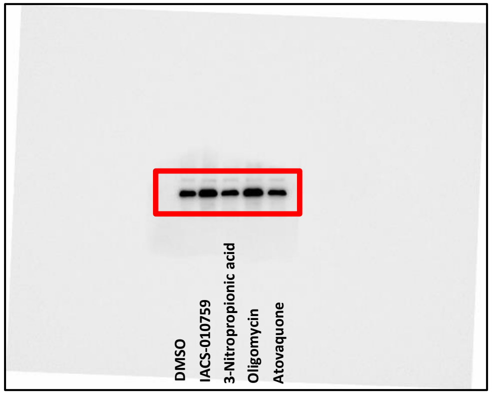

Area used for assembled the figure

GLS1: 1 in 1000, CST
GAPDH: 1 in 5000, CST

Blot: K450

Unedited blots associated with Figure 4C (Right panel: UM-UC-3, EA BLCA cell lines)

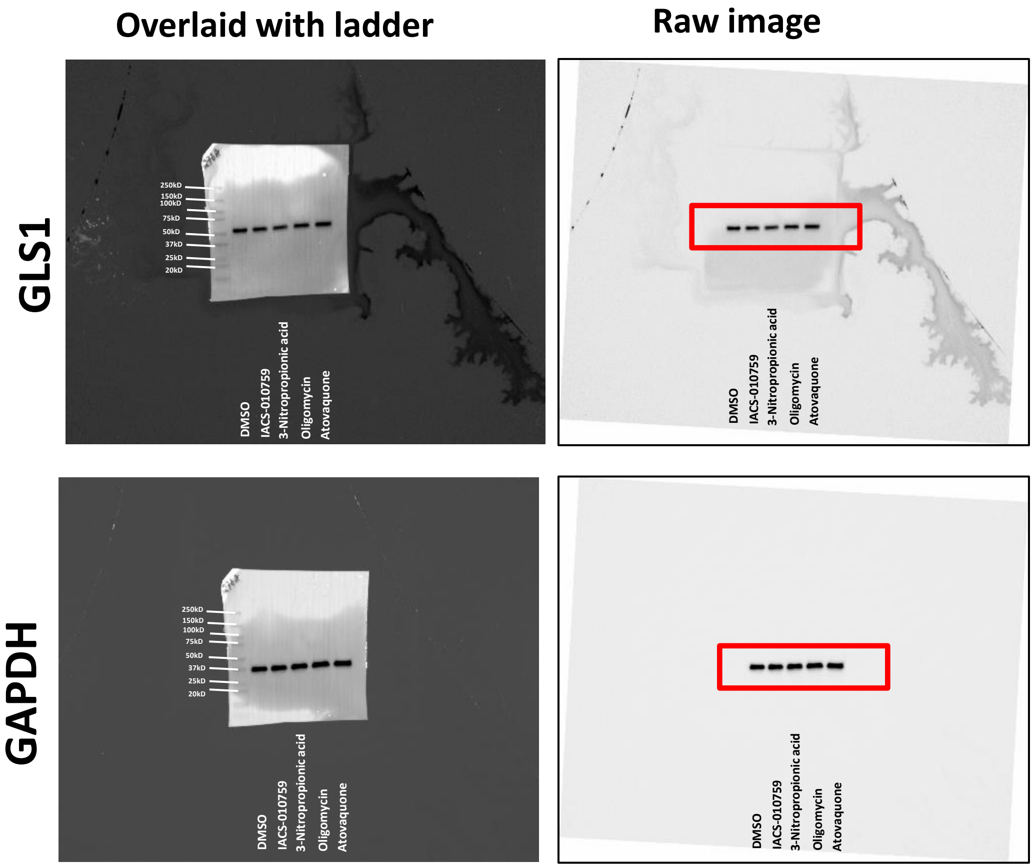

Area used for assembled the figure

GLS1: 1 in 1000, CST
GAPDH: 1 in 5000, CST

Blot: 276A

Unedited blots associated with Supplementary Figure 3B

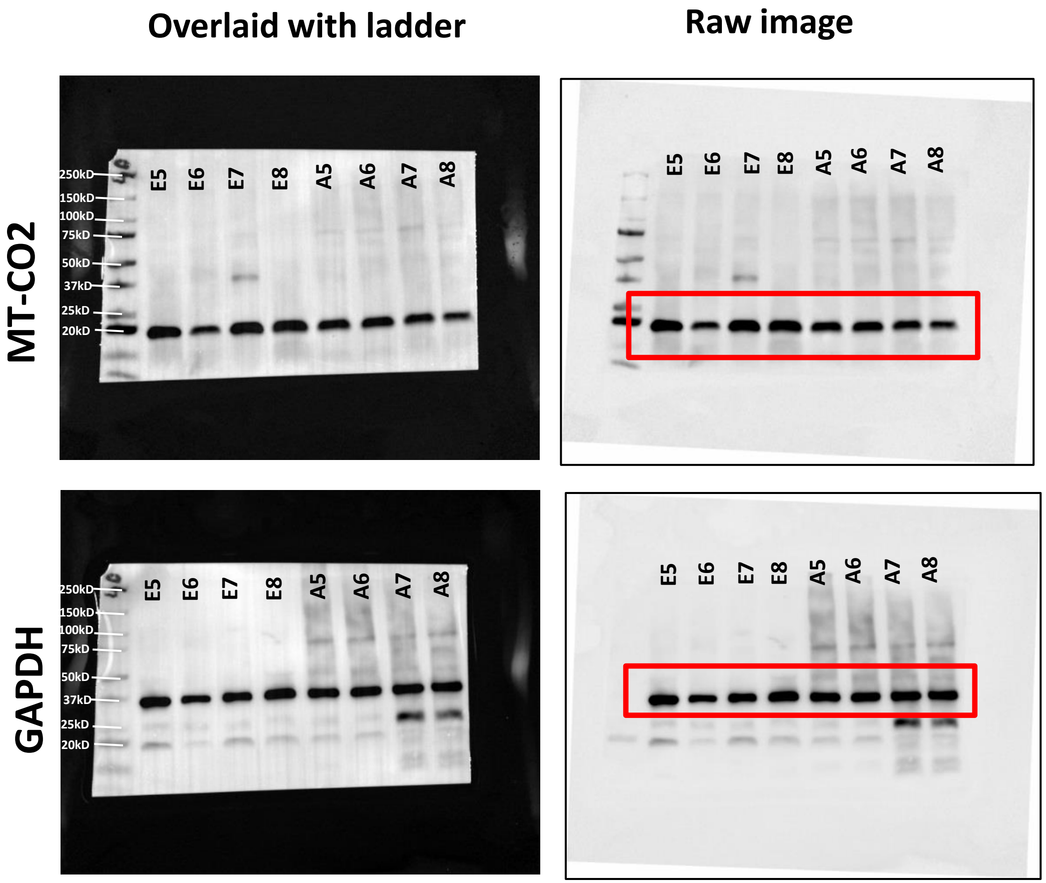

Area used for assembled the figure

MT-CO2:1 in 1000, Abcam
GAPDH: 1 in 5000, CST

Blot no:40

Note: For lane 4 (E7), lane 5-8 (A5, A6, A7, A8) same patient sample lysates was run on different gels for targeting, MT-CO2, and SDHB (refer to slide 13). Remaining lanes from MT-CO2 blot are from different set of patients.

Unedited blots associated with Supplementary Figure 3B

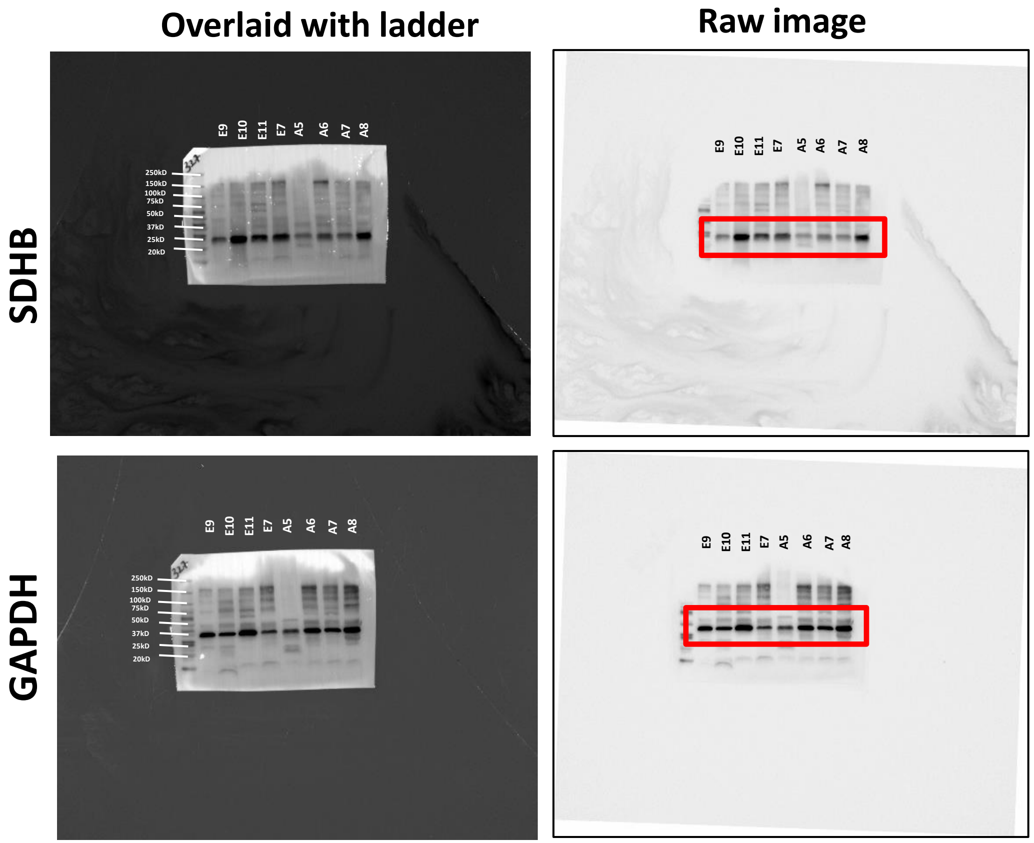

Area used for assembled the figure

SDHB:1 in 1000, Abcam
GAPDH: 1 in 5000, CST

Blot no: 327

Note: For lane 4 (E7), lane 5-8 (A5, A6, A7, A8) same patient sample lysates was run on different gels for targeting, MT-CO2 (refer to slide12), and SDHB. Remaining lanes from SDHB blot are from different set of patients.

Unedited blots associated with Supplementary Figure 3B

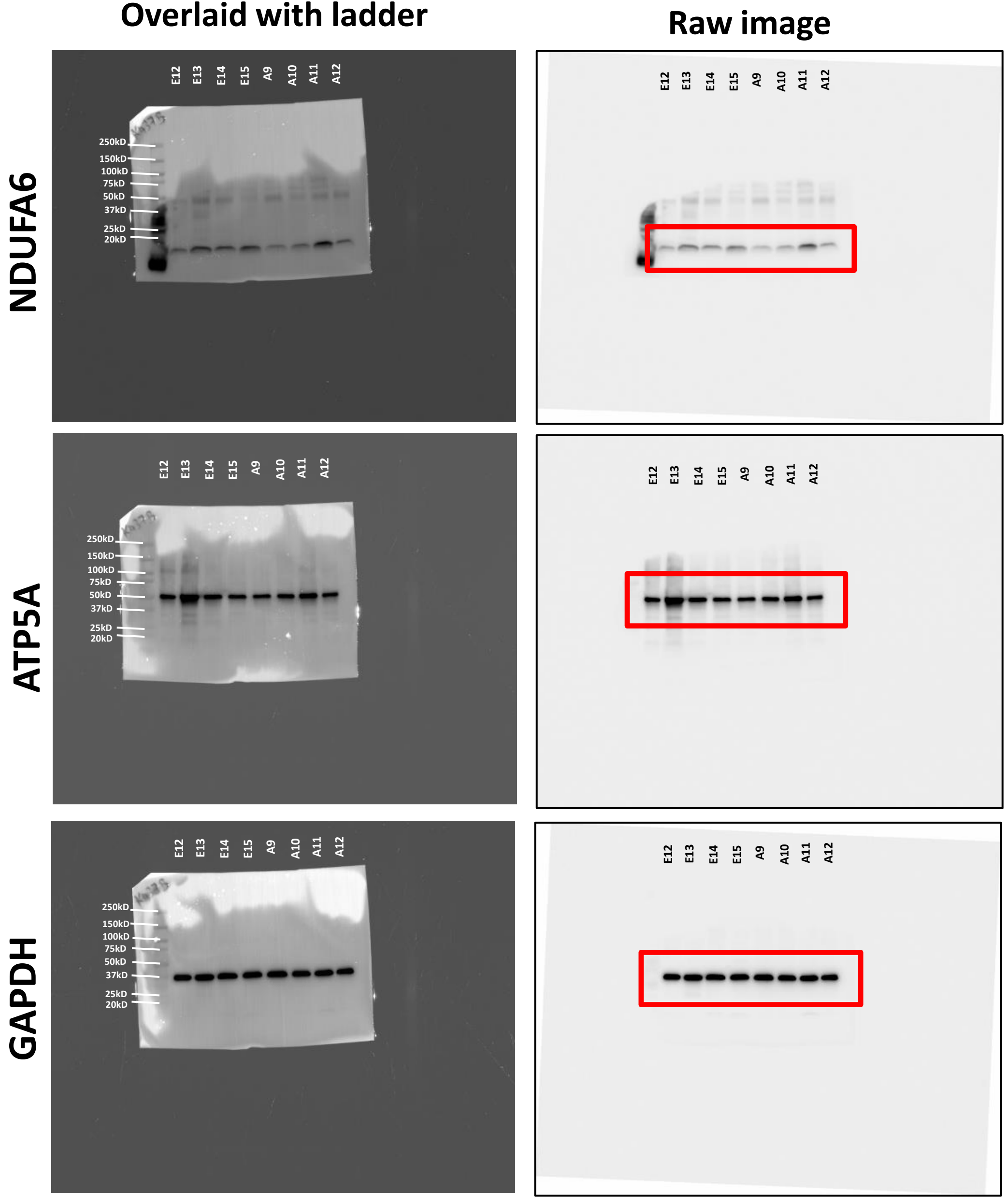

Area used for assembled the figure

NDUFA6:1 in 1000, Novus Biological
ATP5A:1 in 1000, Abcam
GAPDH: 1 in 5000, CST

Blot no: K437B

Note: Same patient (E12, E13, E14, E15, A9, A10, A11, A12) sample lysates were for targeting NDUFA6, ATP5A and NDUFV1, UQCRC2 (refer to slide 15). NDUFA6 and ATP5A run on blot no K437B, NDUFV1 and UQCRC2 run on gel no K437A (slide 15).

Unedited blots associated with Supplementary Figure 3B

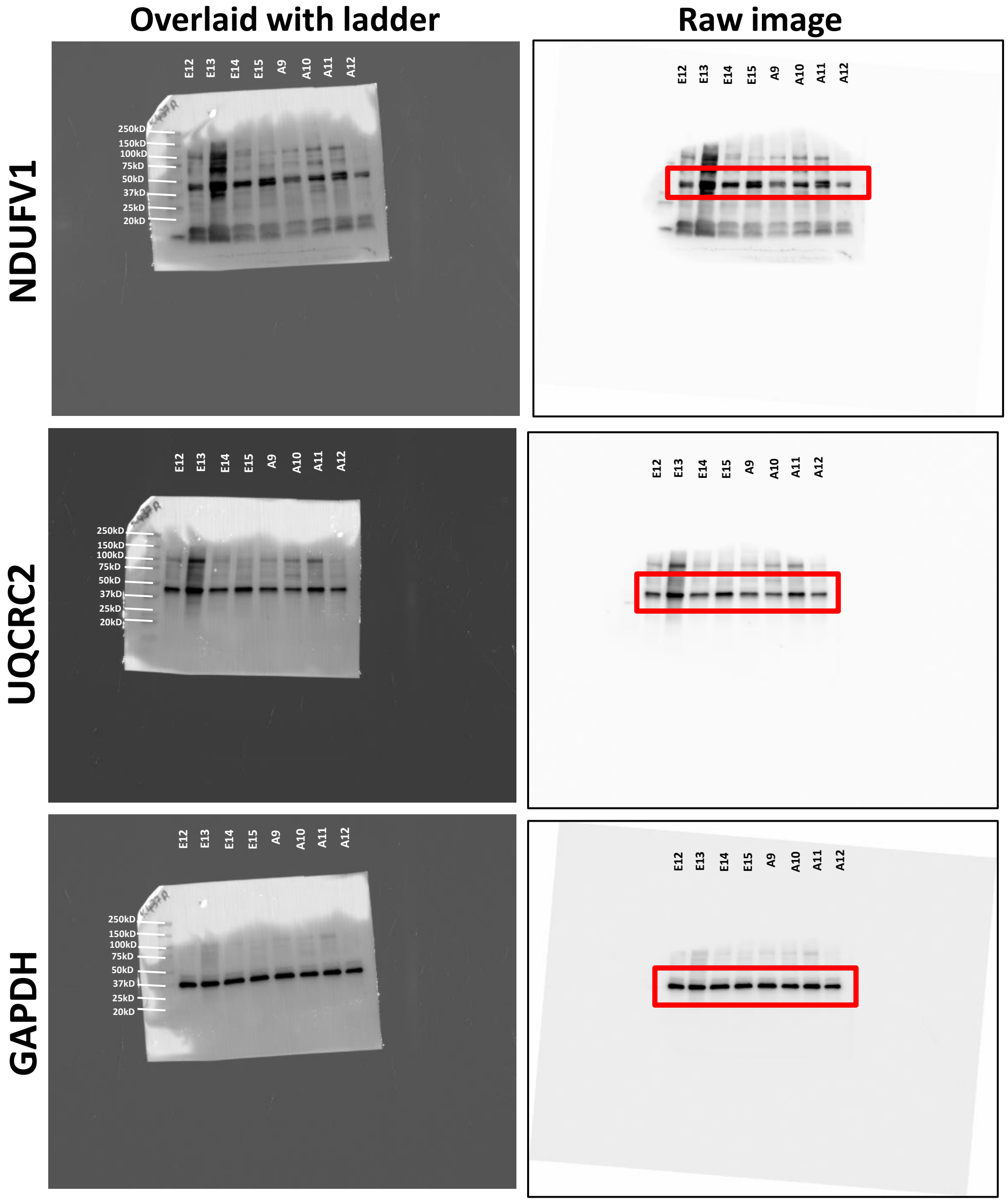

Area used for assembled the figure

NDUFV1:1 in 1000, Novus Biological
UQCRC2:1 in 1000, Abcam
GAPDH: 1 in 5000, CST

Blot no: K437A

Note: Same patient (E12, E13, E14, E15, A9, A10, A11, A12) sample lysates were for targeting NDUFA6, ATP5A (refer to slide 14) and NDUFV1, UQCRC2. NDUFA6 and ATP5A run on blot no K437B (slide 14), NDUFV1 and UQCRC2 run on gel no K437A.

Unedited blots associated with Supplementary Figure 3C

Overlaid with ladder

Raw image

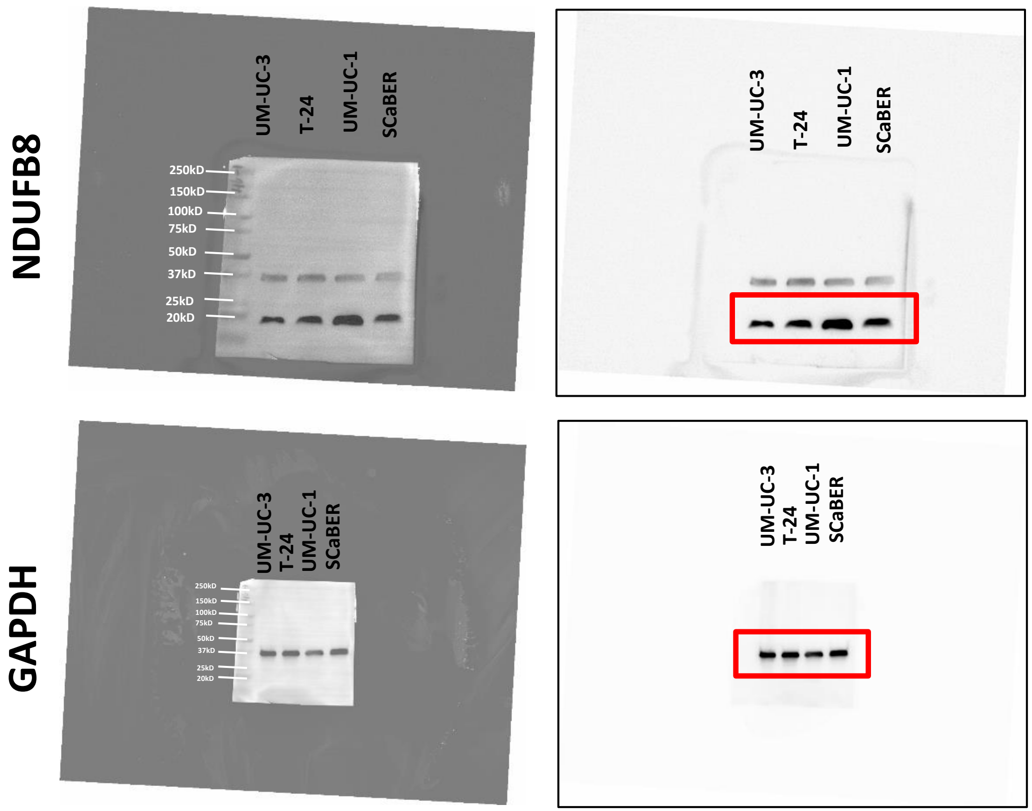

Area used for assembled the figure

NDUFB8:1 in 1000, Abcam
GAPDH: 1 in 5000, CST

Blot: 46-1

Unedited blots associated with Supplementary Figure 4A (SCaBER)

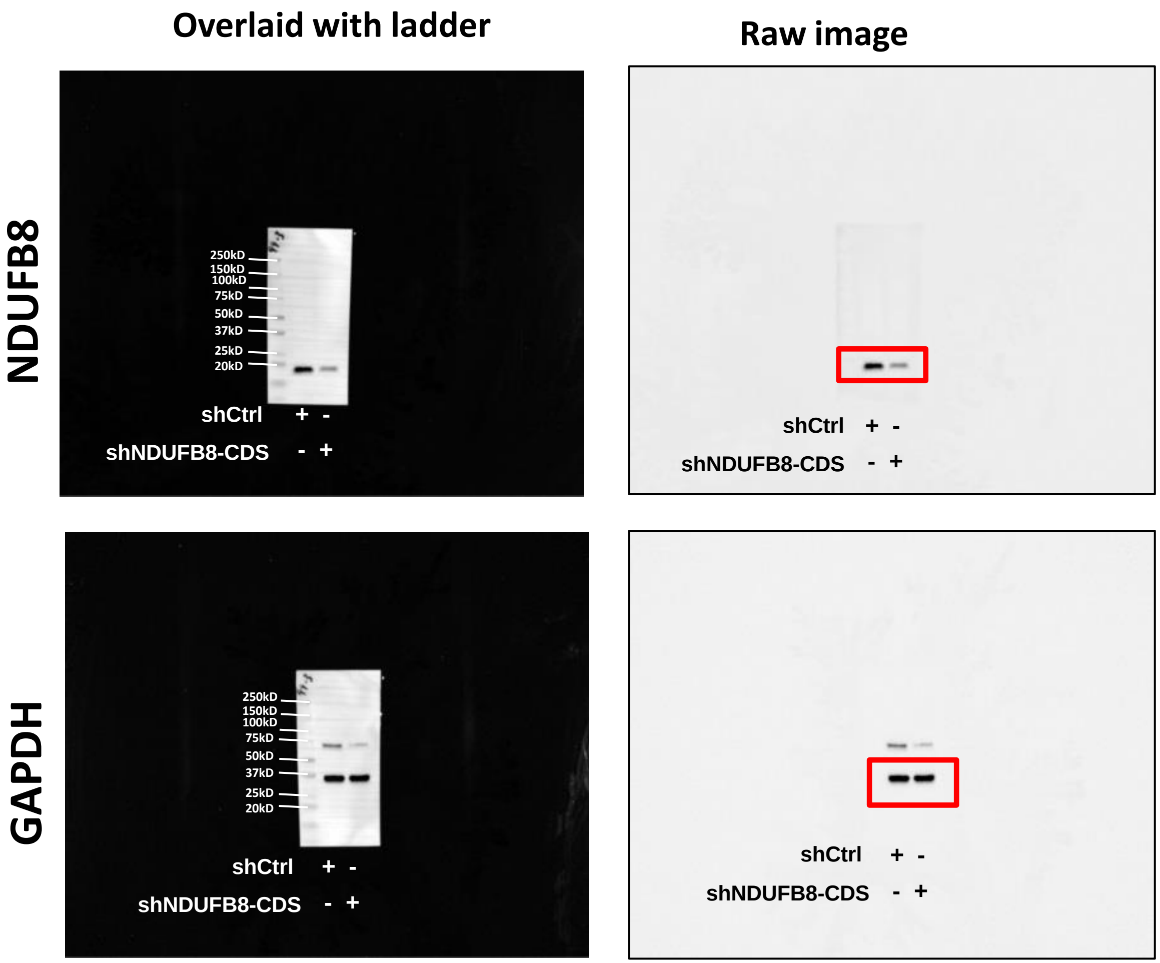

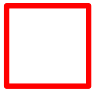

NDUFB8:1 in 1000, Abcam
GAPDH: 1 in 5000, CST

BLOT #99-3

Unedited blots associated with Supplementary Figure 4E (UM-UC-3)

Overlaid with ladder | Raw image

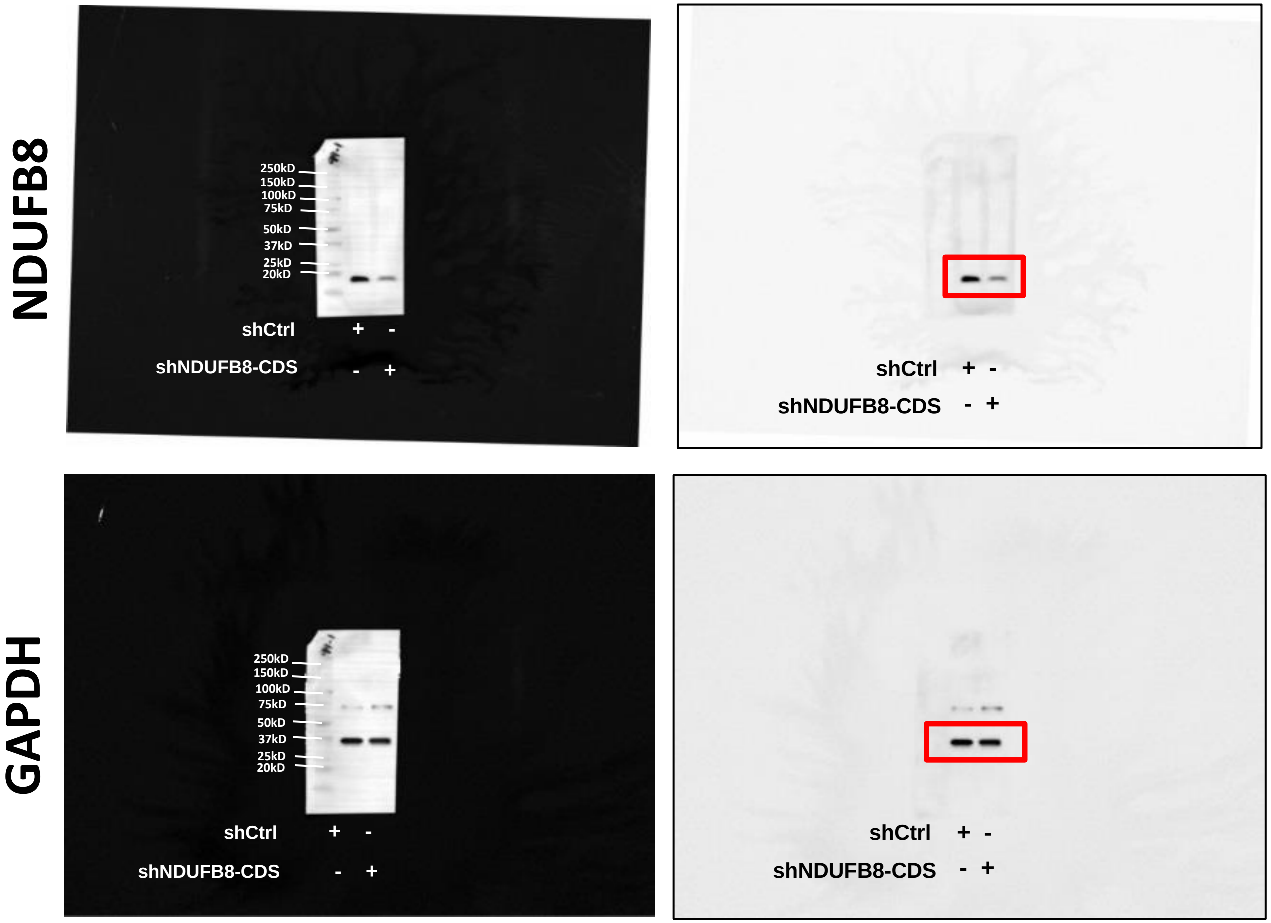

Area used for assembled the figure

NDUFB8:1 in 1000, Abcam
GAPDH: 1 in 5000, CST

BLOT #99-1

Unedited blots associated with Supplementary Figure 4I (UM-UC-3)

Overlaid with ladder

Raw image

NDUFB8

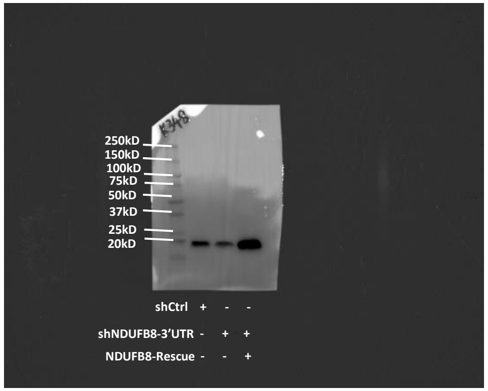

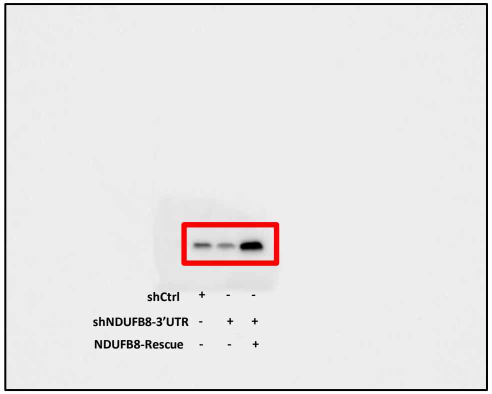

GAPDH

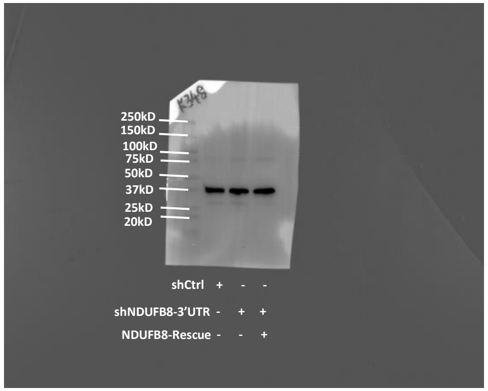

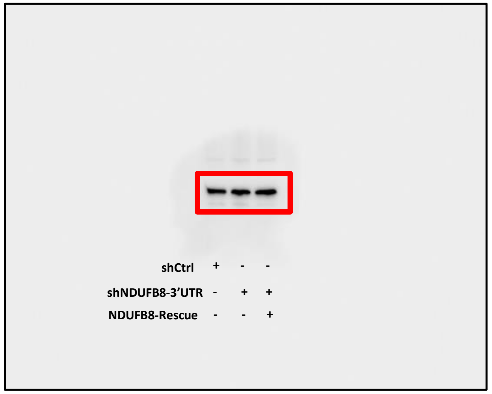

Area used for assembled the figure

NDUFB8:1 in 1000, Abcam
GAPDH: 1 in 5000, CST

Blot:K348

Unedited blots associated with Supplementary Figure: 6A (SCaBER)

Overlaid with ladder | **Raw image**

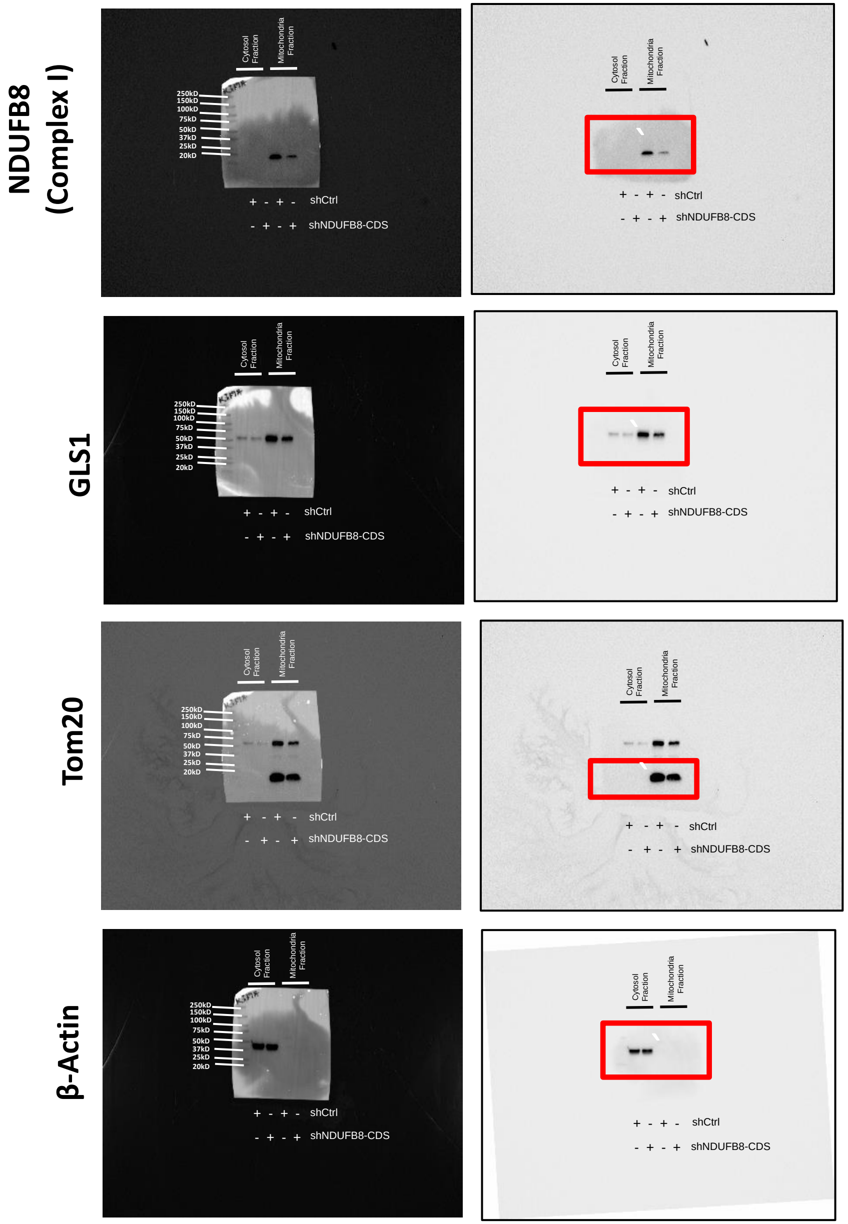

Area used for assembled the figure

NDUFB8: 1 in 1000, Abcam
GLS1: 1 in 1000, CST
Tom20: 1 in 1000, CST
β-Actin: 1 in 5000, CST

Blot: K339A

Unedited blots associated with Supplementary Figure: 6D

Overlaid with ladder | Raw image

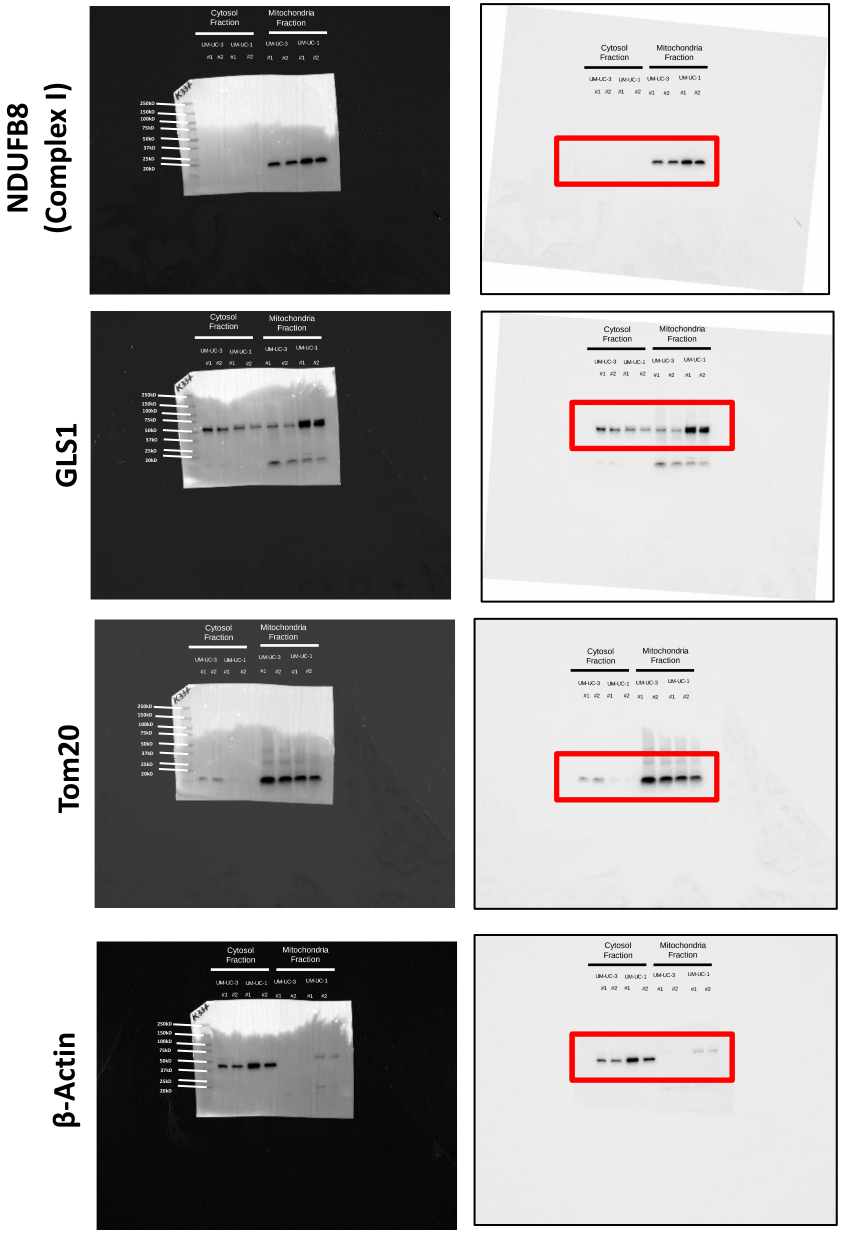

Area used for assembled the figure

NDUFB8: 1 in 1000, Abcam
GLS1: 1 in 1000, CST
Tom20: 1 in 1000, CST
β-Actin: 1 in 5000, CST

Blot: K337

Note: #1,and #2 are replicates

Unedited blots associated with Supplementary Figure: 6E (UM-UC-1)

	Overlaid with ladder	Raw image
NDUFB8	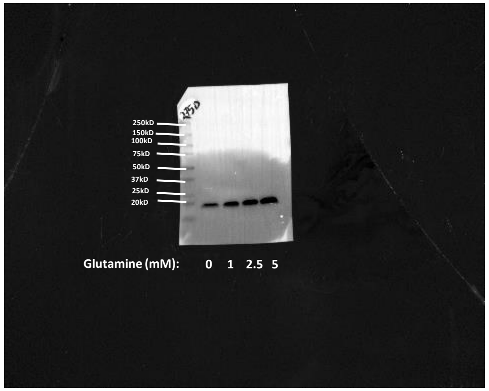	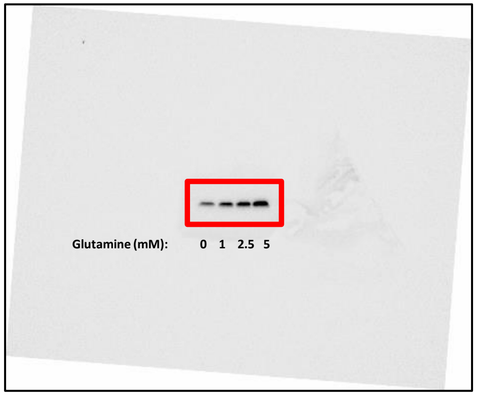
GAPDH	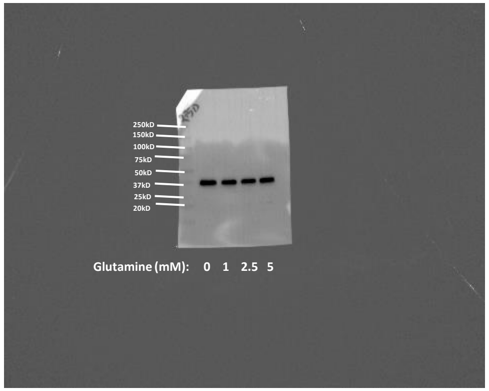	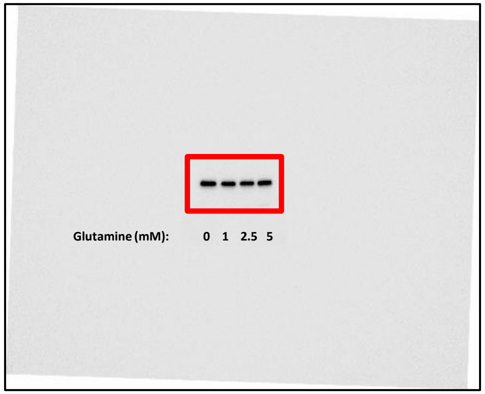

Area used for assembled the figure

NDUFB8:1 in 1000, Abcam
GAPDH: 1 in 5000, CST

Blot: 275D

Unedited blots associated with Supplementary Figure: 6F (SCaBER)

Overlaid with ladder | **Raw image**

NDUFB8

GAPDH

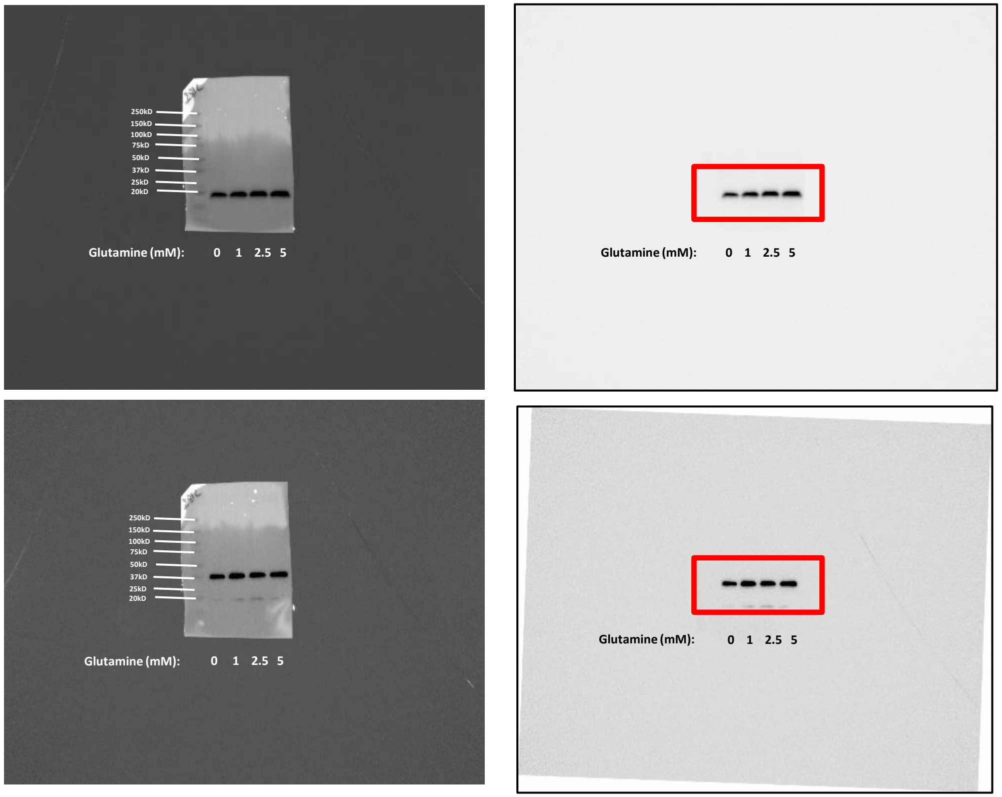

Area used for assembled the figure

NDUFB8:1 in 1000, Abcam
GAPDH: 1 in 5000, CST

Blot: 281C

Unedited blots associated with Supplementary Figure: 6G (UM-UC-1)

Overlaid with ladder | **Raw image**

GLS1

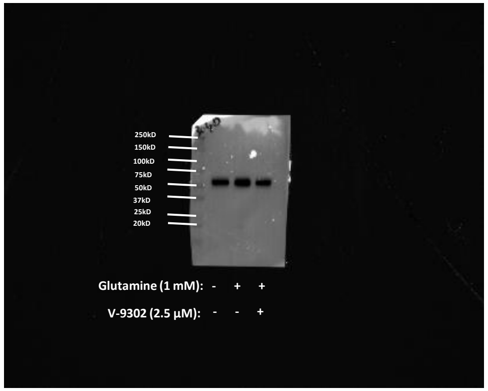

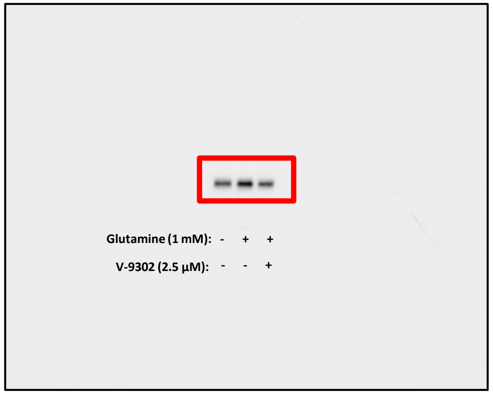

NDUFB8

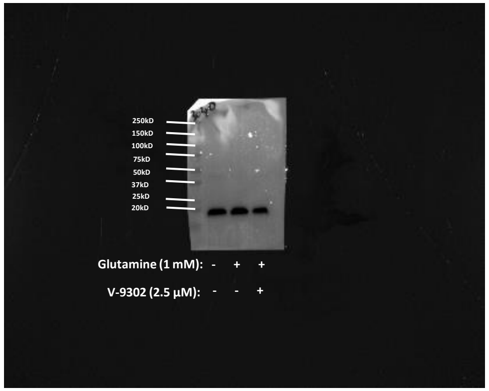

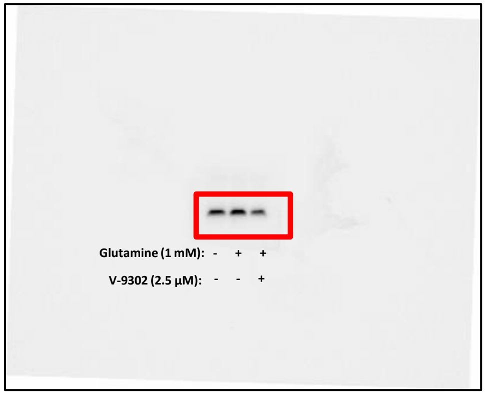

β-Actin

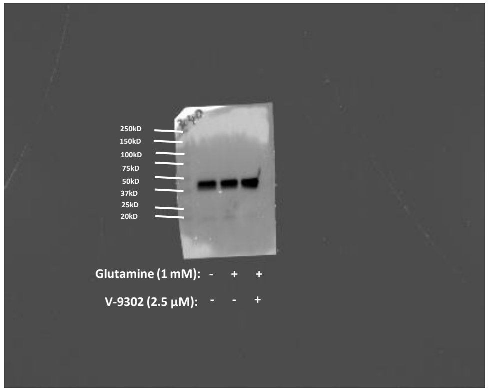

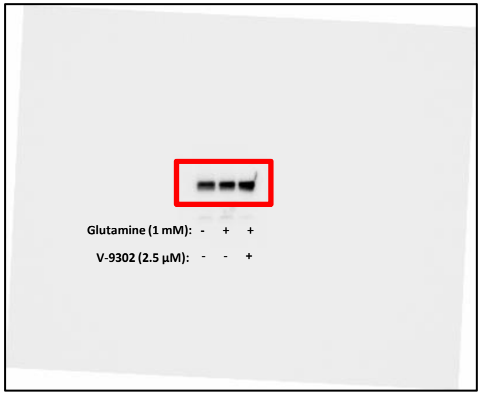

Area used for assembled the figure

NDUFB8: 1 in 1000, Abcam
GLS1: 1 in 1000, CST
β-Actin: 1 in 5000, CST

Blot: 304D

Unedited blots associated with Supplementary Figure: 6H (SCaBER)

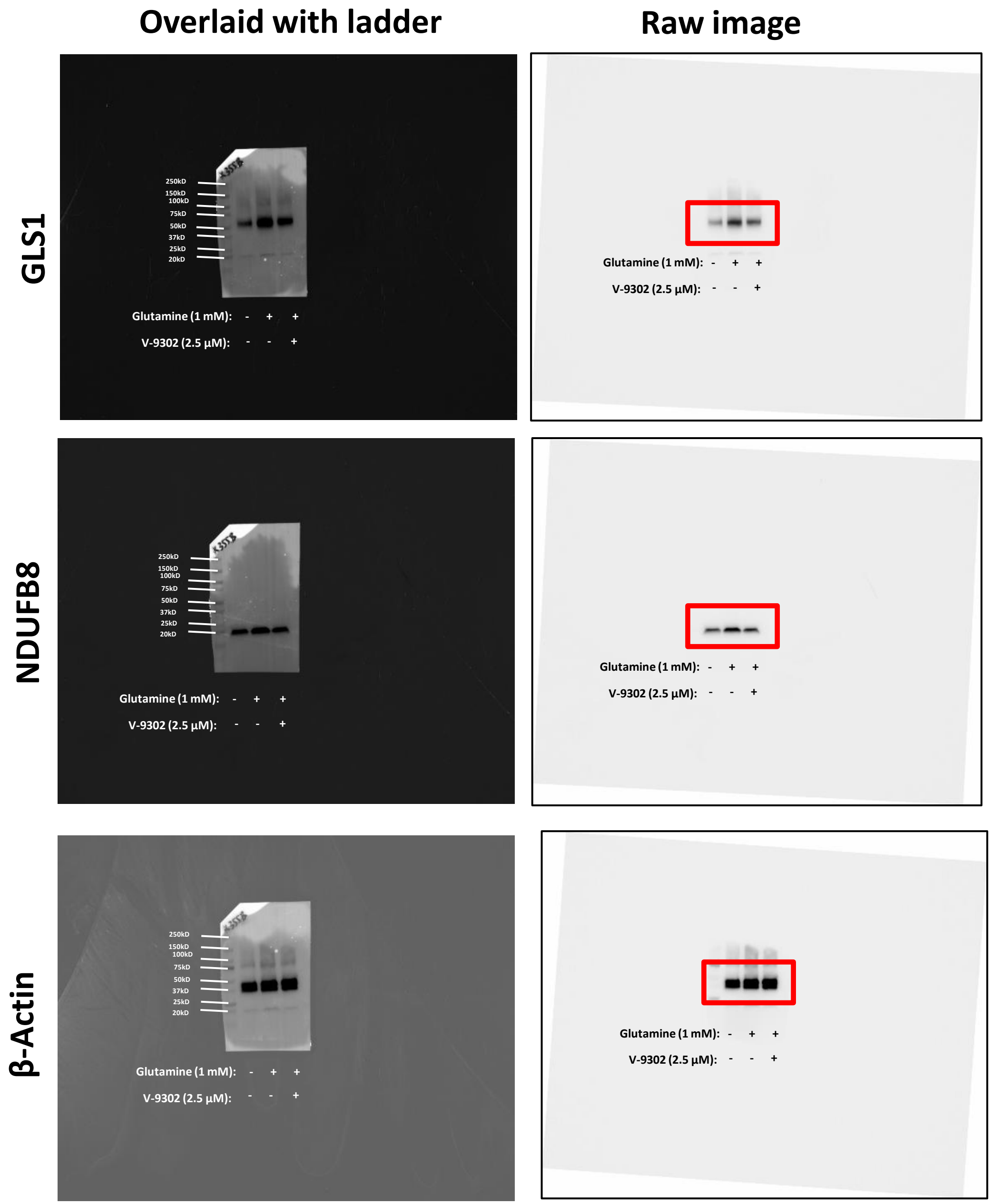

Area used for assembled the figure

NDUFB8: 1 in 1000, Abcam
GLS1: 1 in 1000, CST
β-Actin: 1 in 5000, CST

Blot: 355B

Unedited blots associated with Supplementary Figure 7A (Left panel: SCaBER)

Overlaid with ladder | **Raw image**

GLS1

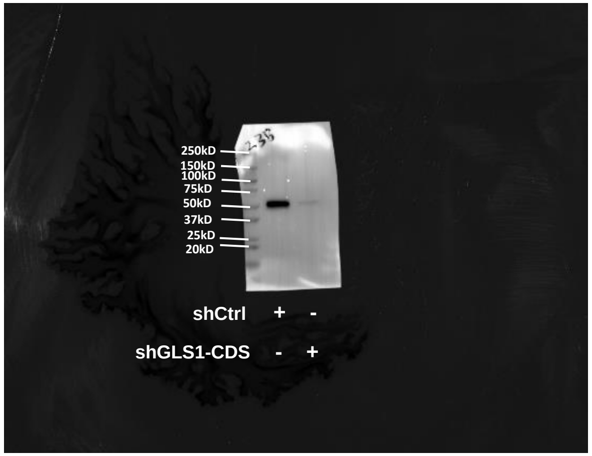

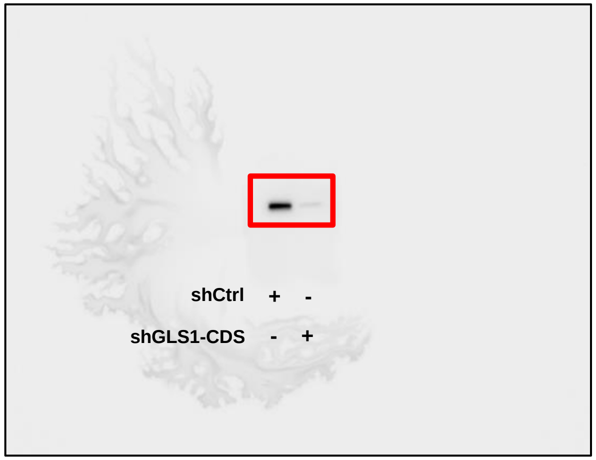

GAPDH

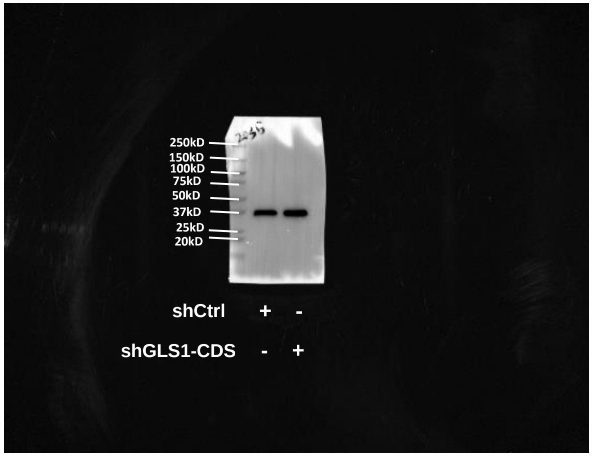

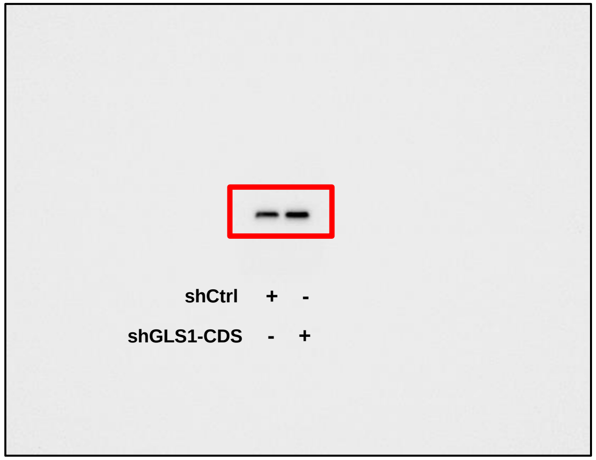

Area used for assembled the figure

GLS1: 1 in 1000, CST
GAPDH: 1 in 5000, CST

Blot:203b

Unedited blots associated with Supplementary Figure 7A (Right panel: UM-UC-3)

Overlaid with ladder

Raw image

GLS1

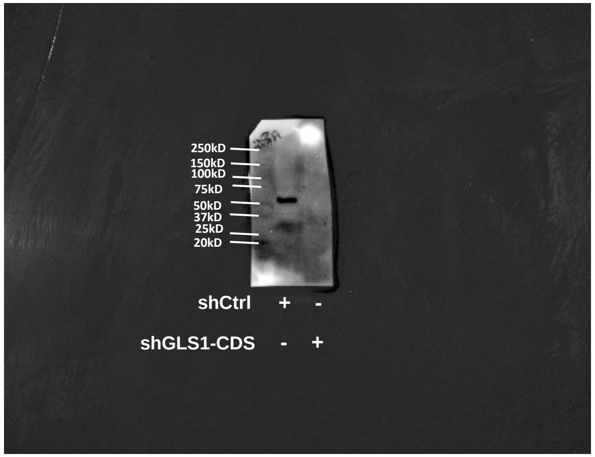

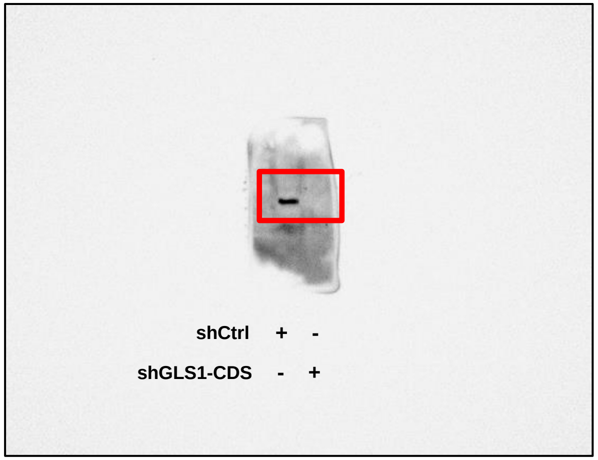

GAPDH

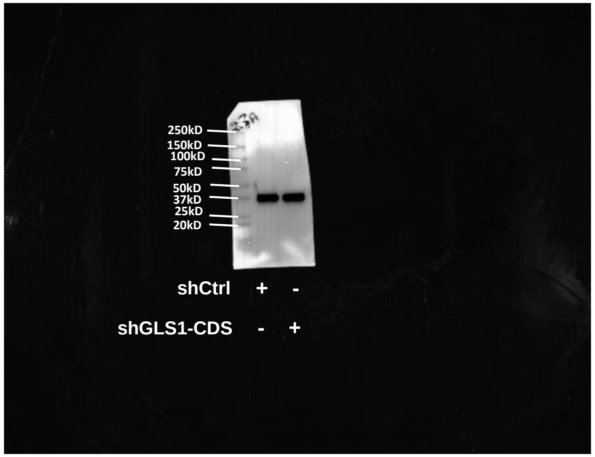

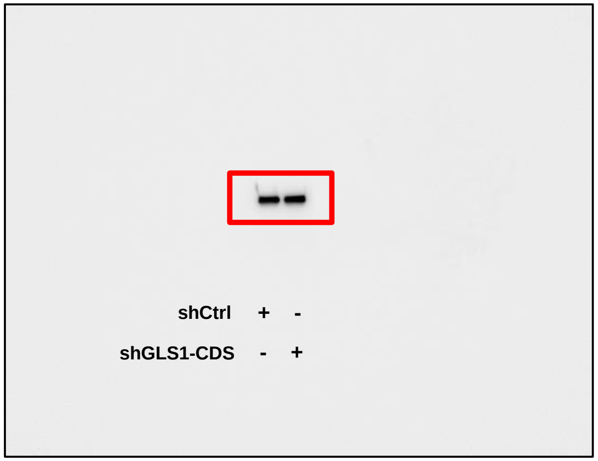

Area used for assembled the figure

GLS1: 1 in 1000, CST
GAPDH: 1 in 5000, CST

Blot:203a

Raw images associated with Supplementary Figure 7B and 7C

Supplementary Figure 7B

SCaBER

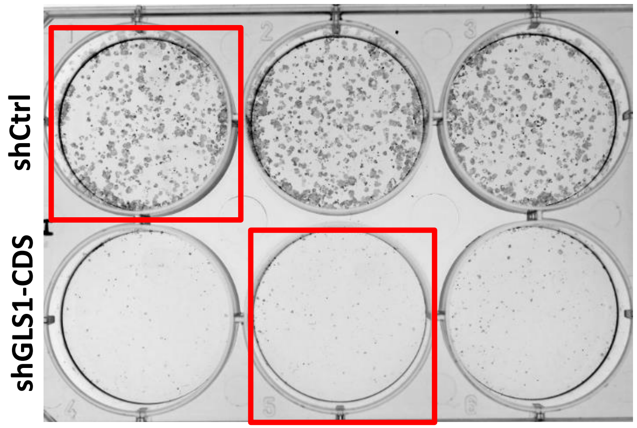

Supplementary Figure 7C

UM-UC-3

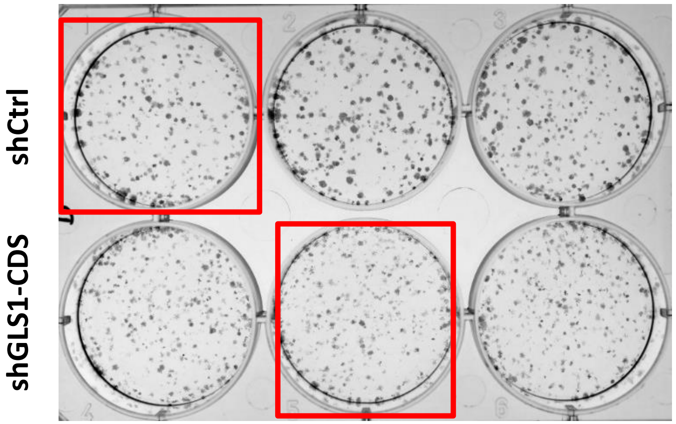

Area used for assembled the figure
